# Supplementary material for: Rbfox1 is required for myofibril development and maintaining fiber type–specific isoform expression in Drosophila muscles
Source: Life Sci Alliance. 2022 Jan 7;5(4):e202101342. doi: 10.26508/lsa.202101342 (PMC8742874; doi:10.26508/lsa.202101342)
Supplement: Supplementary file 2 [file LSA-2021-01342_SdataFS1.pdf]

Raw data used to generate plots

Figure panel

		Exon7		Exon12		Exon14/15		Exon17/18/19		
		Skipped	Included	Skipped	Included	Skipped	Included	xon17+18+1	exon17+19	exon19
S1B	IFMA	0.733032	0.266968	0.873016	0.126984	0.833333	0.166667	0	0.727273	0.272727
	AretIRA	0.83871	0.16129	0.916084	0.083916	0.740864	0.259136	0	0.864662	0.135338
	SalmA	0.661017	0.338983	0.688312	0.311688	0.545455	0.454545	0.060895	0.869923	0.069182
	TDTA	0.974684	0.025316	0.902439	0.097561	0.695793	0.304207	0.044888	0.859279	0.095833
	legA	0.645161	0.354839	0.915789	0.084211	0.639296	0.360704	0.012591	0.881349	0.106061

	IFM	TDT	Abd
S1C	1	1.315947	0.873964
	1	1.175008	0.75623
	1		

	w-	27286	KK101518	Dcr27286
S1E	1	1.607687	0.778339	0.778218
	1	1.328186	0.688763	0.677529
	1	1.031236	0.661546	0.592031

Original RT-PCR gels

Rbfox1 levels in Rbfox1KD hairpins

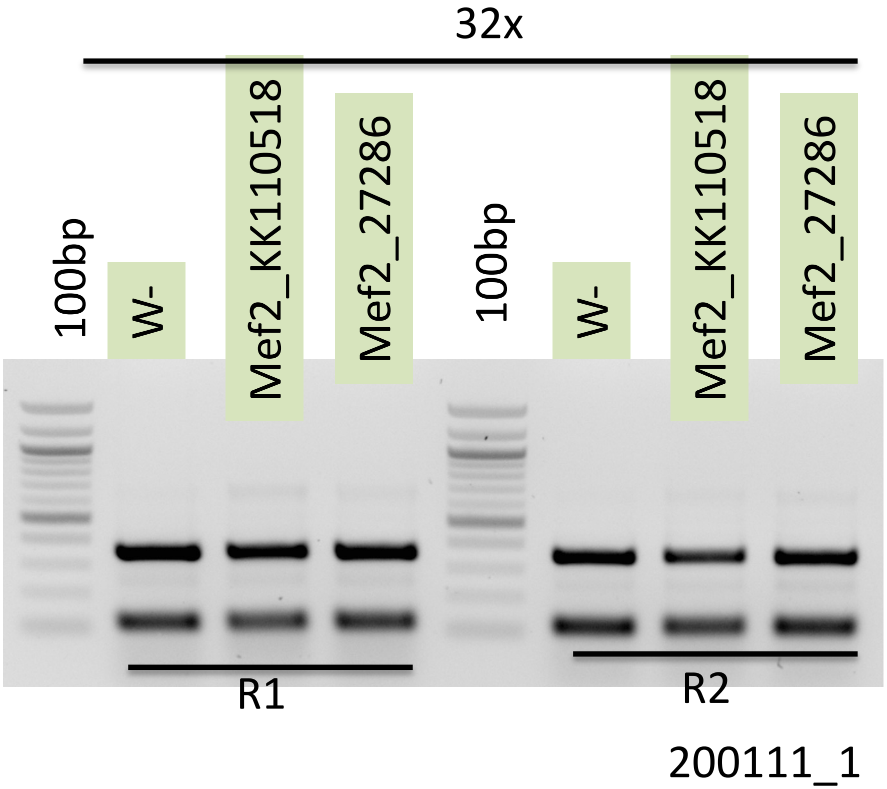

Tested in IFMs

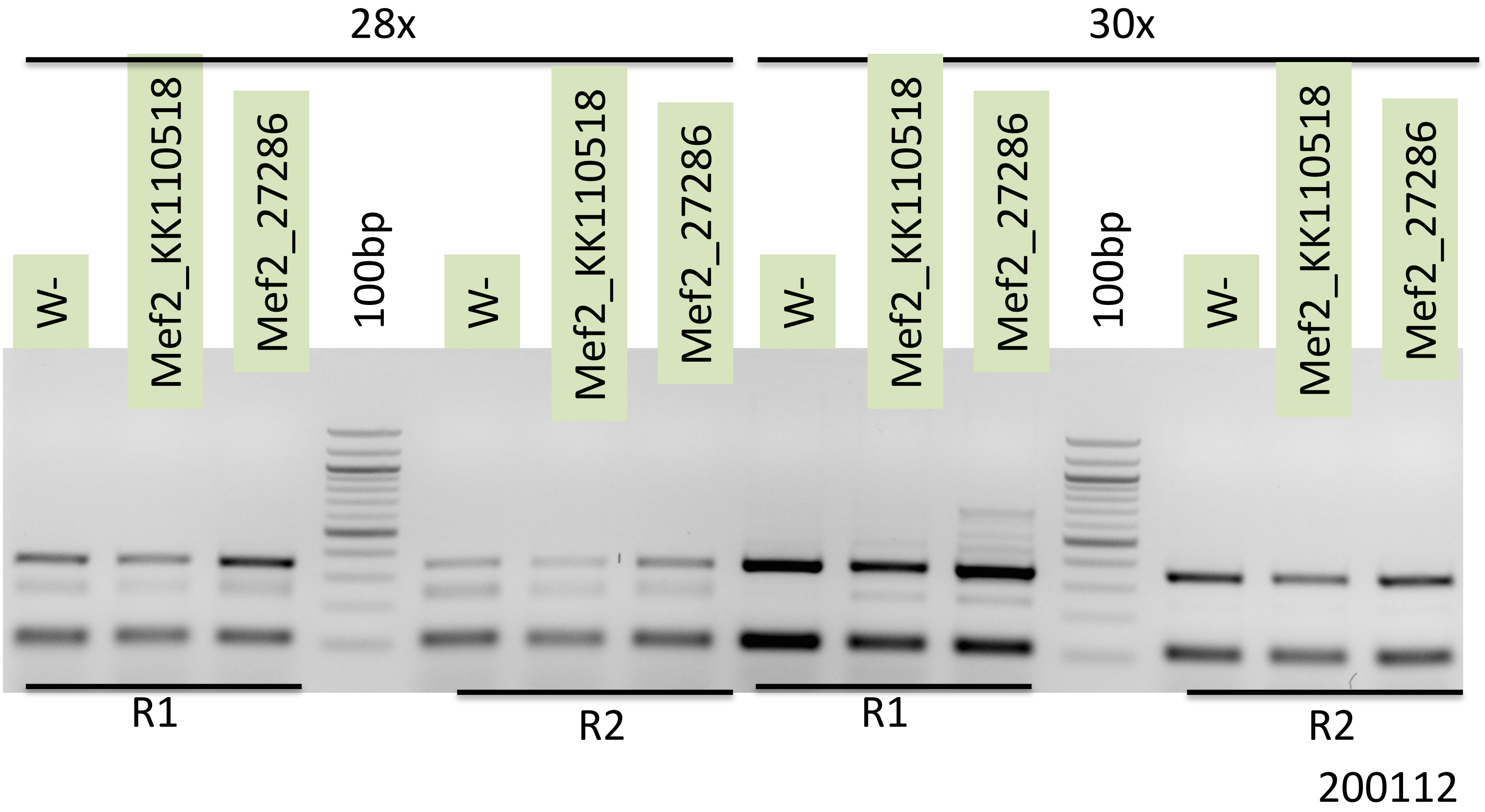

Replicates done from different MMs, run on same PCR block
R1 28x and 30x => coming from a same PCR tube reaction (same for R2)

Tested in IFM tissues

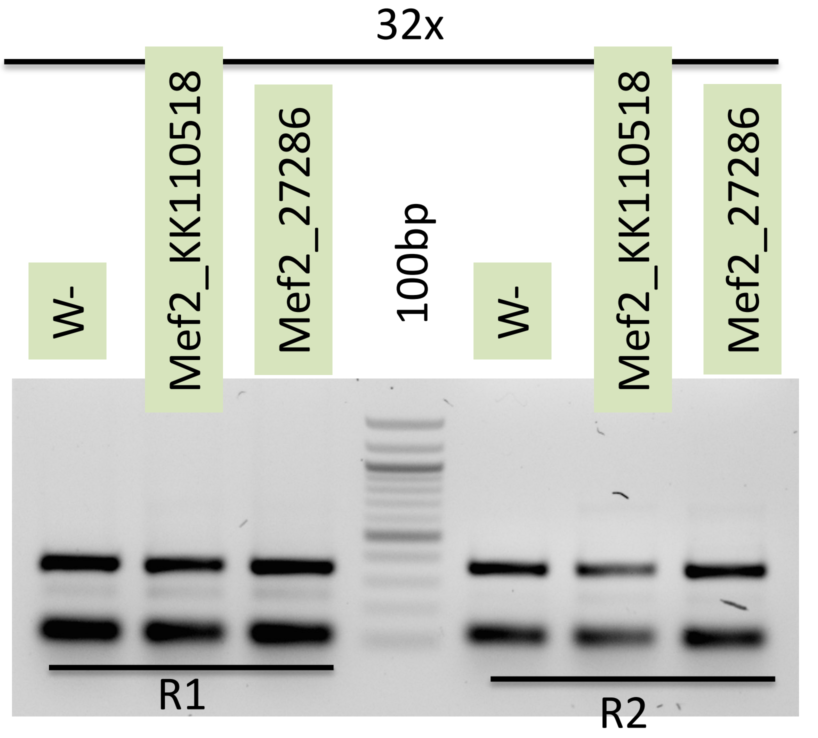

Gel: 200113_//_32x_R1_R2

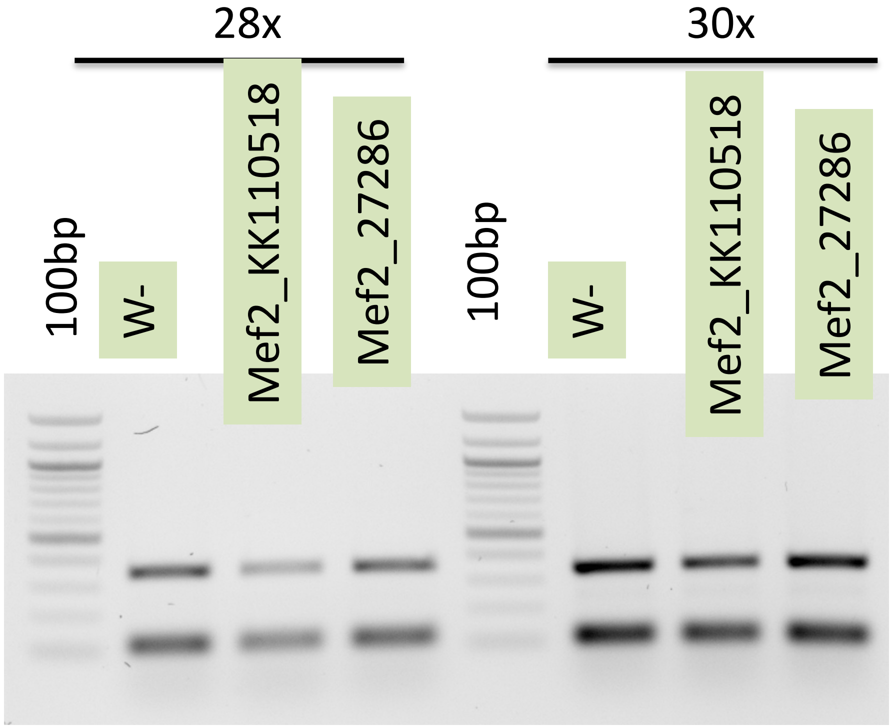

Gel: 210113_//_28x_30x

Tested in IFM tissues

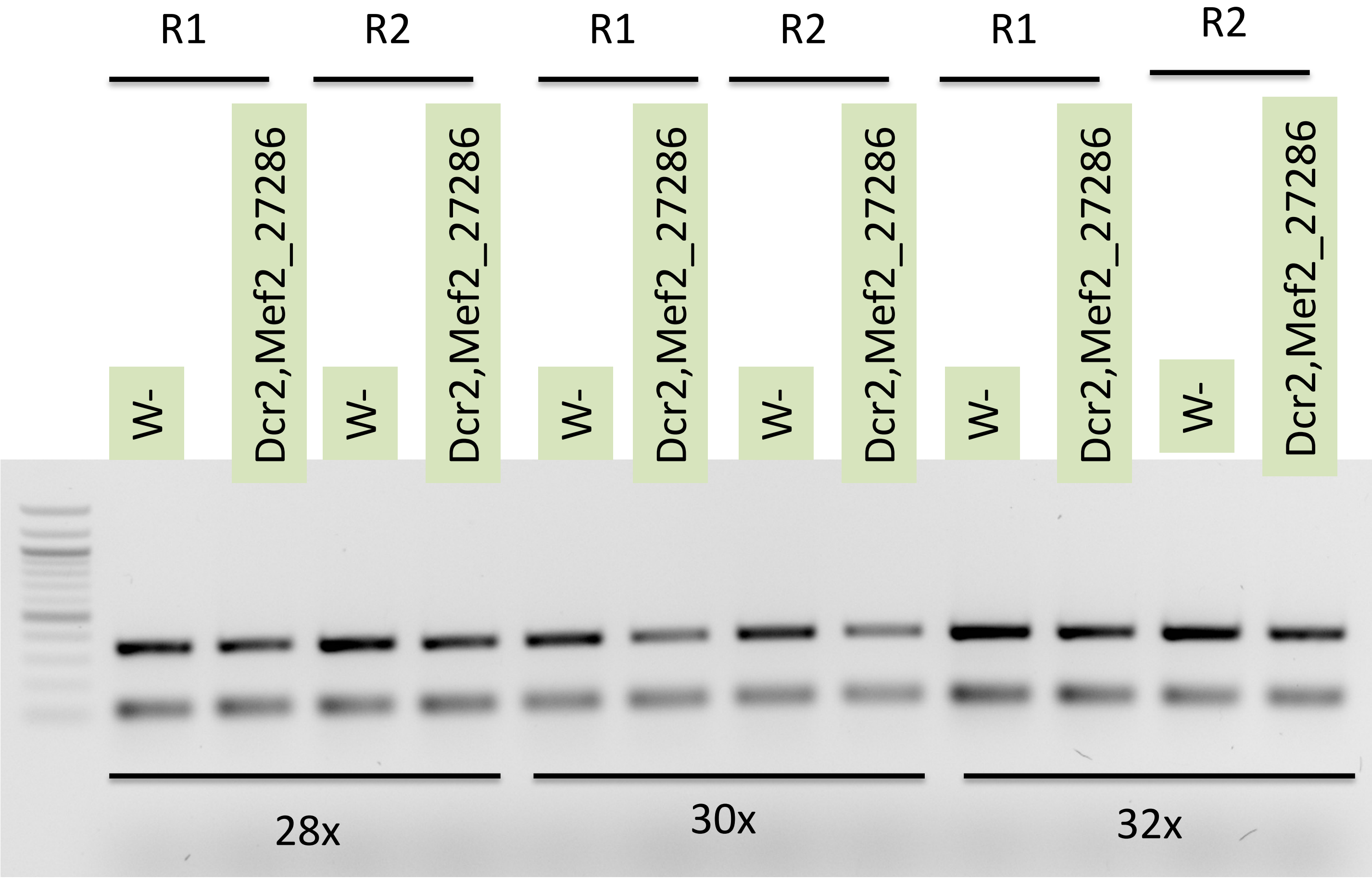

Gel: 210212_fox in dcr2Mef2265_R1_R2

Tested in IFM tissues

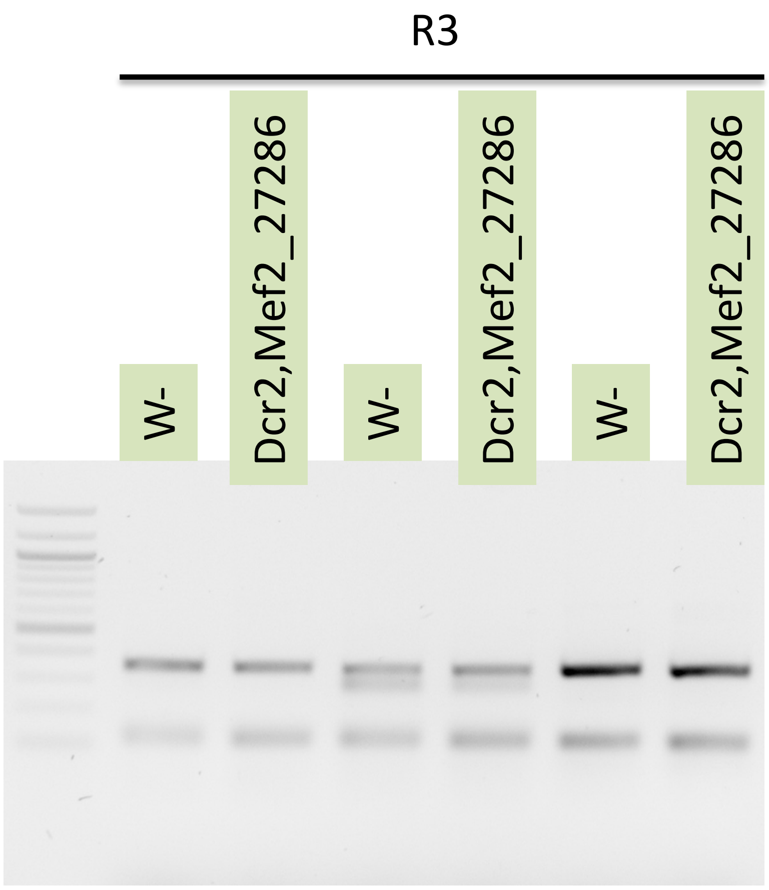

Gel: 210212_2
