# Supplementary material for: Rbfox1 is required for myofibril development and maintaining fiber type–specific isoform expression in Drosophila muscles
Source: Life Sci Alliance. 2022 Jan 7;5(4):e202101342. doi: 10.26508/lsa.202101342 (PMC8742874; doi:10.26508/lsa.202101342)
Supplement: Supplementary file 9 [file LSA-2021-01342_SdataFS4.pdf]

Raw data used to generate plots

Figure panel

	TnI in Rbfox1 KD				TnI in Rbfox1 OE		lef2 > KK11	lef2 > KK110518
	IFM		TDT		IFM		0.814741	0.938502
	control	ef2 > Rbfox1^{R}	control	ef2 > Rbfox1^{R}	control	lef2 > Rbfox	0.69539	0.724432
**S4A**	1	0.01200518	1	1.060119	1	6.229417	0.514623	0.906992
	1	2.394308	1	6.298547	1	1.323514	0.81419	
	1	5.170797	1	1.036219	1	3.078235	0.589012	

	Act88F in Rbfox1 KD				Act88F in Rbfox1 OE	
	IFM		TDT		IFM	
	control	ef2 > Rbfox1^{R}	control	ef2 > Rbfox1^{R}	control	lef2 > Rbfox1
**S4B**	1	0.7410838	1	1.043933	1	0.1371753
	1	1.849098	1	1.651025	1	0.2340307
	1	0.646982	1	0.5610681	1	0.3716996

	IFM				TDT		
	w-	KK101518	27286	Dcr,27286	TDTw-	DTKK10151	TDT27286
**S4D**	1	0.912	1.2224	1.1547	1	0.3741	0.4657
	1	0.6853	0.9212	0.8331	1	0.7186	0.6969
					1	0.6248	0.6894

		Bru1	both	Rbfox1
**S4K**	SPs	0.515625	0.453125	0.03125
	musclepher	0.568131	0.34009	0.091779
	all	0.585725	0.311094	0.103181

Original gels RT-PCR

Exd and Act88F levels in Fox1KD

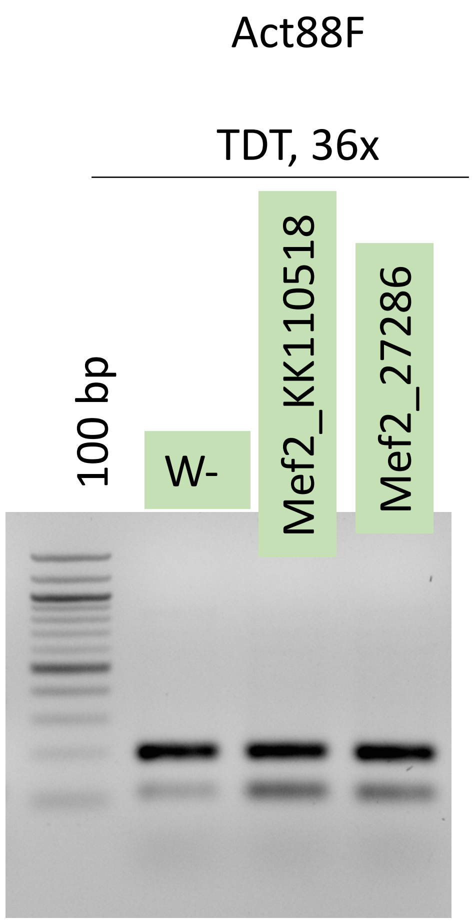
Act88F
TDT, 36x
100 bp
W-
Mef2_KK110518
Mef2_27286

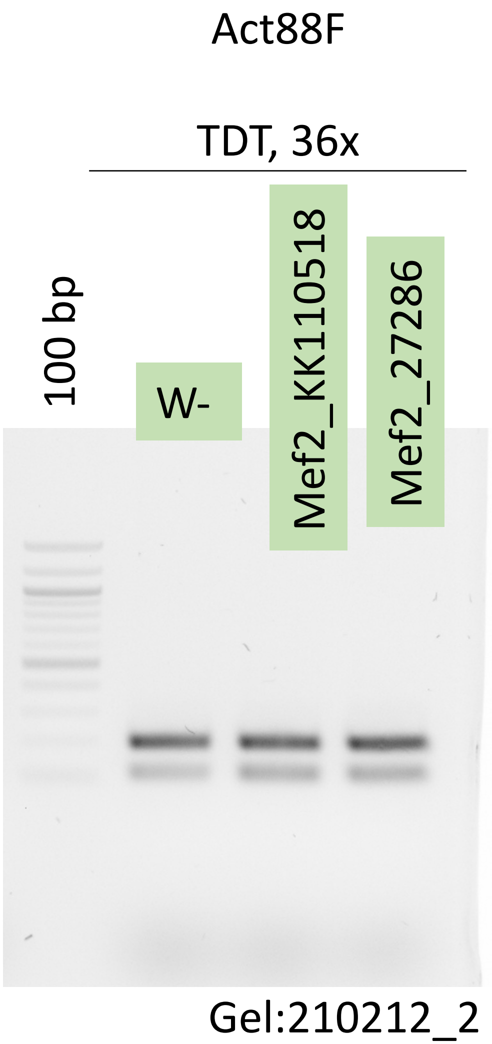
Act88F
TDT, 36x
100 bp
W-
Mef2_KK110518
Mef2_27286
Gel:210212_2

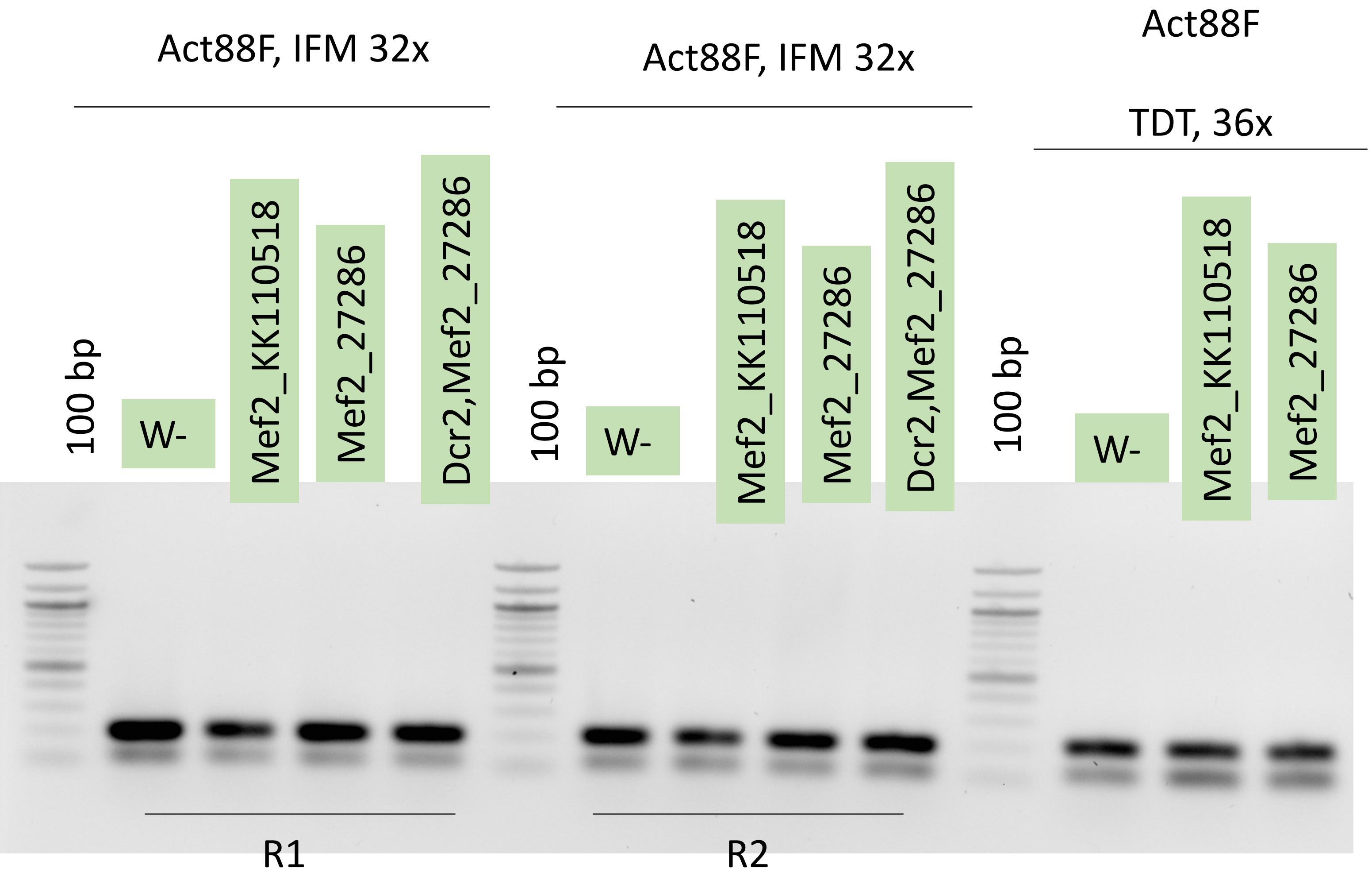
Act88F, IFM 32x
Act88F, IFM 32x
Act88F
TDT, 36x
100 bp
W-
Mef2_KK110518
Mef2_27286
Dcr2,Mef2_27286
100 bp
W-
Mef2_KK110518
Mef2_27286
Dcr2,Mef2_27286
100 bp
W-
Mef2_KK110518
Mef2_27286
R1
R2

Gel: 210216_1
