# Supplementary material for: Rbfox1 is required for myofibril development and maintaining fiber type–specific isoform expression in Drosophila muscles
Source: Life Sci Alliance. 2022 Jan 7;5(4):e202101342. doi: 10.26508/lsa.202101342 (PMC8742874; doi:10.26508/lsa.202101342)
Supplement: Supplementary file 11 [file LSA-2021-01342_SdataF5.pdf]

Raw data used to generate plots

Figure panel

	IFMw-	10.36392	3.210597	10.91125	8.61027	6.569576			2.78734	5.808399	4.735989	5.034785	6.184611			
	IFMMef2w-	6.951901	6.467338	5.029998	5.367419	4.808591			6.16	5.275659	4.020292	8.066185	13.28779	8.89396	5.399252	
	IFM27286	2.821207	5.36273	2.662916	3.122319	8.226132	4.492958		5.791357	6.031401	7.120792	5.049556	5.982743	7.30055		
	IFMKK1015	1.820185	1.981659	1.834678	1.967447	1.730245	1.974214	2.105633	2.060042	2.251457	2.148241	2.307773	2.528652	2.338864	2.159853	
	IFMBru1M2	1.99823	1.462262	2.177849	1.39083	1.894327			1.773957	1.420951	1.878129	1.762897	2.20126	1.798287		
	TDTw-	1.817757	2.268141	2.692818	5.055706	5.973553	4.890503		1.751168	2.331473	1.978609	2.783288	4.116803	3.039422		
	TDTMef2w-	2.472457	3.638106	3.636685	3.865079	2.611493	3.890647	3.581506	2.634124	2.99622	2.521303	2.341858	2.678941	2.879735		
**5K**	TDT27286	3.911771	2.472036	2.136623	1.903909	1.98846	1.950355		2.14389	2.403035	1.943251	2.013257	2.042092	2.585028	3.963765	
	TDTKK101	2.203465	2.158316	1.992171	1.801136	2.028701			2.338349	2.388223	2.042831	2.242424	2.264031			
	TDTBru1M2	2.823685	1.410699	2.197137	2.541173	2.069032	1.928393		2.576689	2.467013	2.680425	2.573171	2.031467	2.358764		
	Abd-Mw-	2.547874	2.099406	2.472211	1.410771	2.018519	2.28961	1.916458								
	Abd-MMef2	1.947982	1.69417	2.516351	3.477734	2.300376	1.941707									
	Abd-M2728	1.906394	1.777027	2.033075	2.592369	2.041378	2.443322	2.120833	2.534599							
	Abd-MKK10	1.230864	2.141324	1.901965	1.671926	2.082321	2.118878		2.513066	1.8491	1.708715	3.781087	2.132229	1.480465		
	Abd-MBru1	2.546609	1.525483	1.980213	2.554244	1.888986	2.265534	1.938653	1.834678	2.36736	1.881052		2.822726	2.817642	2.122977	1.89587

		WT Bru1-RB				KK101518 Bru1-RB				KK101518 Bru1-RA			
**5M**	IFM	1	1	1	1	0.004519	0.007509	0.442007	0.14699	0.711414	1.000774	0.703601	1.053176
	TDT	1	1	1	1	0.058091	0.006109	0.459687	0.182957	1.014842	1.018163	1.116211	1.076011
	Abd	1	1	1	1	0.018974	0.138255	0.05353	0.596302	0.625121	1.034773	0.584167	0.741341
		27286 Bru1 -RB		27286 Bru1-RA		WT Bru1-RA							
	IFM	0.339164	0.396049	1.247711	1.987338	1	1	1					
	TDT	1.456458	3.047977	1.829812	2.532121	1	1	1					

	Sample	% bru1 Rblong isoform													
	IFMw-	0.539667	0.75544	0.496779	0.534486	0.43774	0.478694	0.441523	0.630331	0.595474	0.315433	0.309346			
	IFM27286	0.578062	0.725943												
	IFMDcr2,27	0.789106	0.846248	0.752471	0.737236	0.966365	0.964627	0.891827	0.906615						
**5P**	IFMKK1015	0.724714	0.796146	0.921929	0.937258	0.952342	0.960636	0.898788	0.889759						
	TDTw-	0.290849	0.15748	0.682019	0.693197	0.759347	0.100656	0.050108	0.070335	0.061716	0.094559	0.076678	0.053247	0.142088	0.11813
	TDT27286	0.253948	0.055256												
	TDTDcr2,27286		0.174477	0.108492	0.236246	0.291445									
	TDTKK101	0.267364	0.370082	0.465972	0.247465	0.087665			0.27504	0.334619					
		% bru1 RB short isoform													
	IFMw-	0.460333	0.24456	0.503221	0.465514	0.56226	0.521306	0.558477	0.369669	0.404526	0.684567	0.690654			
	IFM27286	0.421938	0.274057												
	IFMDcr2,27	0.210894	0.153752	0.247529	0.262764	0.033635	0.035373	0.108173	0.093385						
	IFMKK1015	0.275286	0.203854	0.078071	0.062742	0.047658	0.039364	0.101212	0.110241						
	TDTw-	0.709151	0.84252	0.317981	0.306803	0.240653	0.899344	0.949892	0.929665	0.938284	0.905441	0.923322	0.946753	0.857912	0.88187
	TDT27286	0.746052	0.944744												
	TDTDcr2,27286		0.825523	0.891508	0.763754	0.708555									
	TDTKK101	0.732636	0.629918	0.534028	0.752535	0.912335			0.72496	0.665381					

	bru1 mRNA common primers (14 + 17)								
	IFMw-	IFM27286	FMDcr2728	FMKK11051	Mef2>Rbfox	TDTw-	TDT27286	DTDcr2728	DTKK110518

5N,O	1	1.349872	6.606037	6.409671	0.011898	1	0.519242	1.082129	1.281597
	1	1.535095	4.444666	6.452661	0.028392	1	0.350666	0.935739	0.997305
	1	1.696796	2.293325	2.451396	0.022538	1	0.60247	1.070328	1.09536
	1		3.003005	4.062621		1	0.218813		
	1		1.747264	2.509926			0.49034		
							0.39629		
	bru1 mRNA isoform RB primers (5 + 8)								
		IFMw-	IFM27286	FMDcr2728	FMKK11051	TDTw-	TDT27286	DTDcr2728	DTKK110518
		1	1.20779	1.703698	0.574241	1	0.289302	2.219745	0.477784
		1	1.418784	1.855733	0.756938	1	0.264105	1.298527	0.575979
		1		1.466173	2.883405	1		1.587855	0.88503
		1		1.464049	2.709429	1		3.117991	1.620894
		1		3.971184	5.427456	1			1.124197
		1		3.14773	4.267931				2.619484
									3.897268

	M2IFM	M2TDT	M2Abd	UH3RAIFM	79BRATDT	79BRAAbd	BruIR-IFM	BruIR-TDT	controlIFM	controlTDT	controlAbdM	Mef2>Bru1
5S	1.096923	0.925559	1.09417	1.139421	0.599809	1.127029	0.588841	0.311454	1	1	1	0.204878
	1.23915	1.092261	1.198249	1.229551	0.853995	1.284146	0.467999	0.489551	1	1	1	0.096248
	1.491719	1.125833	1.1372	1.252351	0.8098	1.165227	0.433881	0.371471	1	1	1	0.56993

		Std. Norm Counts	
		Rbfox1	bru1
5Q	Myo	-0.411552	-1.421987
	16h	1.843554	-0.910896
	24h	-0.34524	-0.055127
	30h	-1.218514	1.459121
	48h	-1.018331	0.889715
	72h	0.220905	0.706095
	90h	0.858597	0.168333
	1d	0.070581	-0.835254

	WTIFM	tdt	leg	salm	bruIR	M3	bru1IR30h	bru1IR72h
5R	0	0.882896	-0.574277	0.07561	-0.934324	-0.358605	-0.376448	-0.961075

Original RT-PCR gels

Bru1 levels in w- and Dcr2,Mef2G4x27286 IFM

32x cycles all

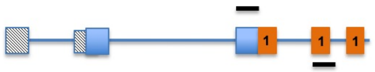

Bru1ex7 F: AGCCTGCCGAATAGTCCC
Bru1 ex8 R: CTTTCAGGGCGGCGTGTC

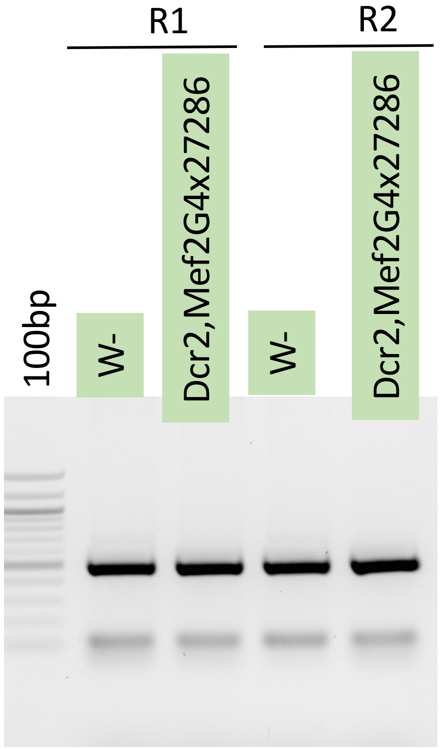

Gel:210212_1

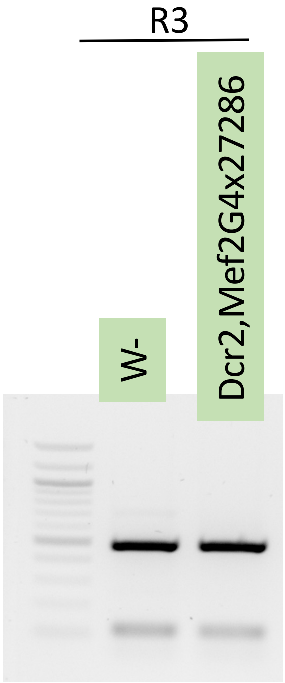

Gel:210212_2

Bru1 C-term

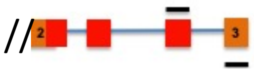

Bru1 ex14 F: CCAGAATCTAGCGGCCATT
Bru1 ex17 R: TTCAATCTGCTTTCCCGC

Bru1 levels in w- and **Mef2G4x27286** IFM

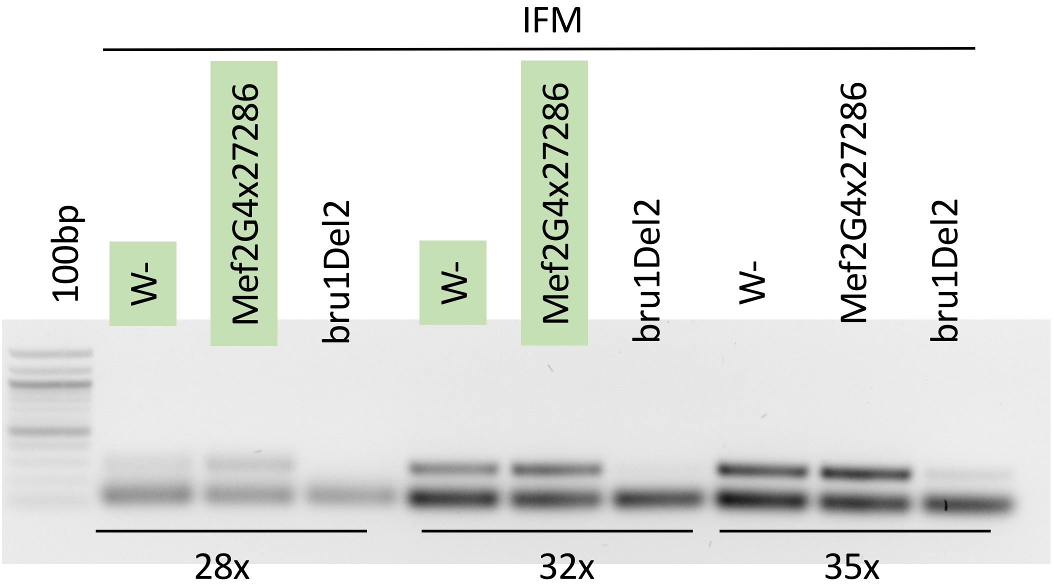

Gel: 200417-a1

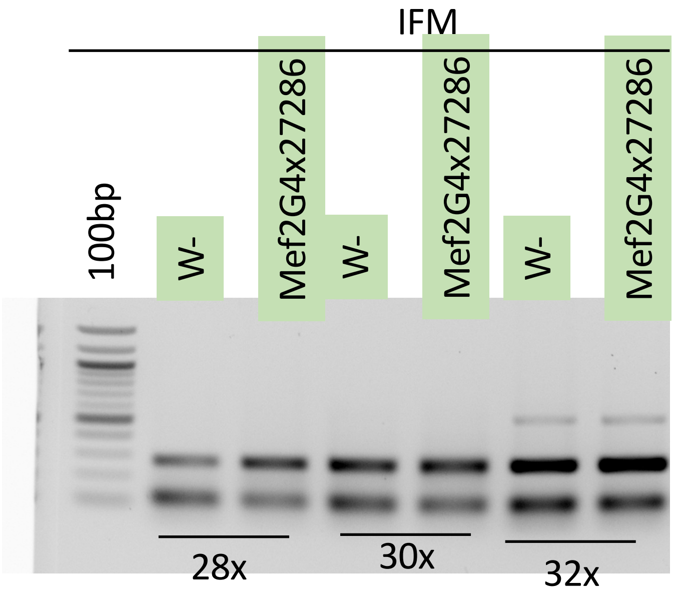

Gel:200529_IFMs_Left_bru1…

Bru1 C-term

Bru1 levels in w- and **Mef2G4x27286** IFM

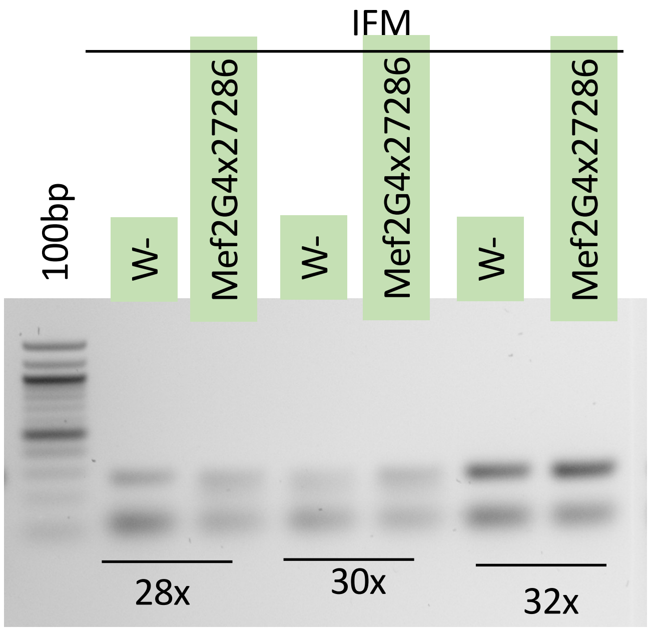

Gel:200605_IFM_///_Right_Bru1

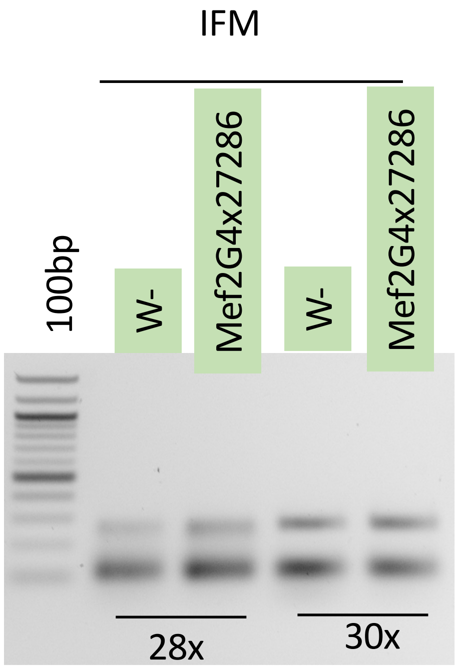

200703_///

Bru1 C-term

Bru1 levels in w- and **Mef2G4x27286** TDT & Abd

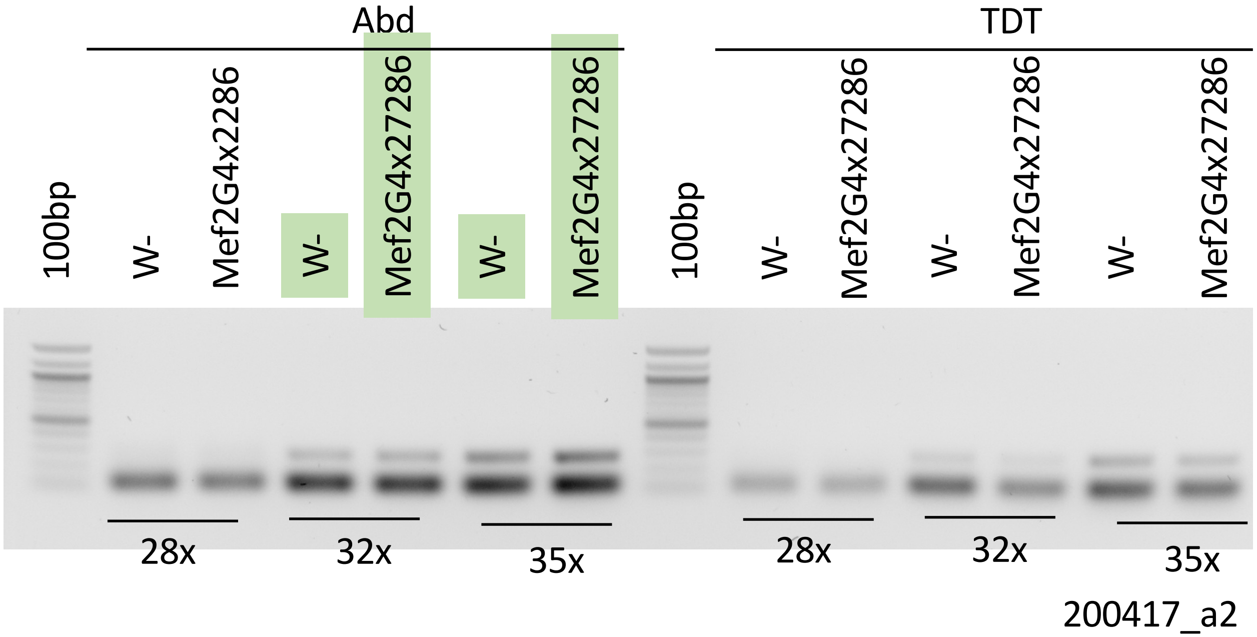

Bru1 C-term

Bru1 levels in w- and **Mef2G4x27286** TDT & Abd

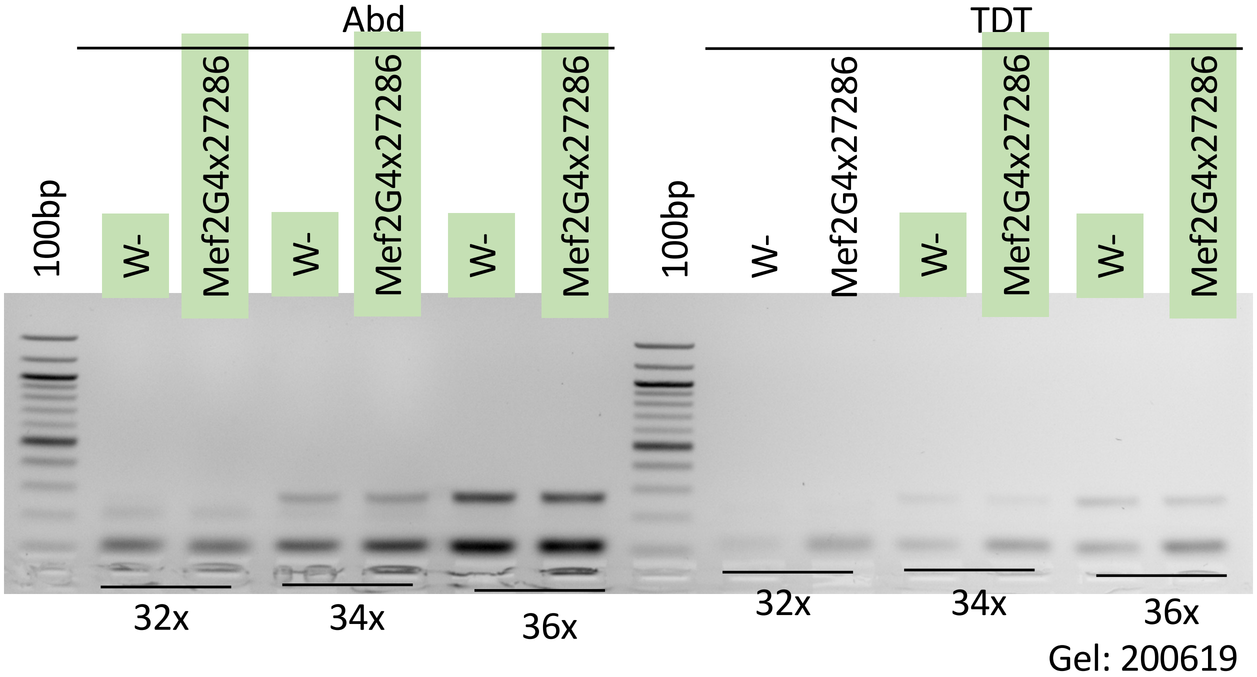

Bru1 C-term

Bru1 levels in w- and **Mef2G4x27286** TDT & Abd

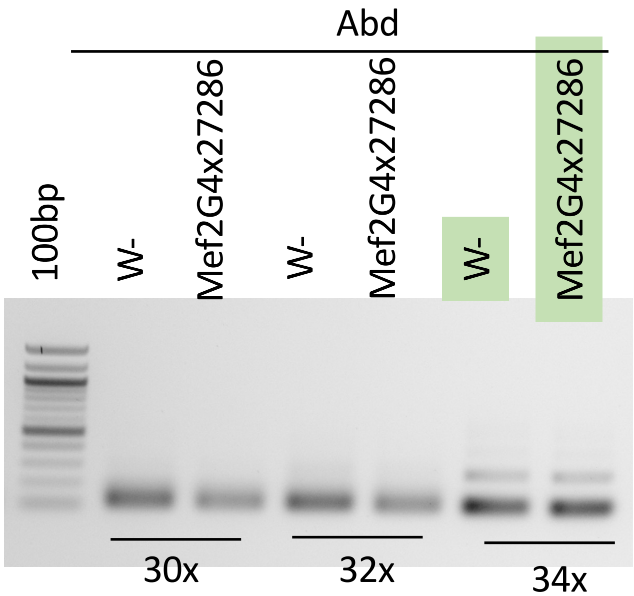

Gel: 200622a

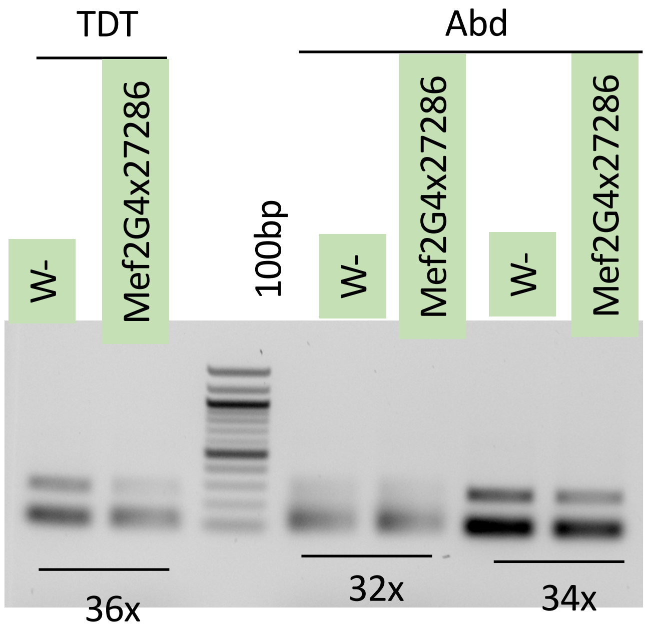

Gel: 200622b

Bru1 C-term

Bru1 levels in w- and **Mef2G4x27286** TDT & Abd

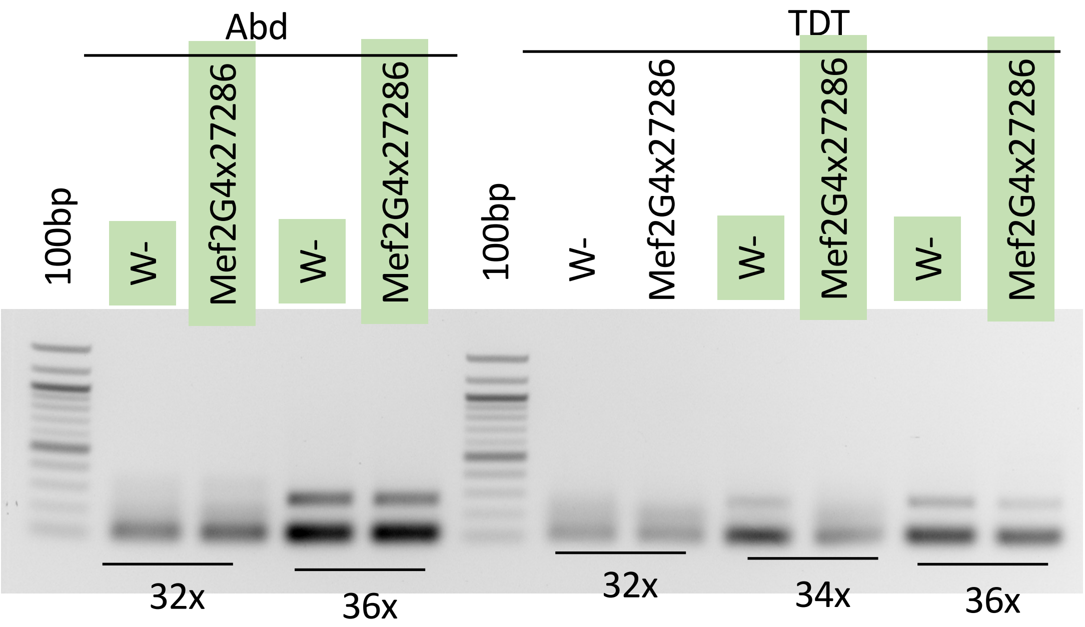

Gel: 200623

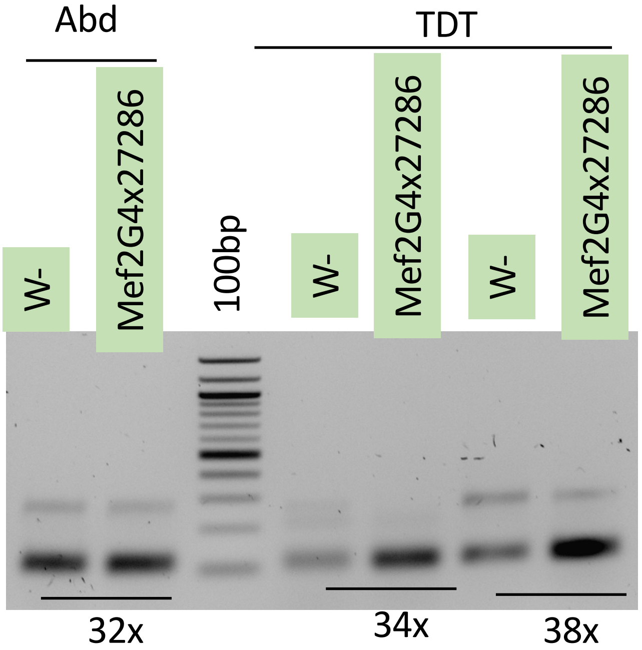

Gel: 200624

Bru1 C-term

Bru1 levels in w- and **Mef2G4x**27286 TDT & Abd

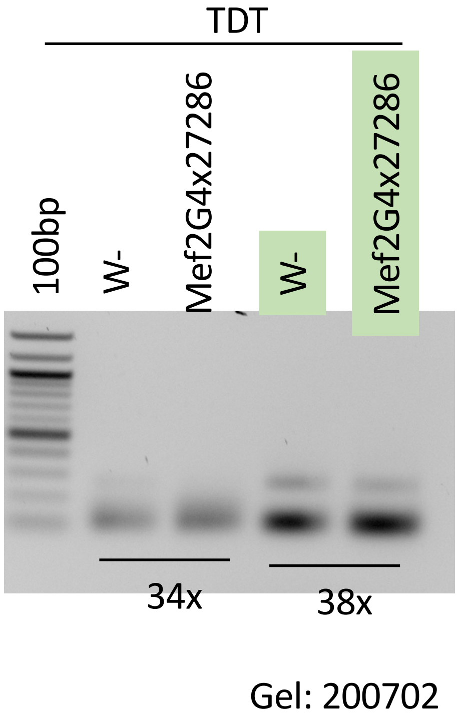

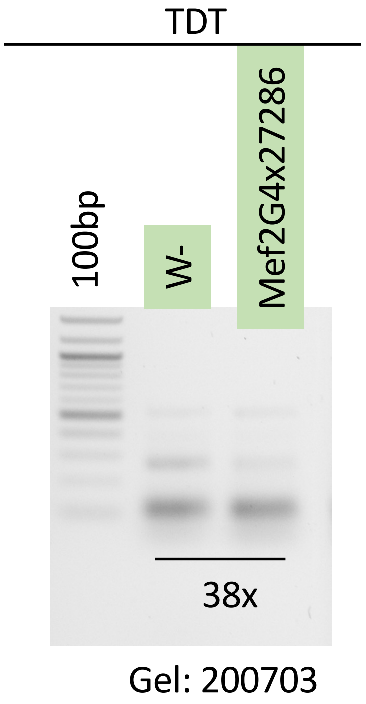

Bru1 C-term

Bru1 levels (C-term) in w- and **Mef2G4xKK110518 & Dcr2,Mef2x**27286 IFM

32x cycles all

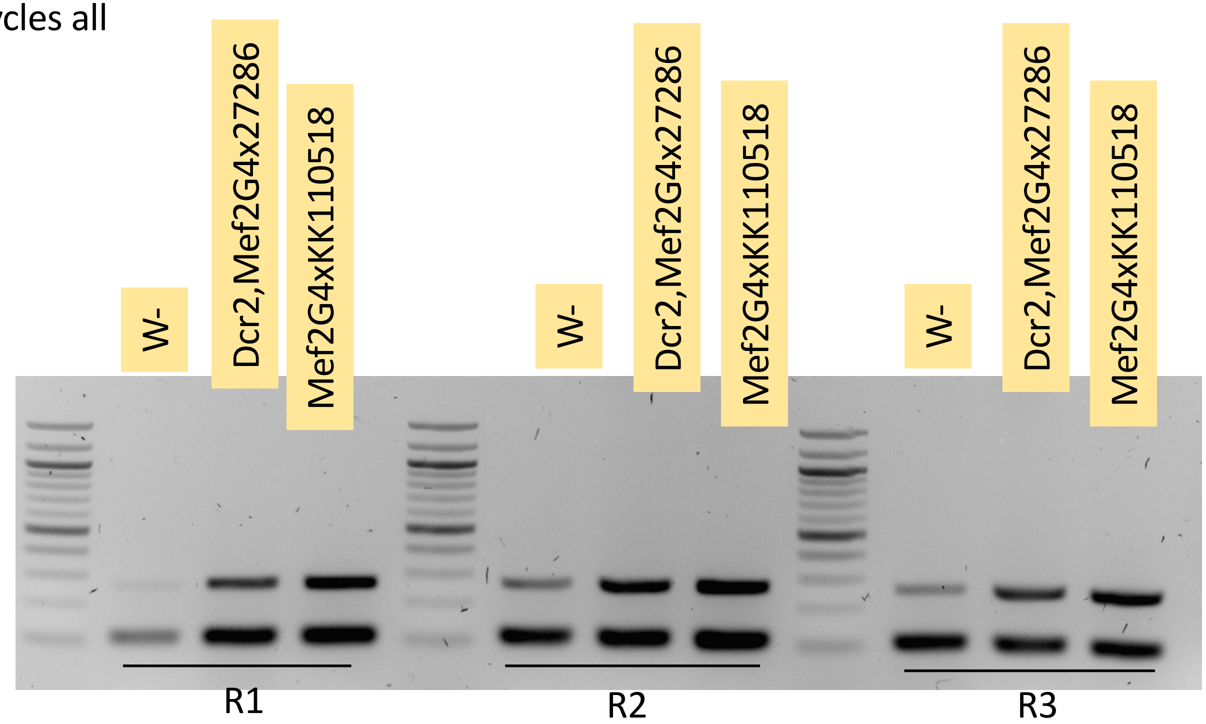

This gel: 211125_rerun_1417_800ms

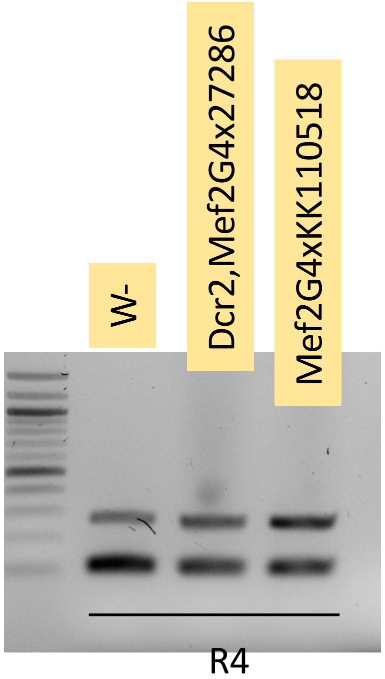

This gel: 211125_rb_900ms

Bru1 levels (C-term) in w- and **Mef2G4xKK110518 & Dcr2,Mef2x**27286 IFM

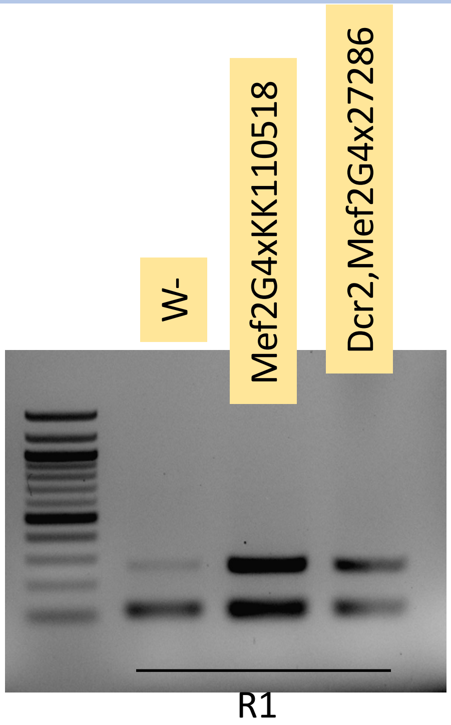

Gel: 2111125_1s

Bru1 RB levels in w- and **Mef2G4xKK110518 and Dcr2,Mef2x27286 TDT**

All 36x cycles

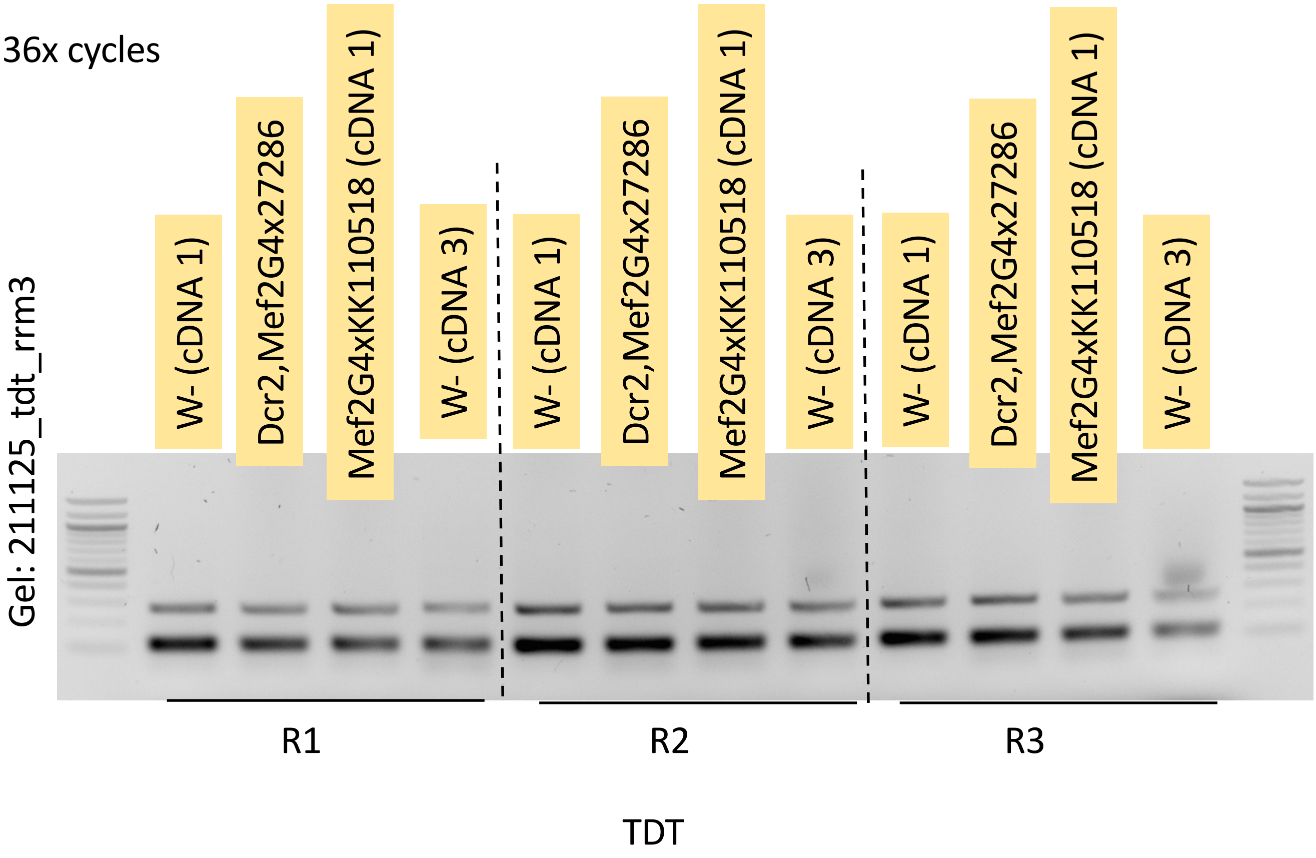

Bru1 N-term

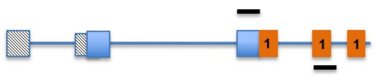

Bru1ex7 F: AGCCTGCCGAATAGTCCC
Bru1 ex8 R: CTTTCAGGGCGGCGTGTC

Bru1 levels in w- and Mef2G4x27286 IFM, TDT and Abd

32x cycles all

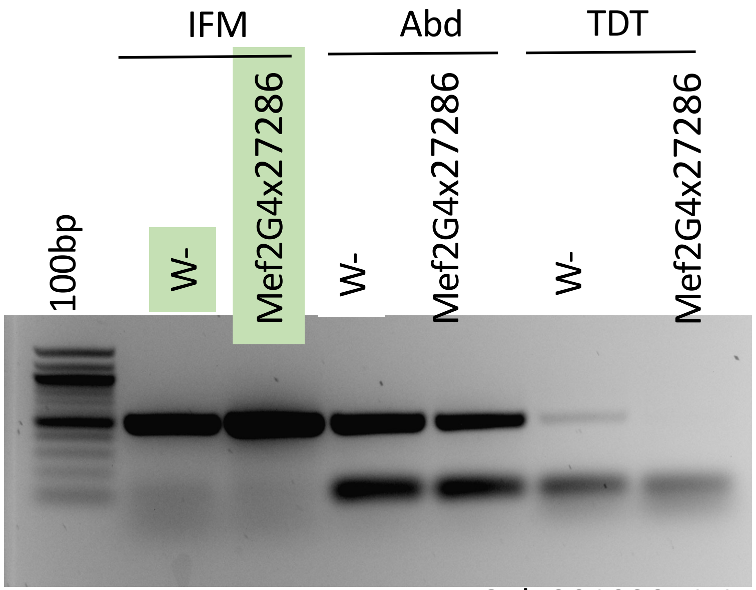

Gel: 201020_1.1

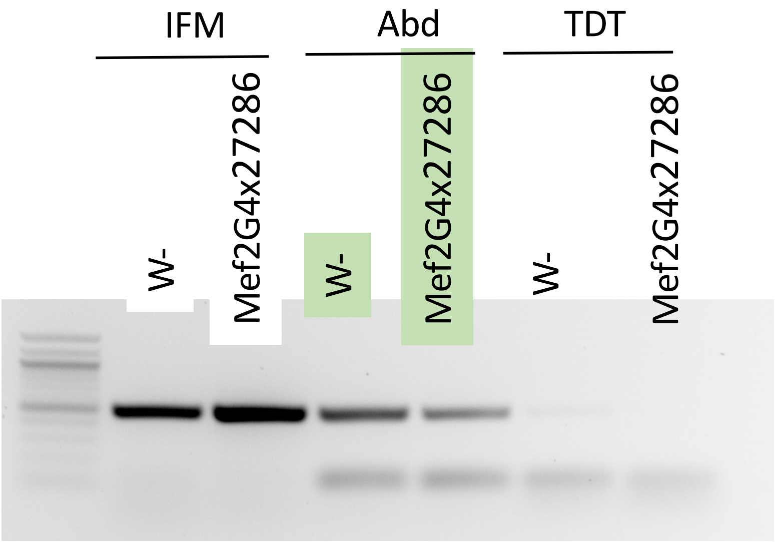

Gel: 201020_1

Note: same gel, different exposures

Bru1 N-term

Bru1 levels in w- and Mef2G4x27286 IFM, TDT and Abd

32x cycles all

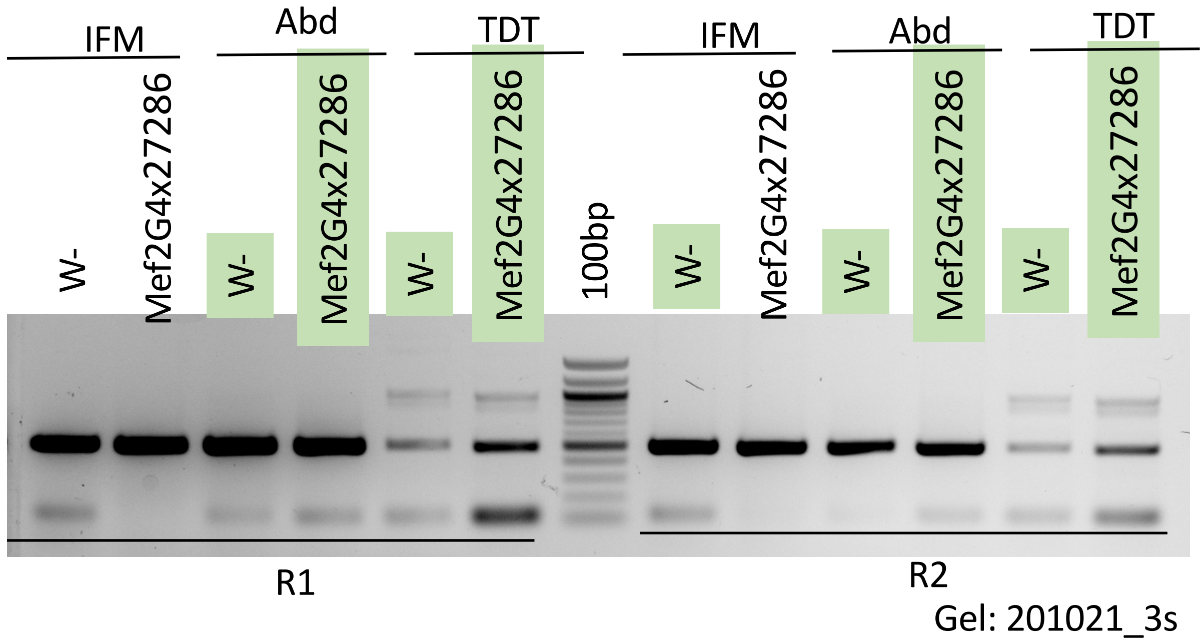

Replicates are form different MMs, but runed on same PCR block/gel

Bru1 N-term

Bru1 levels in w- and Mef2G4x27286 IFM, TDT and Abd

32x cycles all

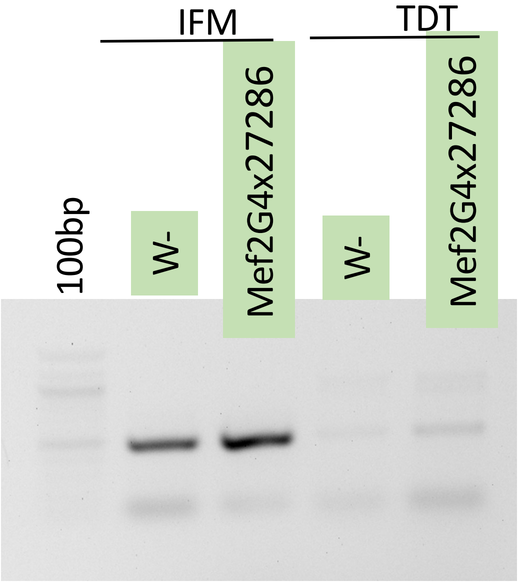

Gel: 201023_auto

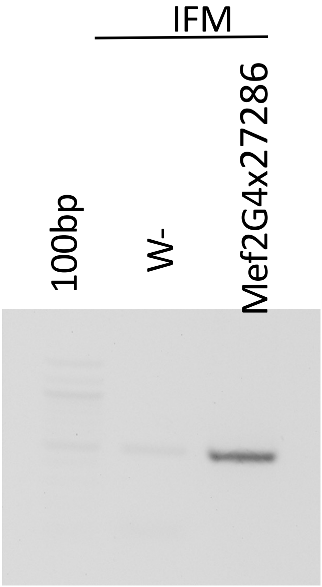

Gel: 201027

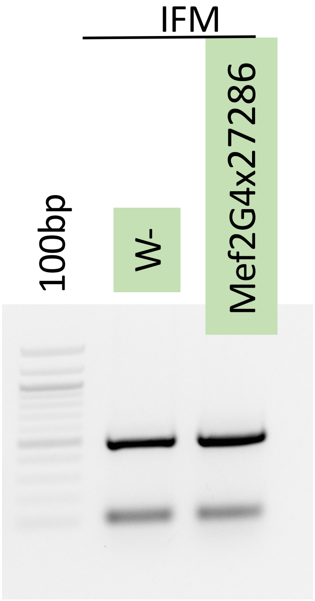

Gel: 201229

Bru1 N-term

Bru1 levels in w- and Mef2G4xKK110518 IFM, TDT and Abd

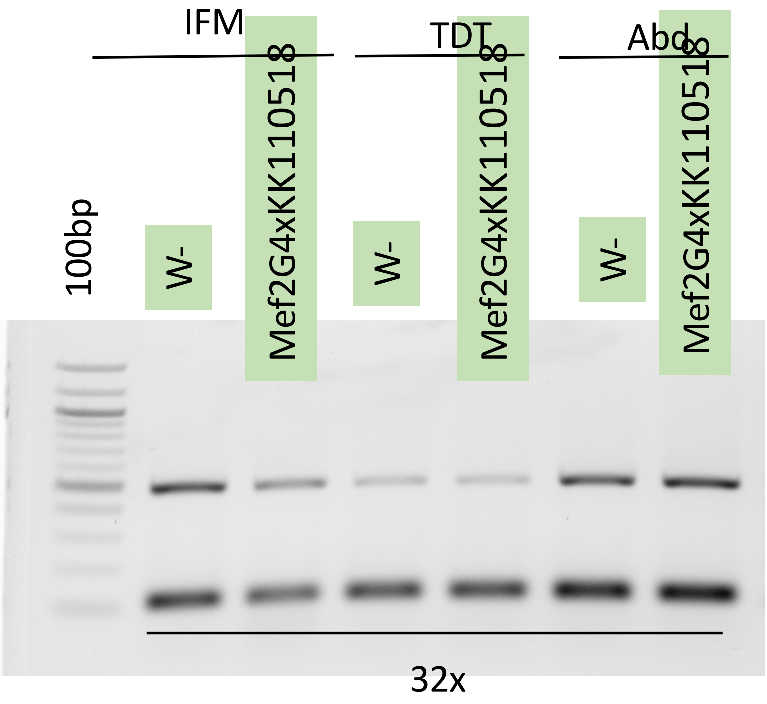

Gel: 201221_Left_Bru1R2_IFM_TDT_Abd

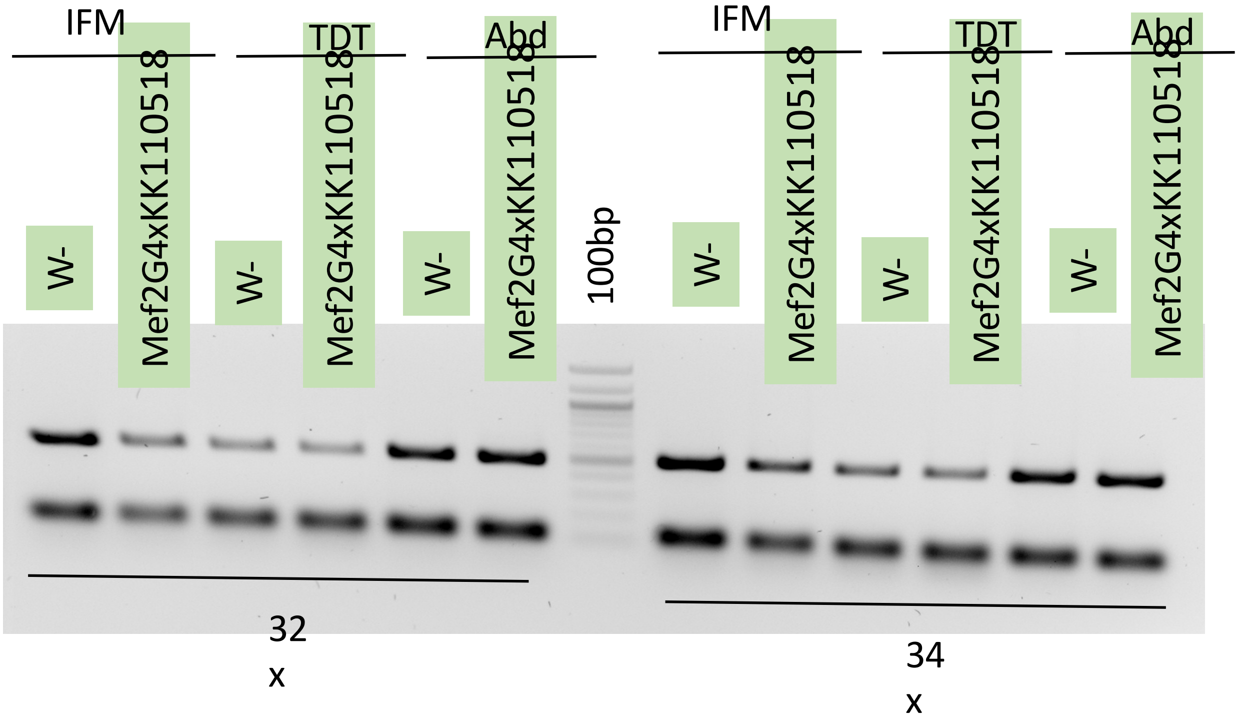

Gel: 201222_Bru1_32X_34X

Bru1 N-term

Bru1 levels in w- and Mef2G4xKK110518 IFM, TDT and Abd

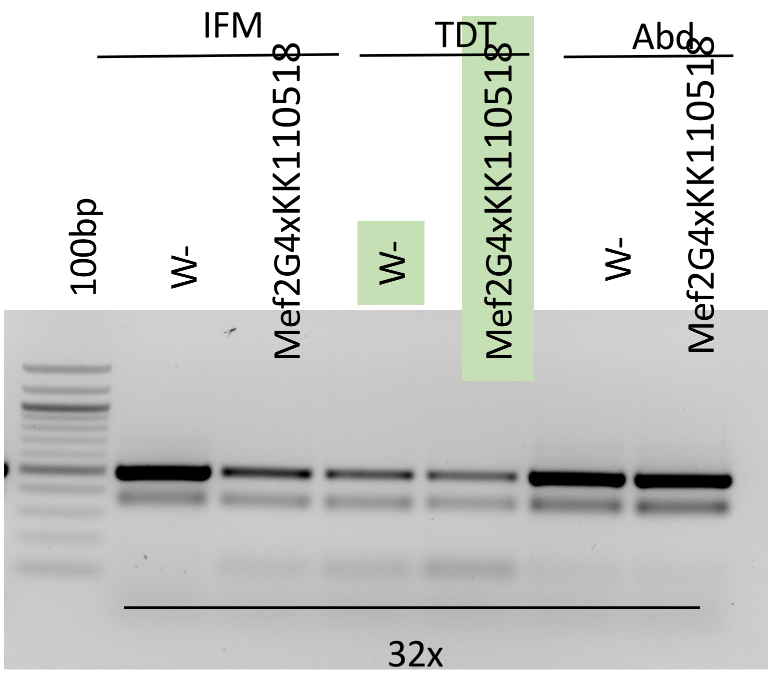

Gel: 201223_Bru1_32x_rep

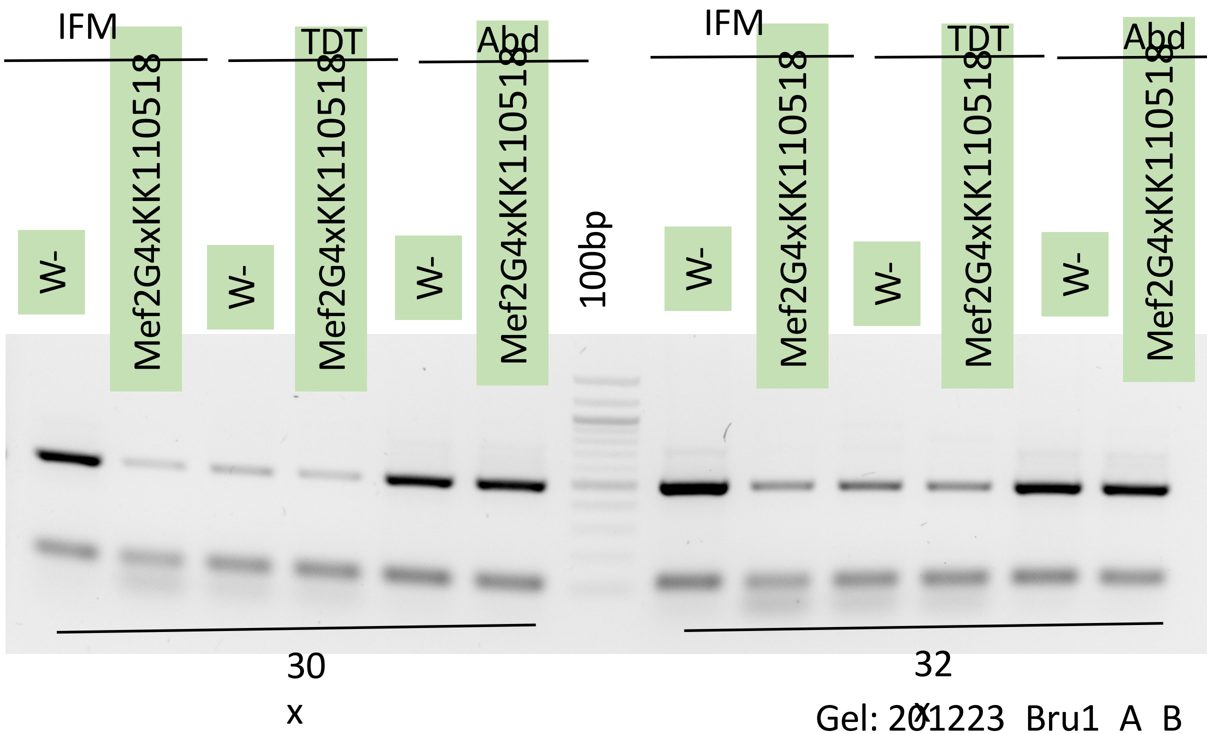

Gel: 201223_Bru1_A_B

Bru1 N-term

Bru1 levels in w- and **Mef2G4x27286** IFM, TDT, Abd with RB/RG primers

Bru1 RB iso

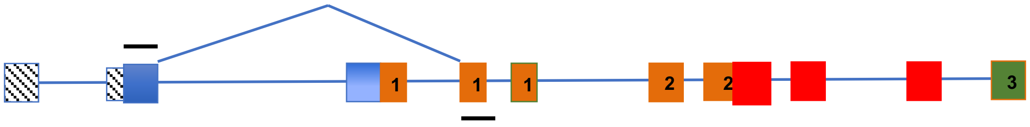

Bru1 long isoform

Bru1 ex5 F: AAC TGG AGT TCG GTC TCG C

Bru1 ex8 R: CTTTCAGGGCGGCGTGTC

All done at 32x cycles

R1

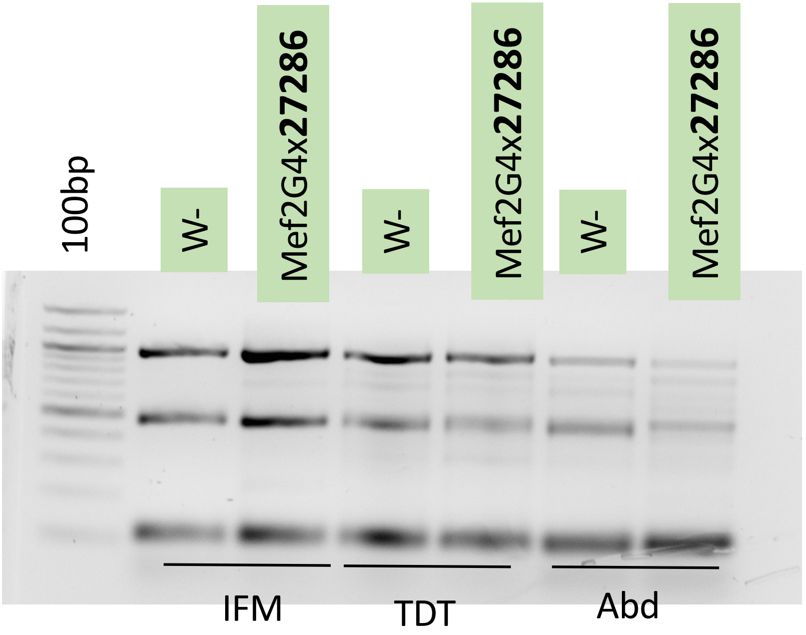

Gel: 200731

R2

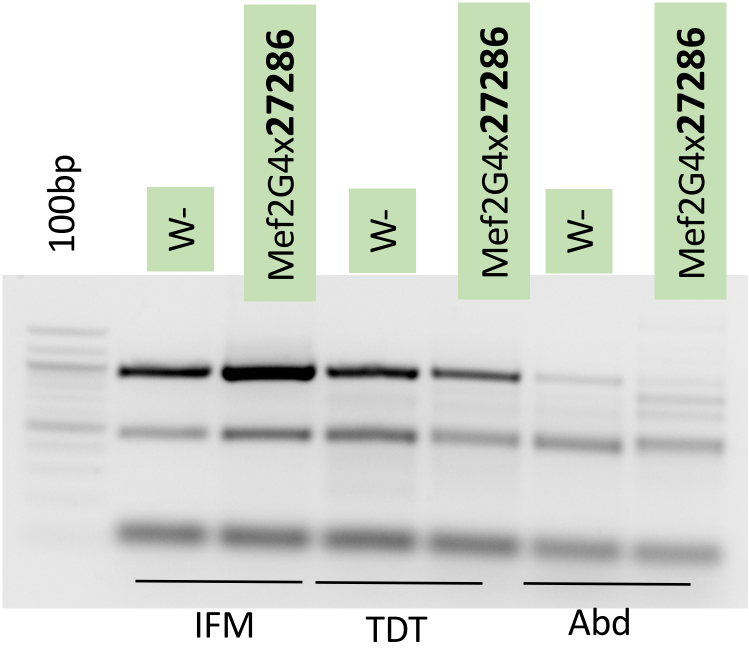

Gel: 200804

Bru1 RB iso

Bru1 RB levels in w- and **Mef2G4xKK110518 & Dcr2,Mef2x27286** IFM

All done at 32x cycles

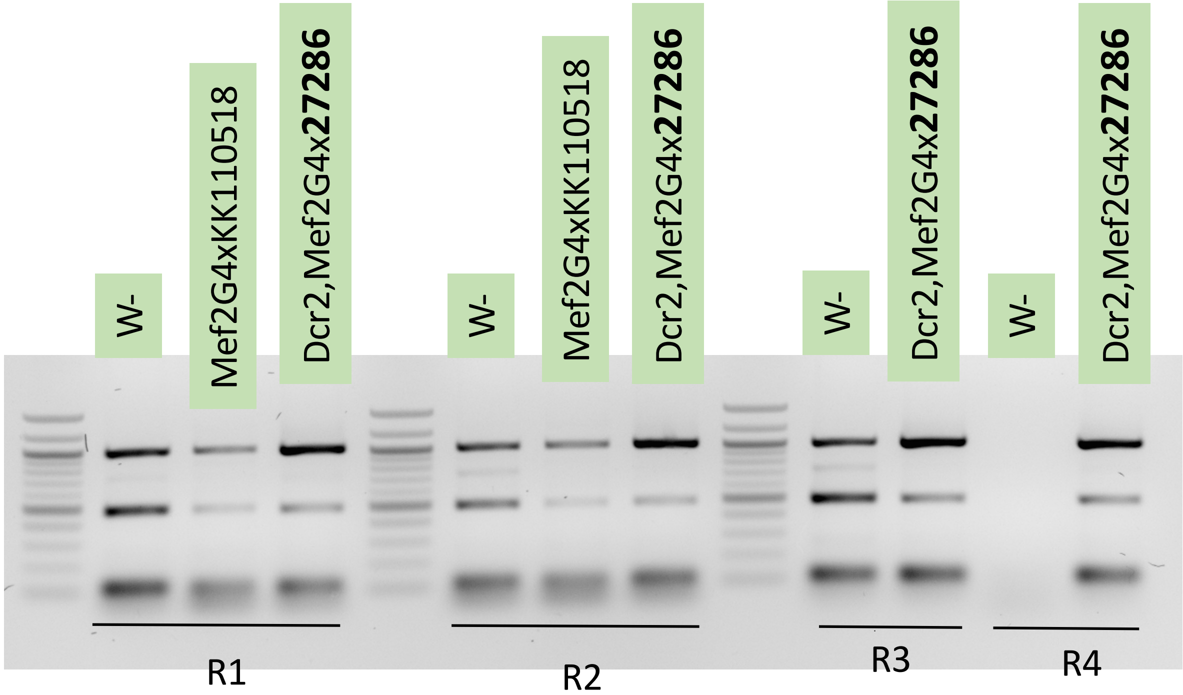

Gel: 210303_1_IFM

Bru1 RB iso

Bru1 RB levels in w- and **Mef2G4xKK110518 TDT and Abd**

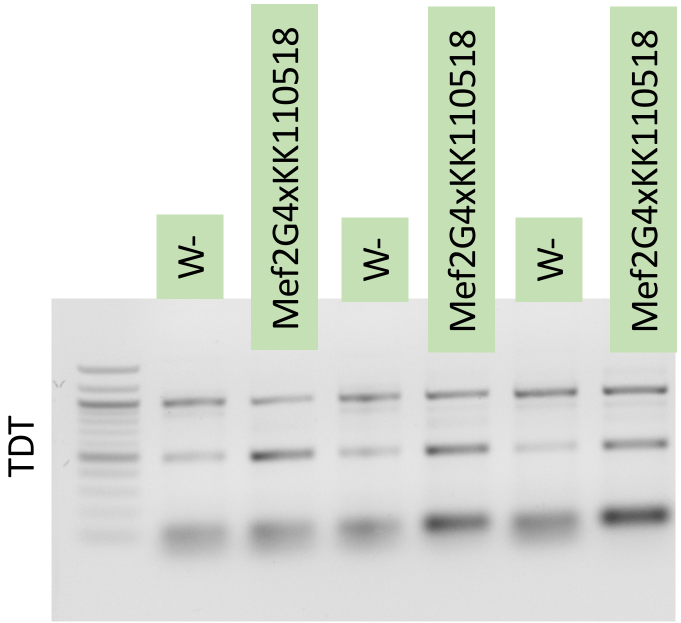

Gel: 210303_2_TDT_Abd

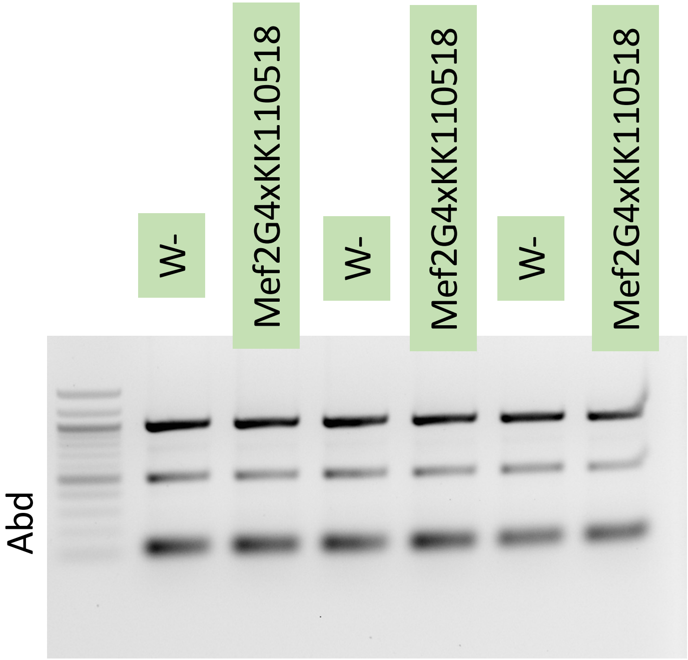

Gel: 210303_2_TDT_Abd

Bru1 RB iso

Bru1 RB levels in w- and **Mef2G4xKK110518 IFM and TDT**

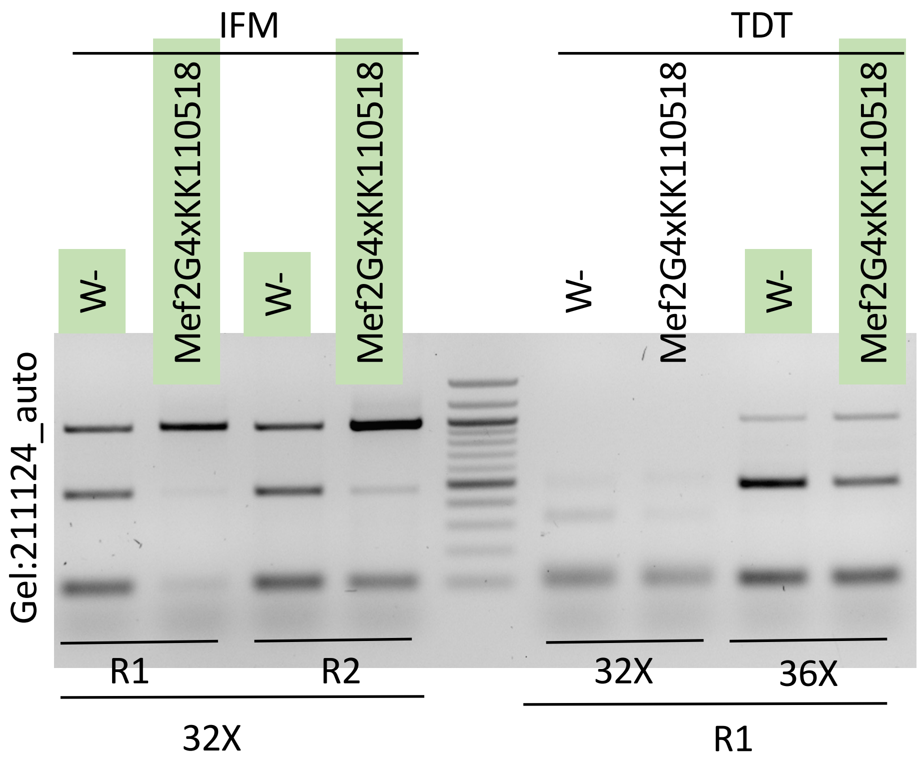

Bru1 RB iso

Bru1 RB levels in w- and **Mef2G4x**KK110518 **and Dcr2,Mef2x27286 IFM**

32x cycles

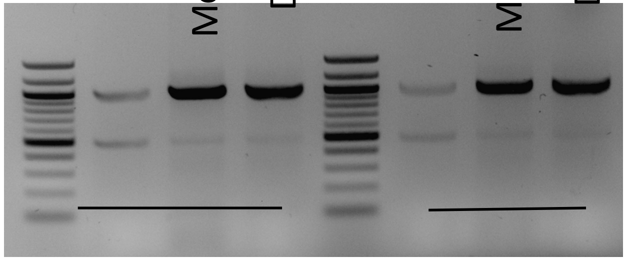

Gel: 2111125_1s
(also 2111125_1.5s)

RP49 didn't work, so not quantifiable, but shows that the trend is similar at KDs

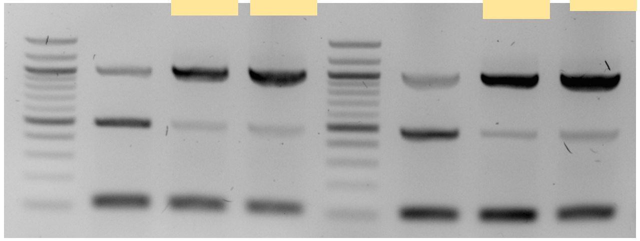

This gel: 211125_rb_900ms
(also 211125_rb_auto)

The loading order is correct, I realized that in left gel I loaded 266 in front, and correct the loading order in the next PCR

Bru1 RB iso

Bru1 levels in w- and **Mef2G4xKK110518 & Dcr2,Mef2x27286** TDT

All 36x cycles

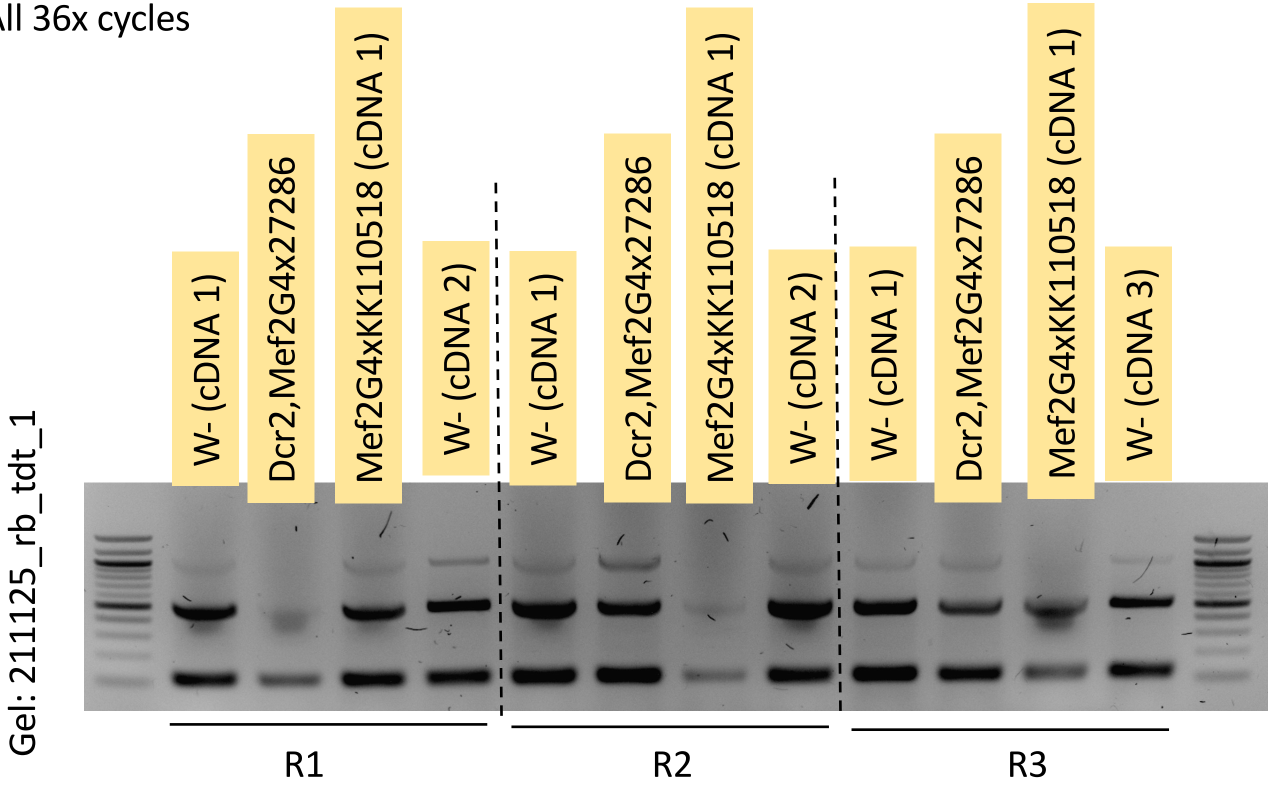

TDT

Bru1 RB iso

Bru1 levels in w- and **Mef2G4xKK110518 & Dcr2,Mef2x27286** TDT

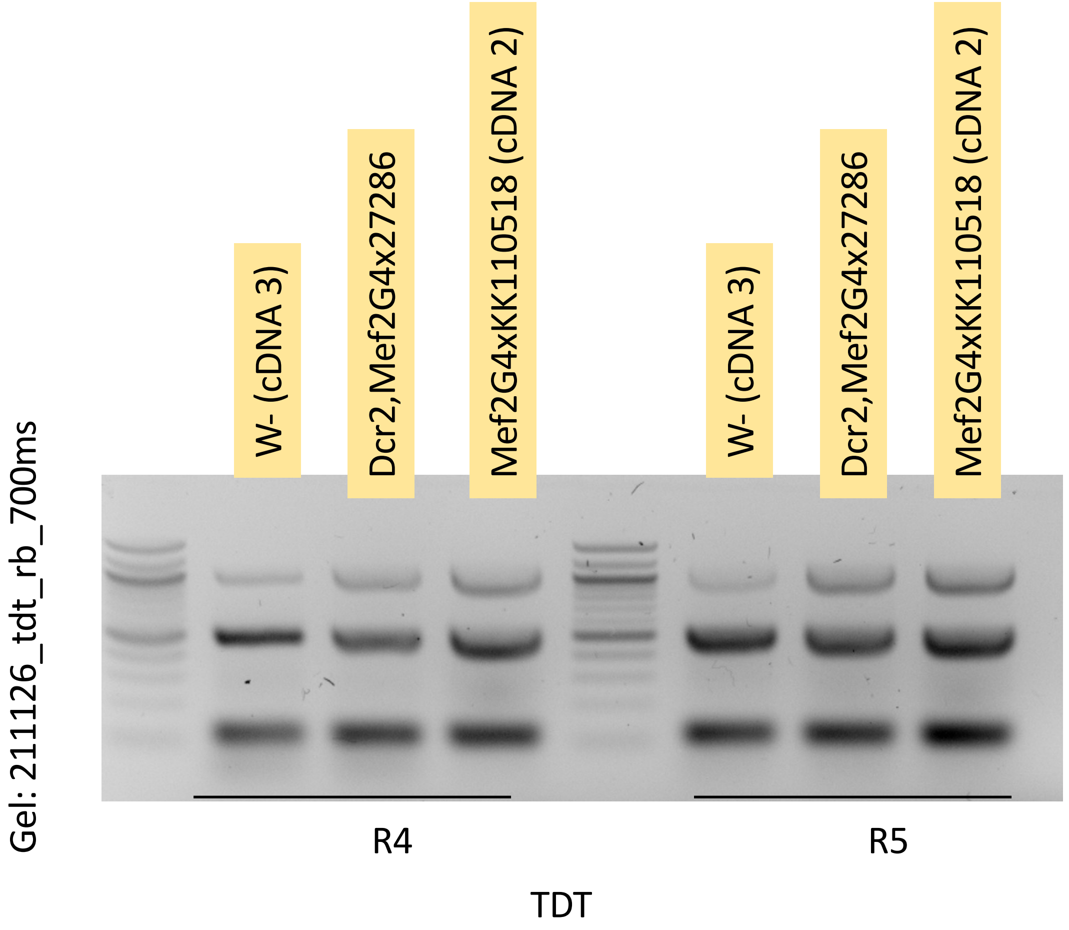

Bru1 RB iso

Rbfox1 levels in bru1Del2 IFMs

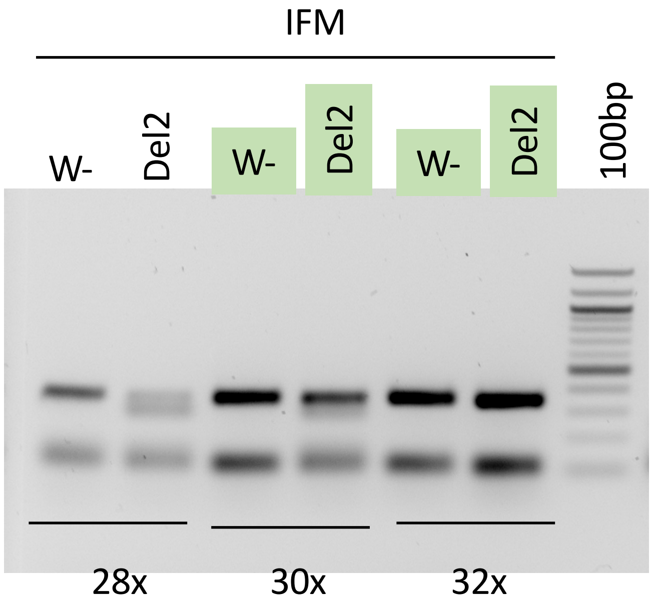

Gel: 200703

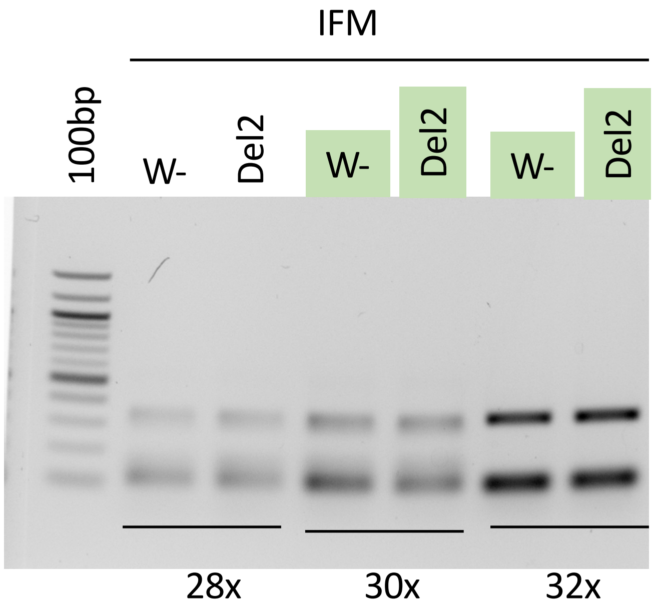

Gel: 200710_b

Nx of one gel = same PCR reaction, from which aliquots were taken at the respective cycle number

Rbfox1

Rbfox1 levels in bru1Del2 IFMs

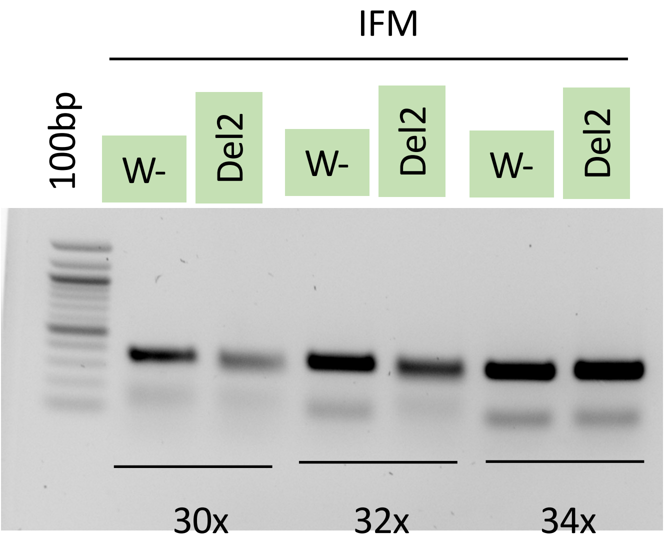

Gel: 200710

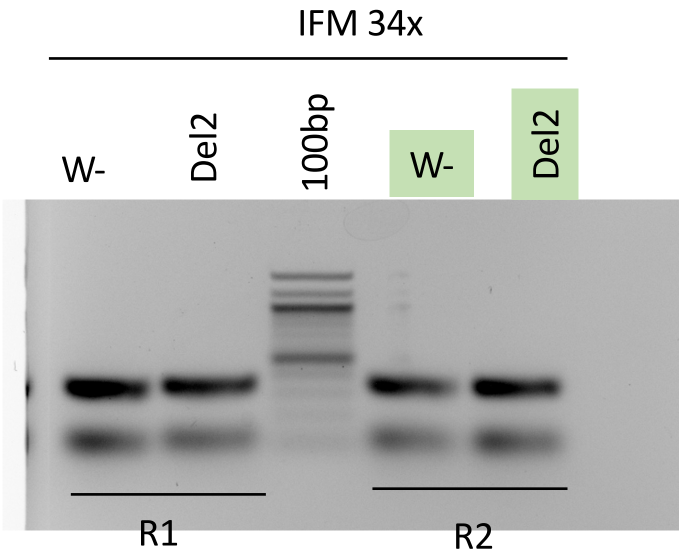

Gel:200714

Rbfox1

Rbfox1 levels in Bru1 OE IFMs

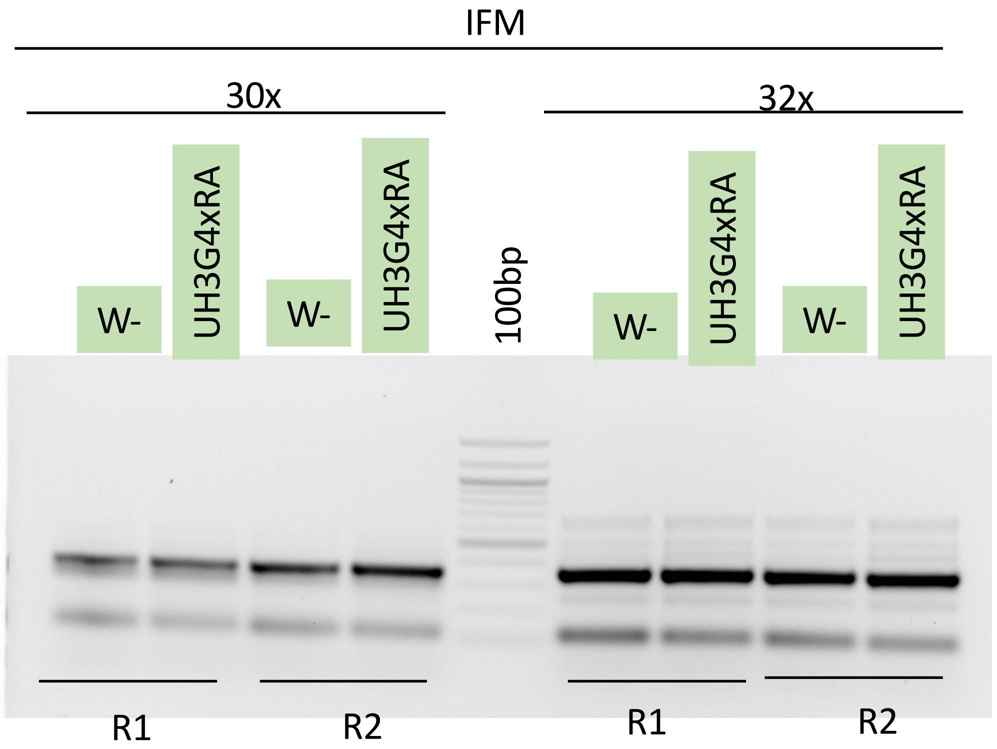

Gel: 201230_Fox_IFM_R1_R2_Left30x_Right_32x

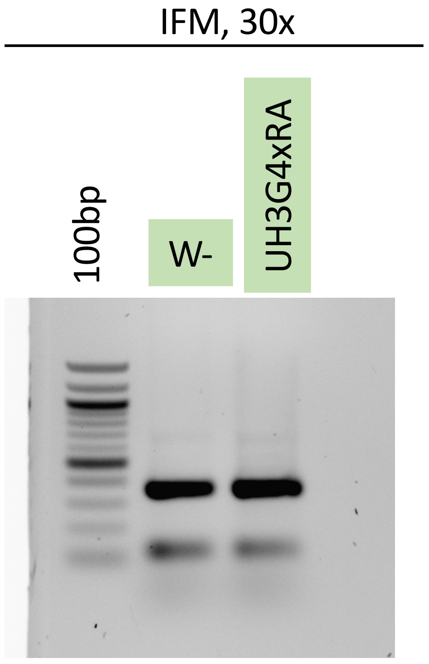

Gel: 210104

Rbfox1

Rbfox1 levels in bru1Del2 and Bru1 OE tissues

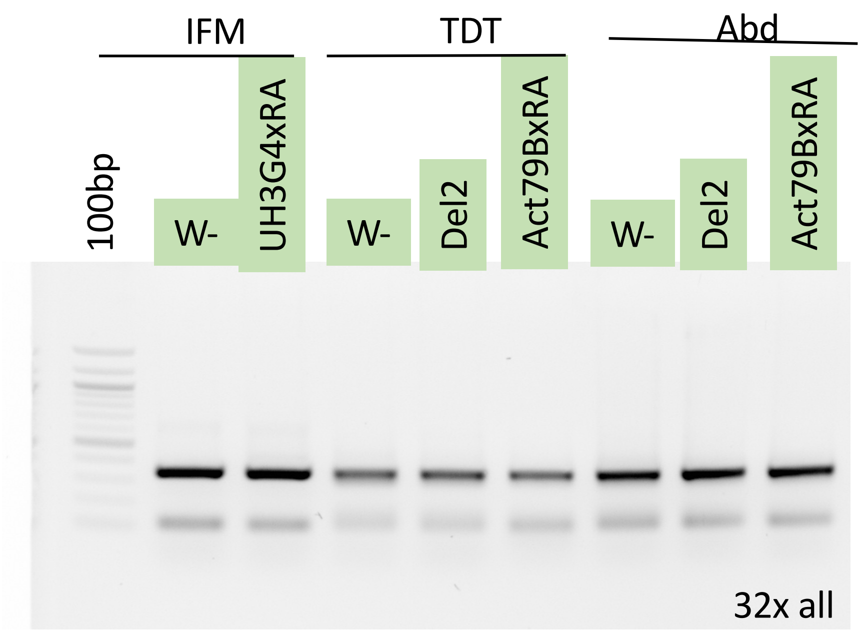

Gel:201229_Fox1_1(Left)

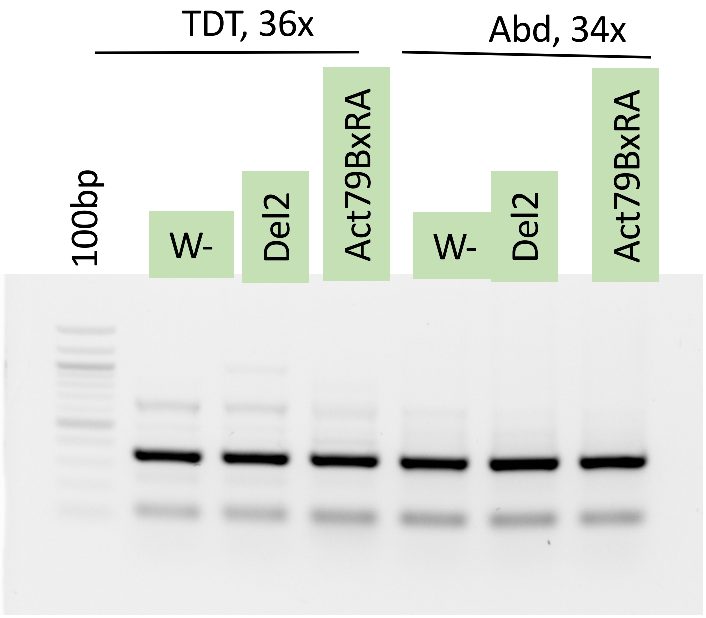

Gel:201229_Fox1_2

Rbfox1

Rbfox1 levels in bru1Del2 and Bru1 OE tissues

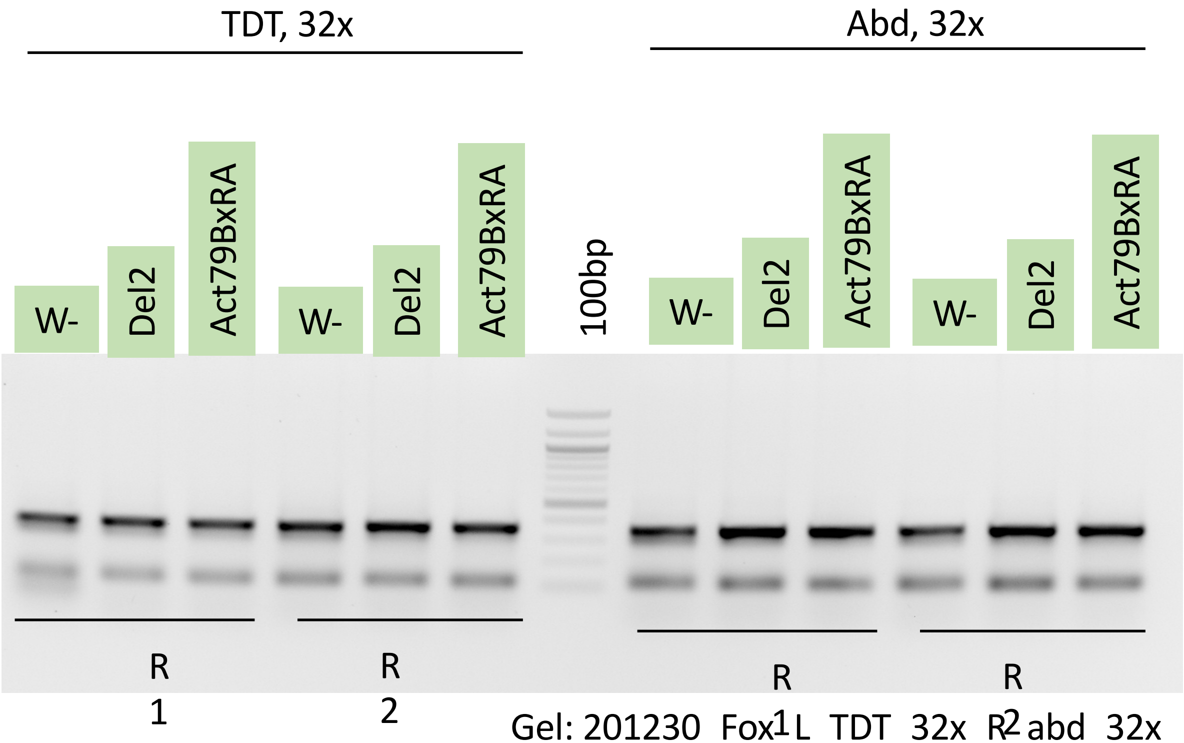

Gel: 201230_Fox1_L_TDT_32x_R2_abd_32x

Rbfox1

Rbfox1 levels in bru1Del2 and Bru1 OE tissues

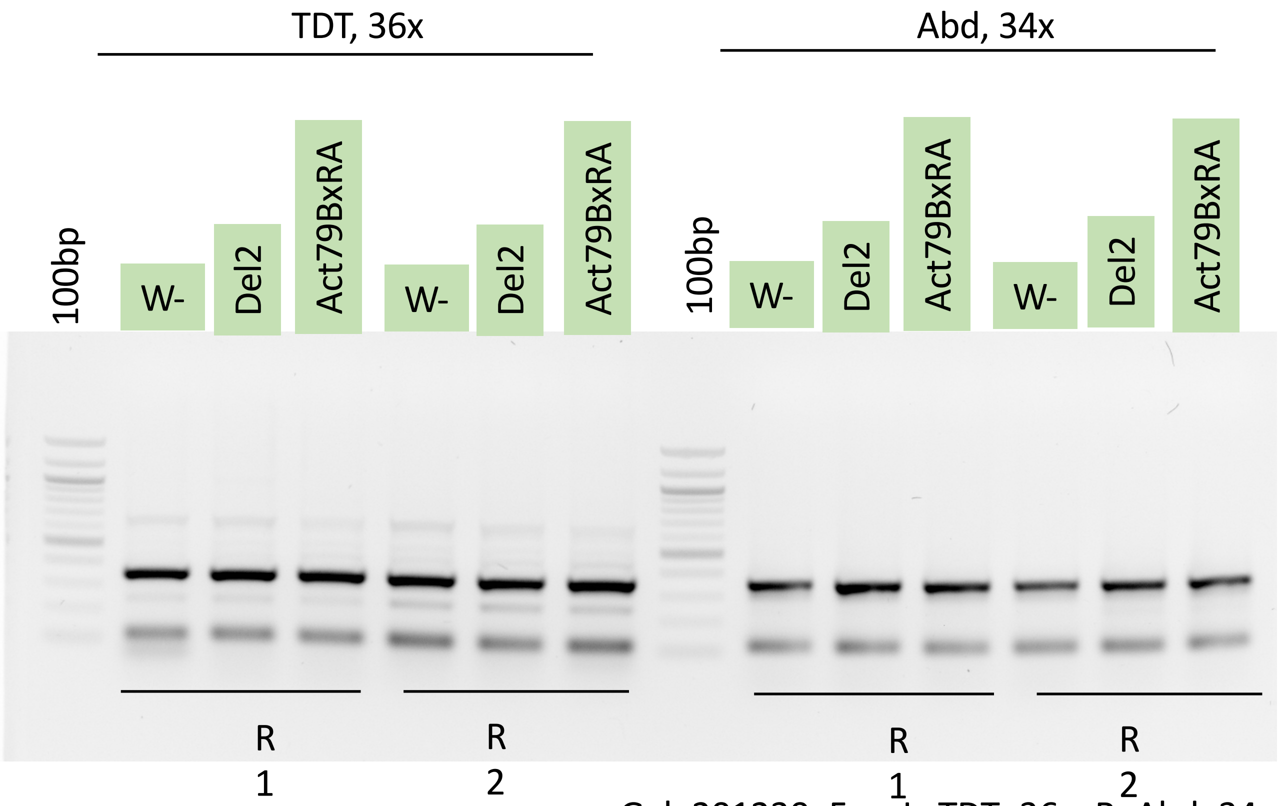

Gel: 201230_Fox_L_TDT_36x_R_Abd_34x

Rbfox1

Original Western blots

Bru1 levels in *Rbfox1-IR*^{27286} tissues

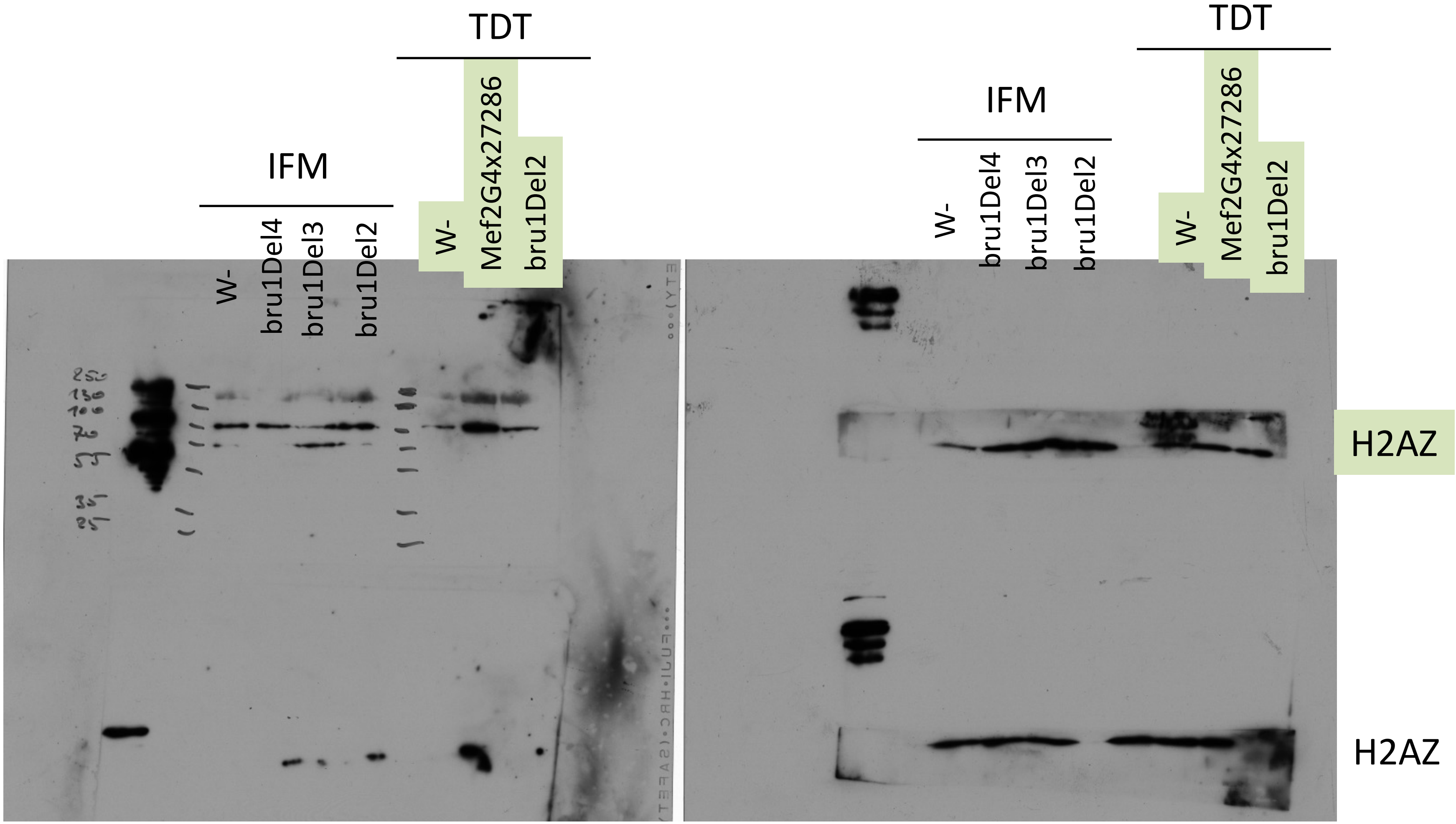

Blot: 200408_1

Bru1 levels in *Rbfox1-IR*[27286] tissues

Note: No H2AZ was run on this blot, thus not included in quantifications

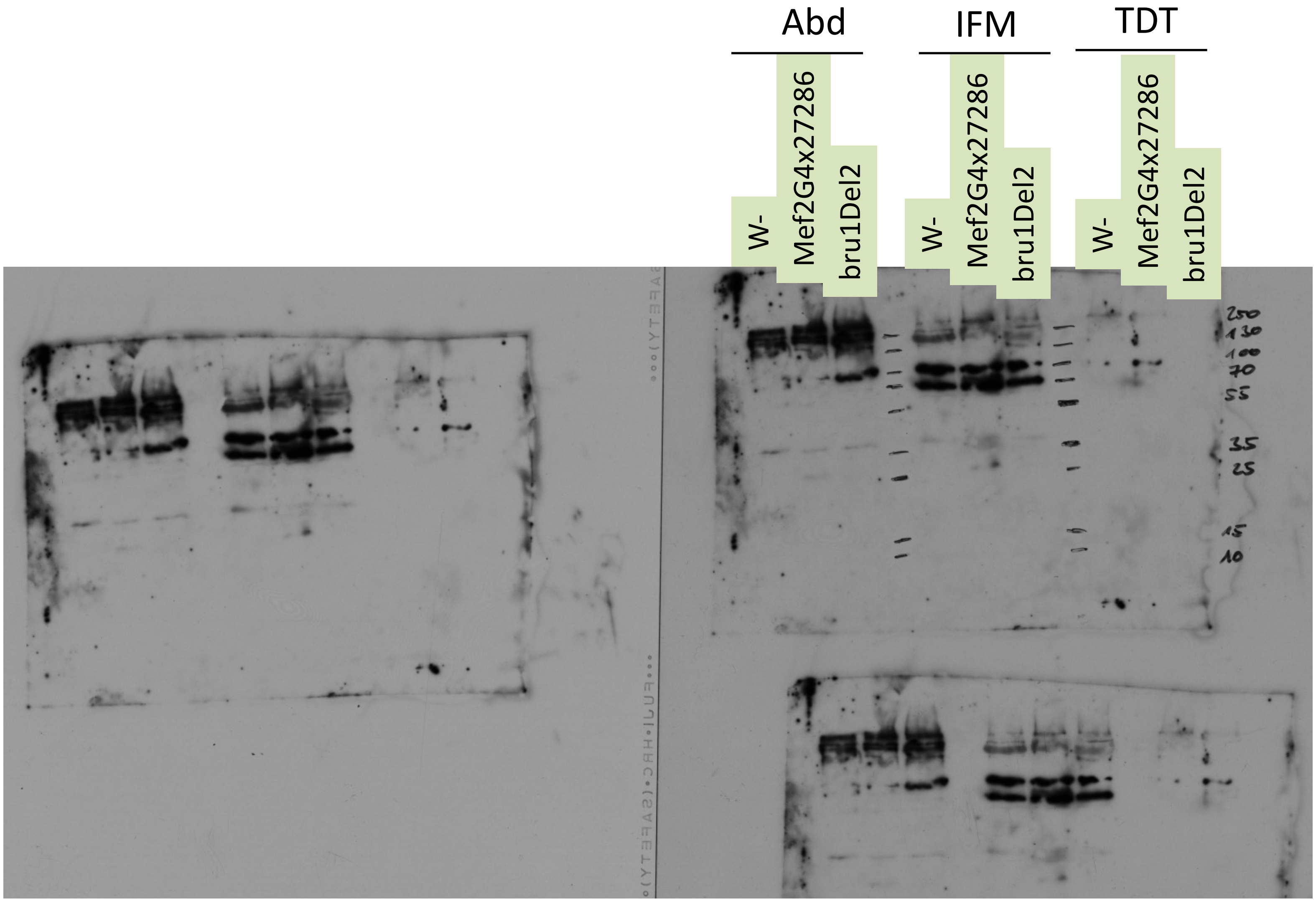

Blot: 200428

Bru1 levels in *Rbfox1-IR*[27286] tissues

Blot: 200519

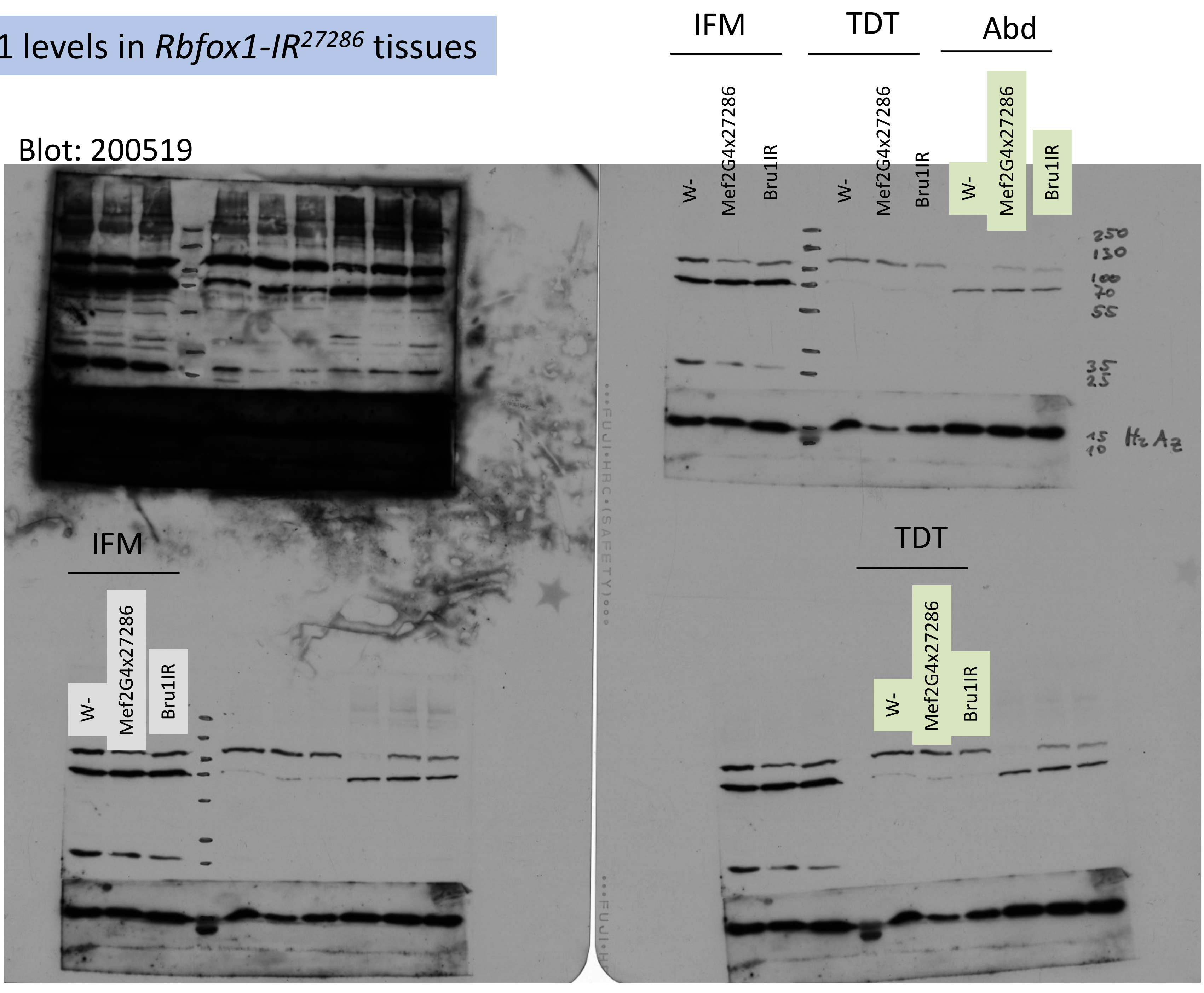

Bru1 levels in *Rbfox1-IR*[27286] tissues

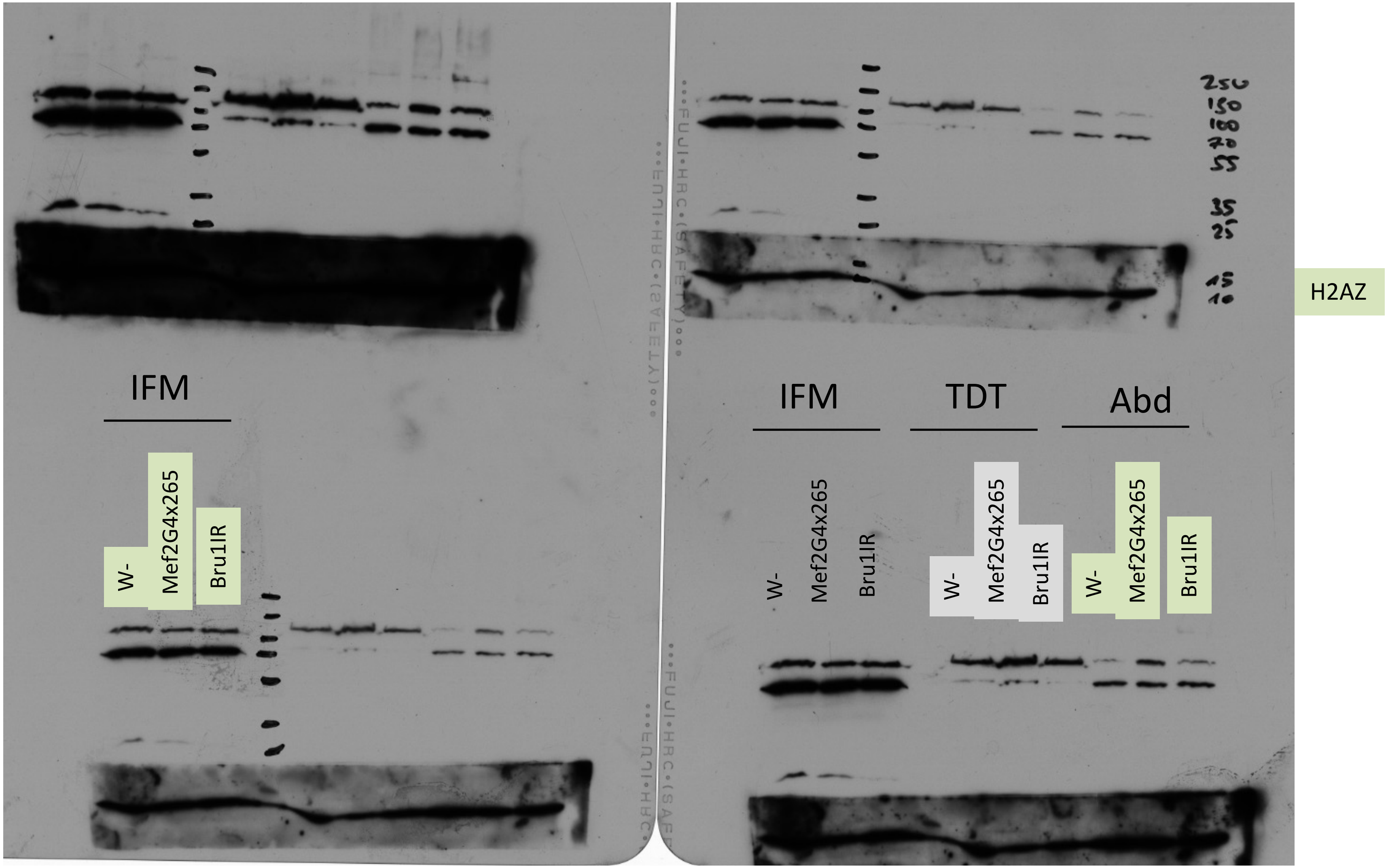

Blot: 200526

Bru1 levels in *Rbfox1-IR*^{27286} tissues

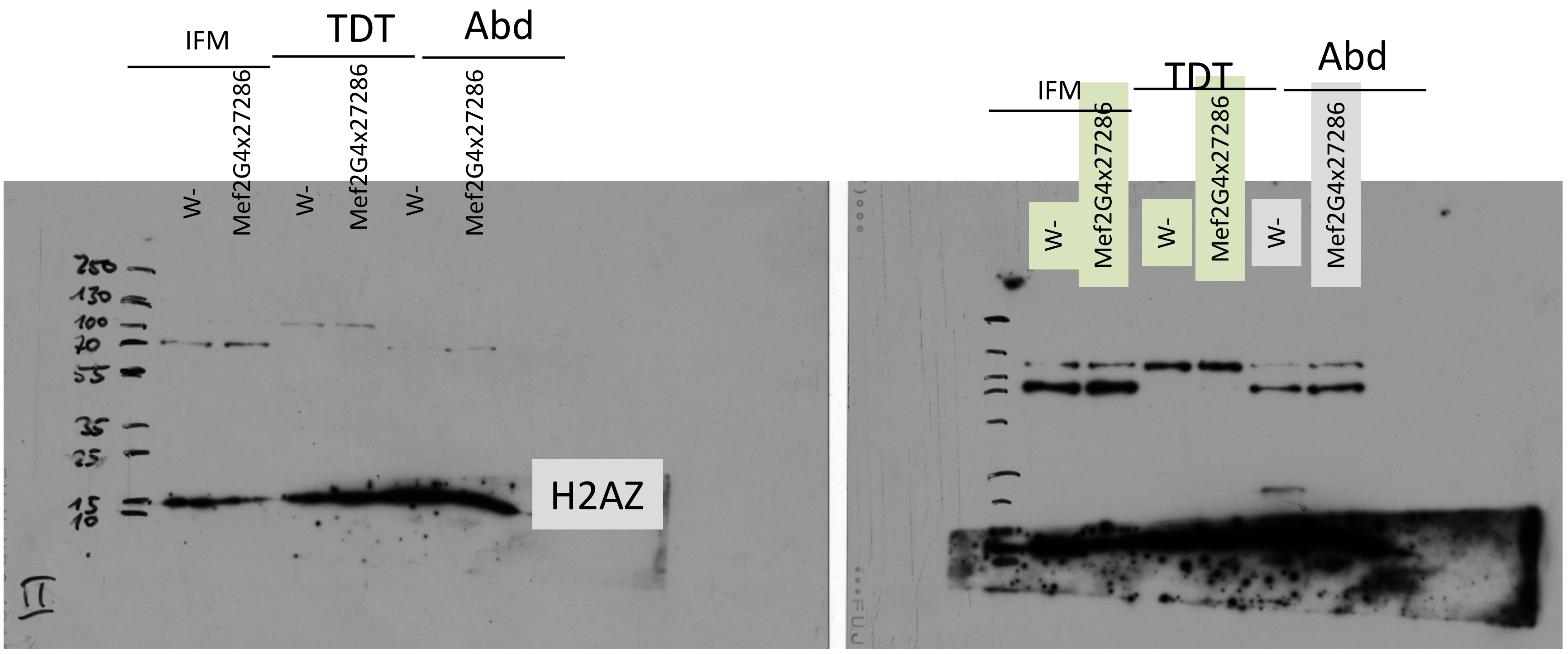

Blot: 201015

Bru1 levels in *Rbfox1-IR*^{KK110518} tissues

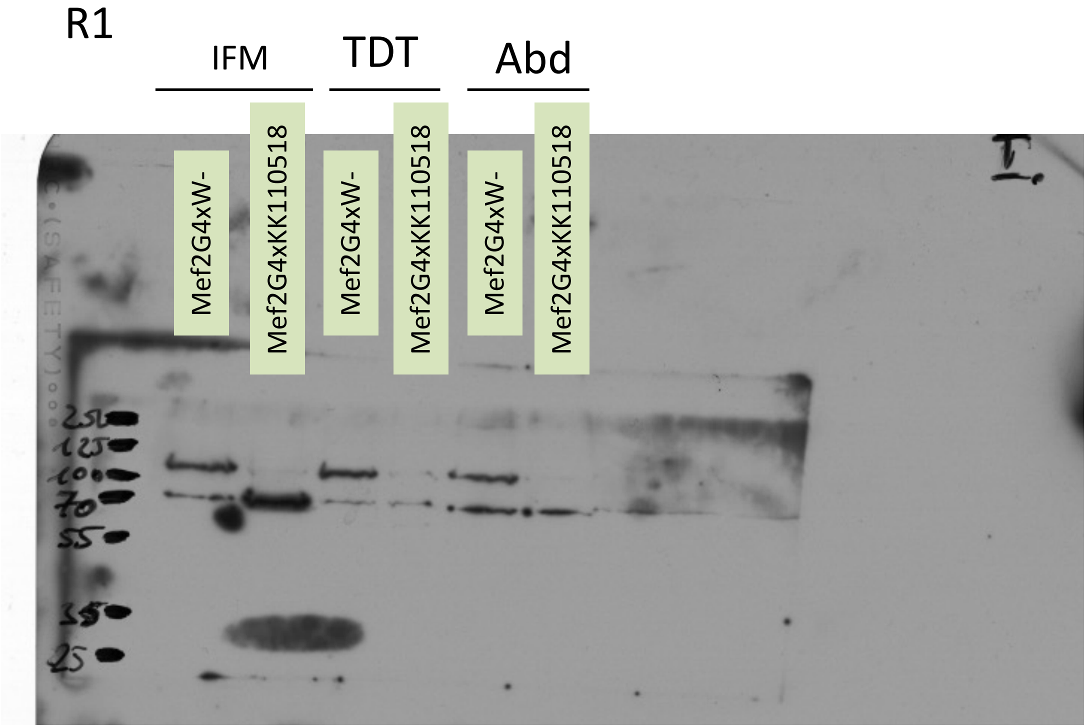

Blot: 201104_I_exp

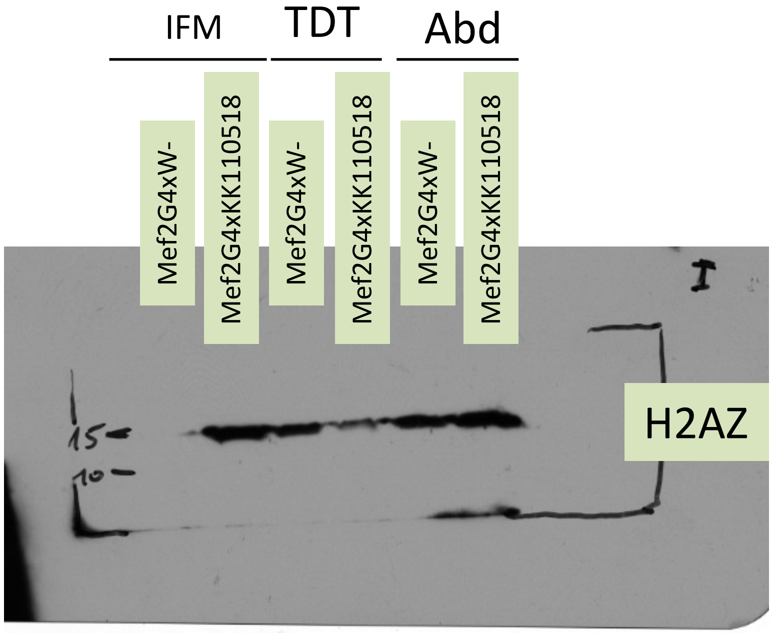

Blot: 201104_I_H2AZ

Bru1 levels in *Rbfox1-IR*^{KK110518} tissues

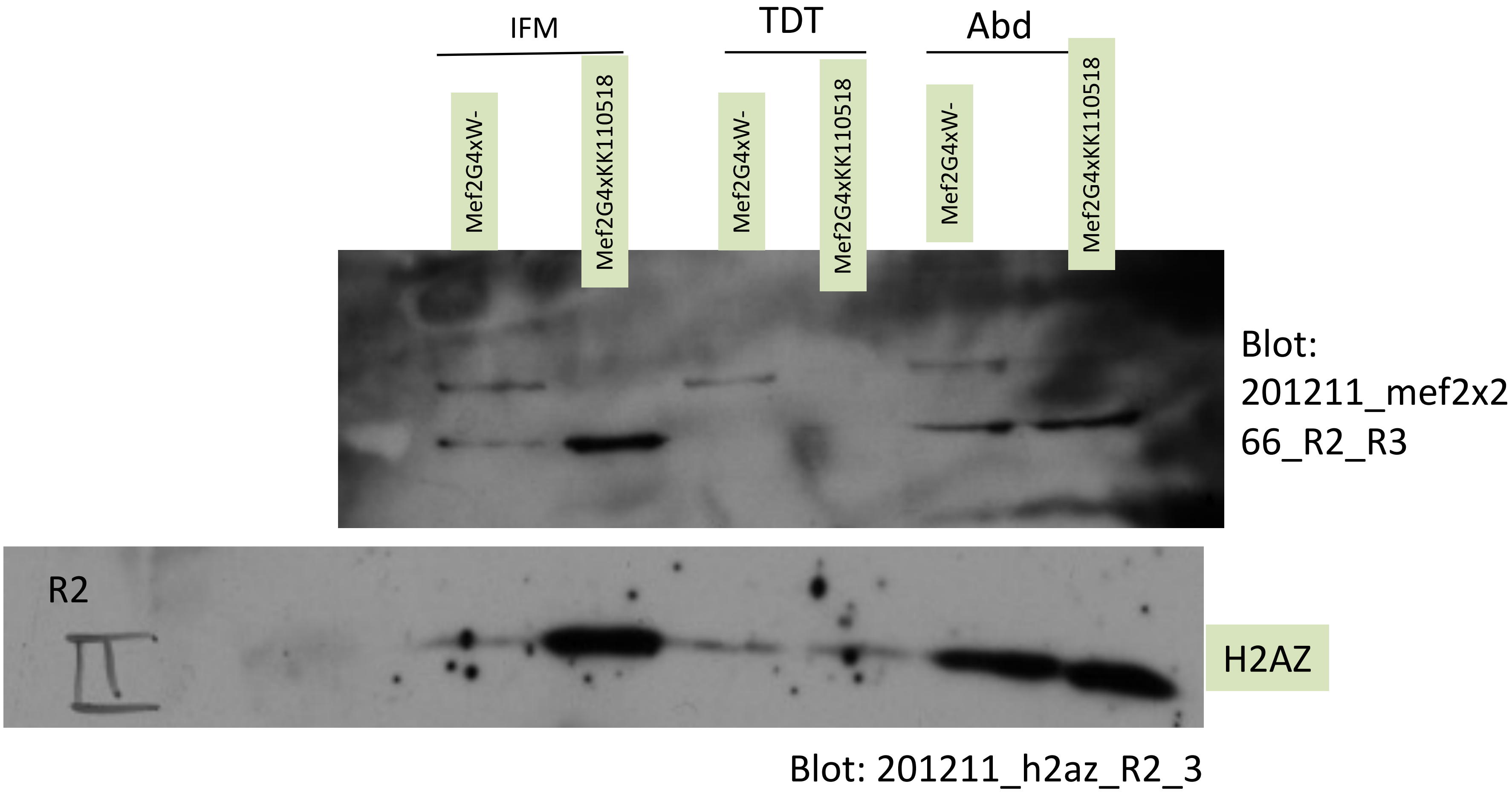

Bru1 levels in *Rbfox1-IR*^{KK110518} tissues

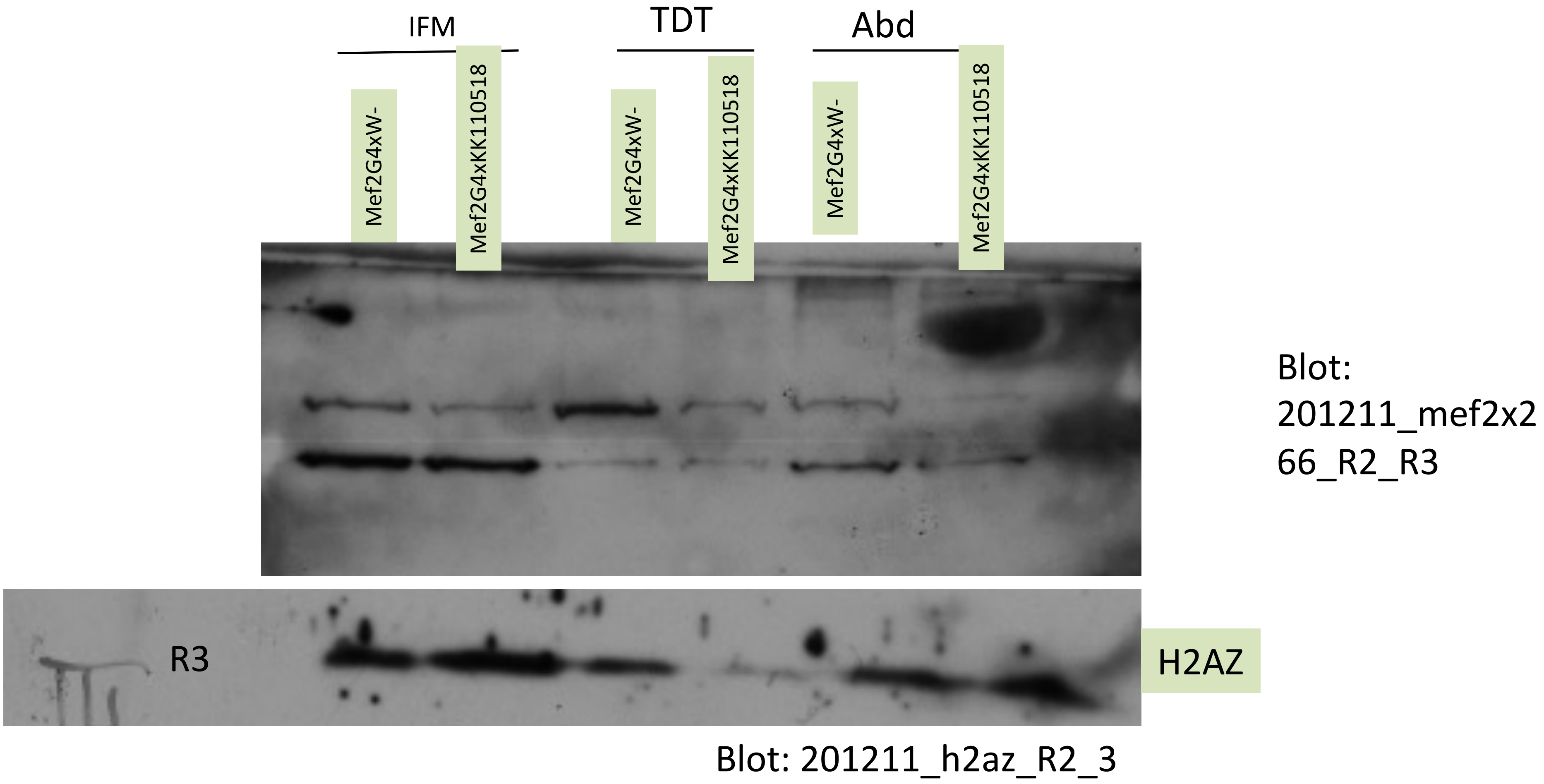

Bru1 levels in *Rbfox1-IR*^{KK110518} tissues

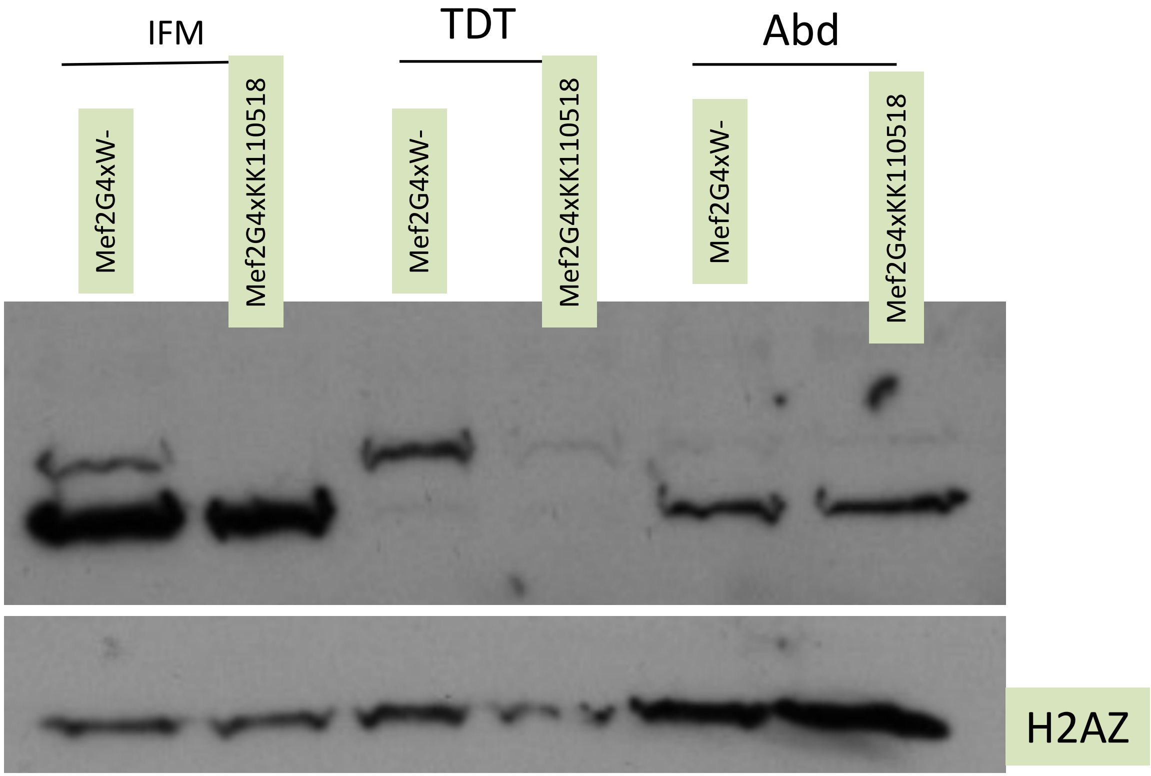

Blot: 201216_266_R4
