# Supplementary material for: Rbfox1 is required for myofibril development and maintaining fiber type–specific isoform expression in Drosophila muscles
Source: Life Sci Alliance. 2022 Jan 7;5(4):e202101342. doi: 10.26508/lsa.202101342 (PMC8742874; doi:10.26508/lsa.202101342)

Bruno1 Western Blot

Anti-DIV antibody verification

(rabbit polyclonal antibody raised against the divergent domain (DIV) of Bru1)

Bru1 in *bru1* mutant flies

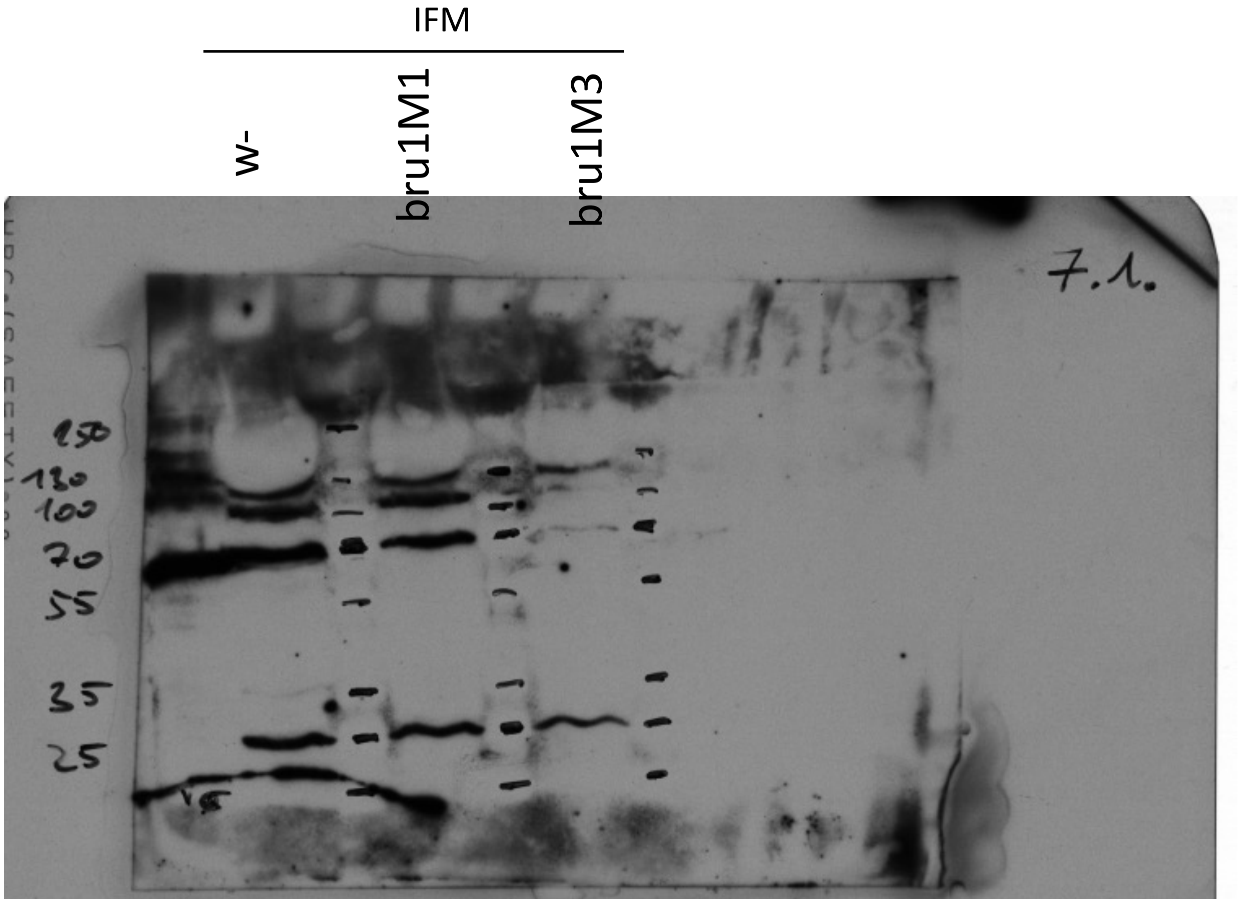

Blot: 200107_aDIV

Bru1 in wildtype tissues

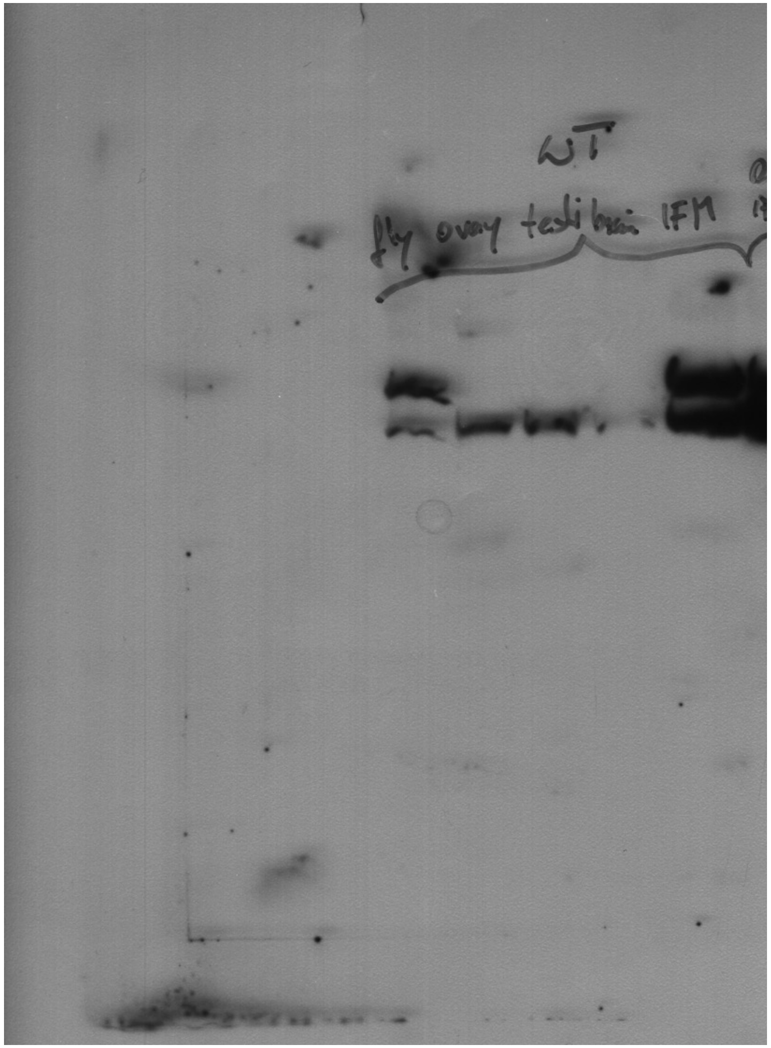

Blot: 2021_11_09_aDIV

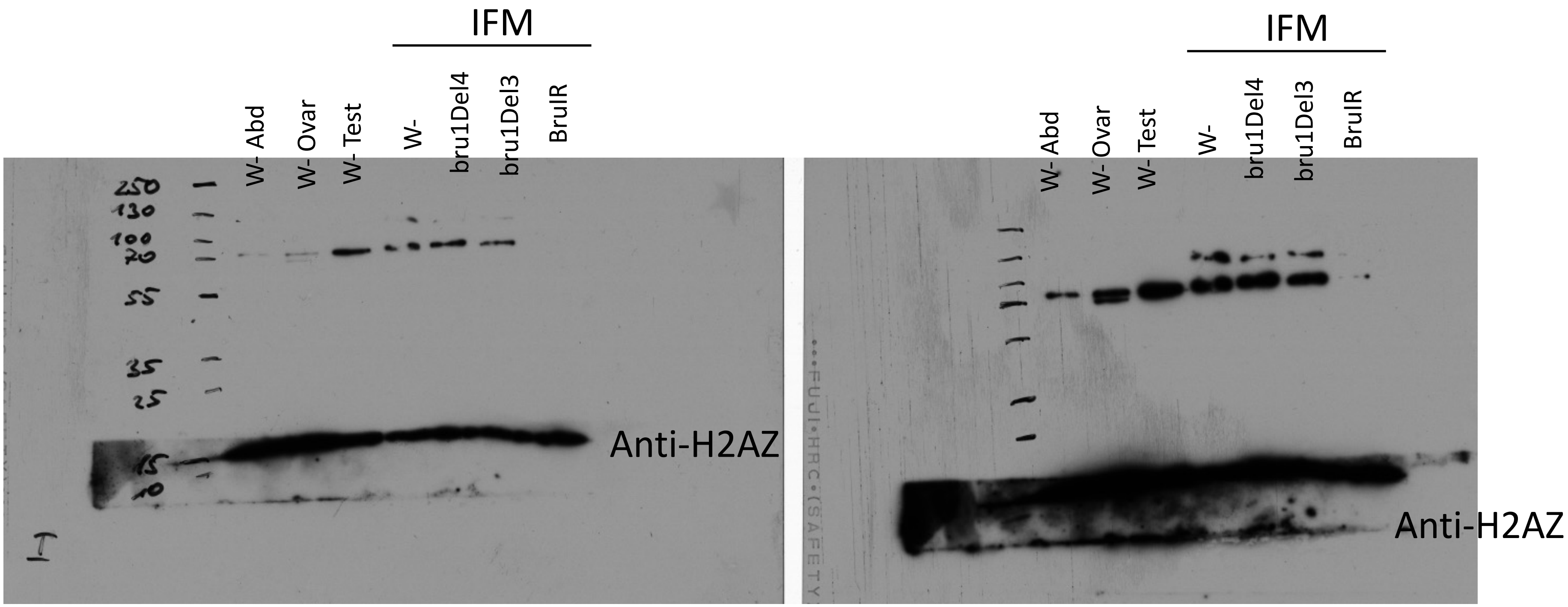
IFM
W- Abd
W- Ovar
W- Test
W-
bru1Del4
bru1Del3
BruIR
250
130
100
70
55
35
25
15
10
Anti-H2AZ
IFM
W- Abd
W- Ovar
W- Test
W-
bru1Del4
bru1Del3
BruIR
Anti-H2AZ

Blot: 201015

Bru1 in wildtype tissues

bru1Del2
bru1Del2 (line 1)
bru1Del2 (line 2)
bru1Del2 (line 3)
bru1Del1 (line 1)
bru1Del3 (line 1)
bru1Del3 (line 2)
w1118
Purified Bru1-PA

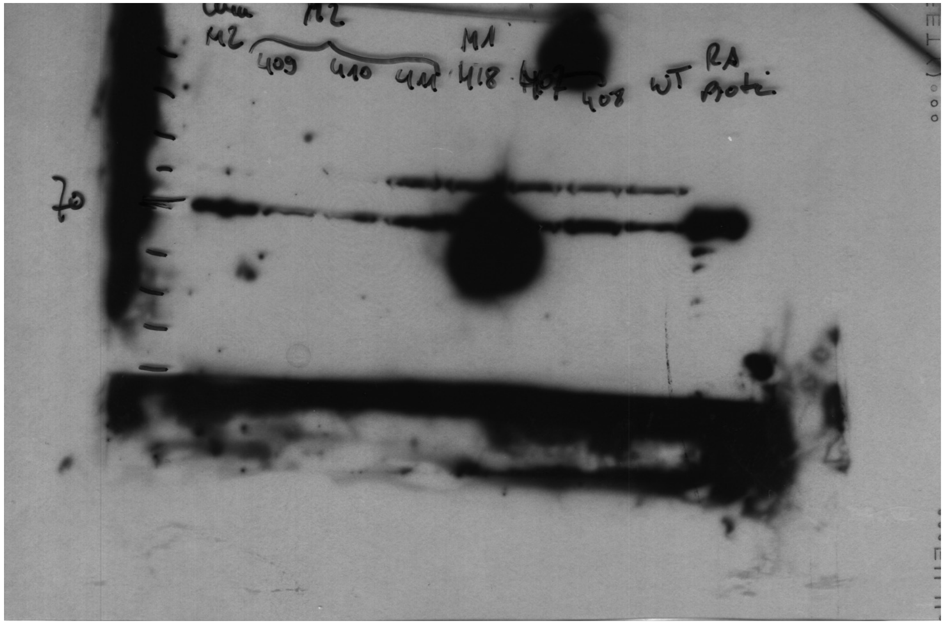

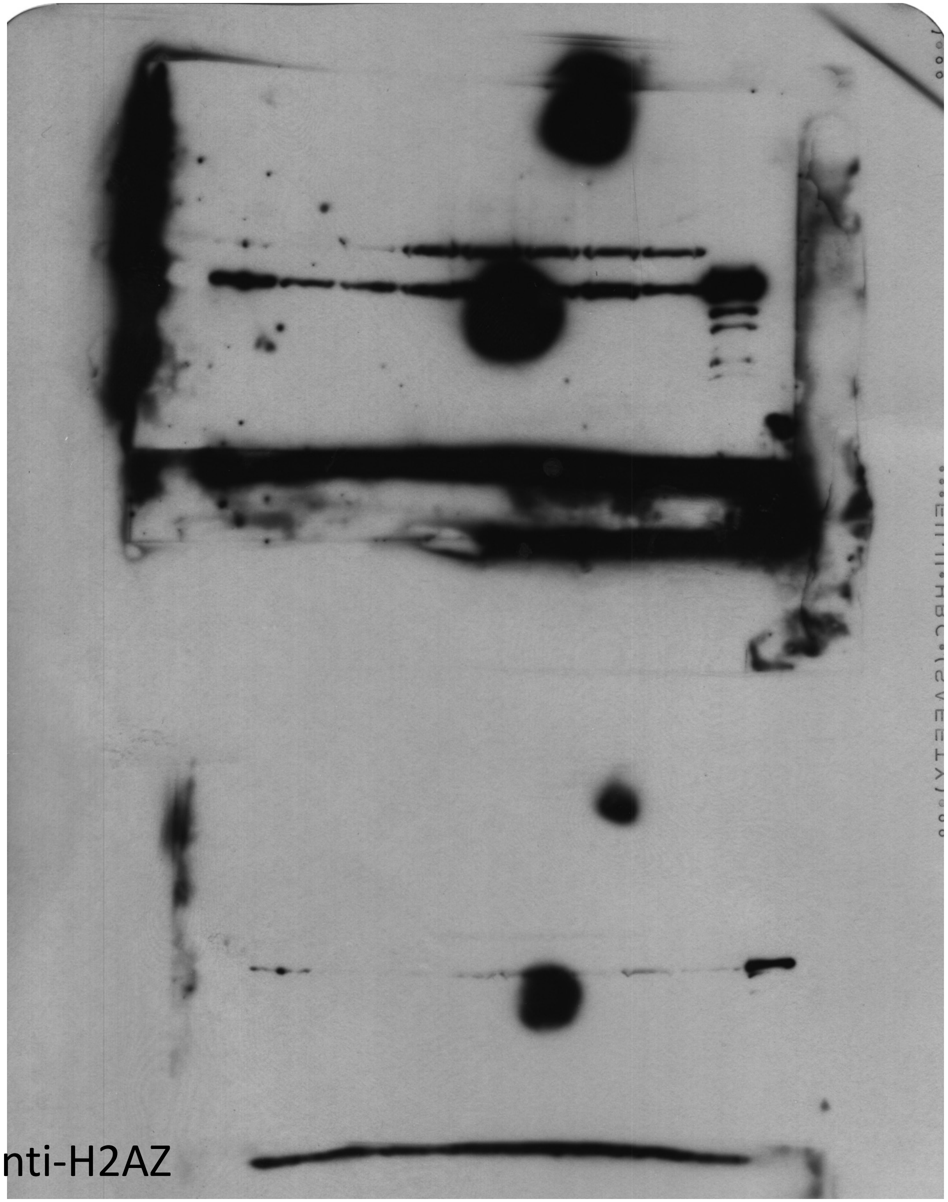

Anti-H2AZ

Blot: 2021_11_22_aDIV

Bruno1 Del2 verification

dsRed cassette

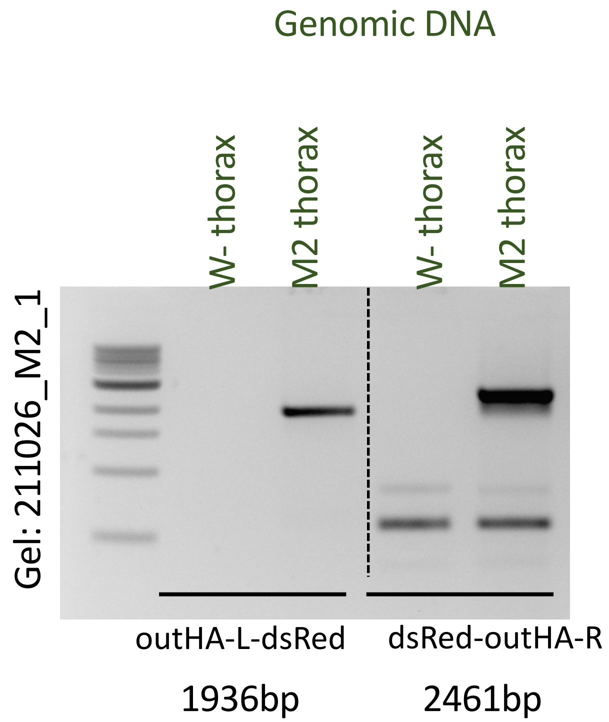

Out HA-L: gcacttgcagtttgcgataa

SV40_R: gactagttgatcataatcagcca

dsRed_F: ccacaaggccctgaagctga

Out HA-R: GAATCTCGCCAGAACCAAGA

Anti-Bru1 (DIV domain)

Ex13_F: GGTACCCAAAAAGAGAAGGAGCA[illegible]

Ex17_R: TTCAATCTGCTTTCCCGC

cDNA

W- thorax | M2 thorax

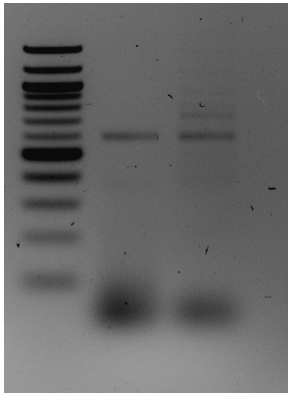

Ex13 – ex17: 588 bp
DIV domain

Gel:211007_bru1DIV

Genomic DNA

W- | M3 | M2

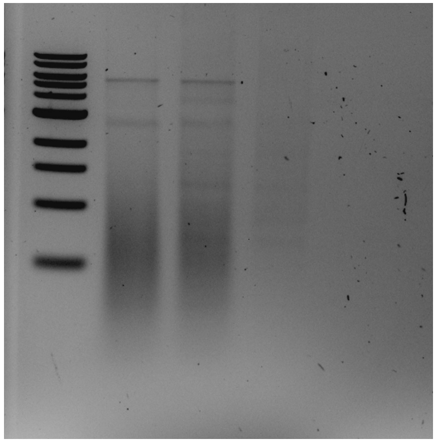

Ex13 – ex17: 5715 bp

Gel: 211012_gPCR_DIV

Ex13_F: GGTACCCAAAAAGAGAAGGAGCAG

Ex13_R: AACAACTGGTGCTGCAGT

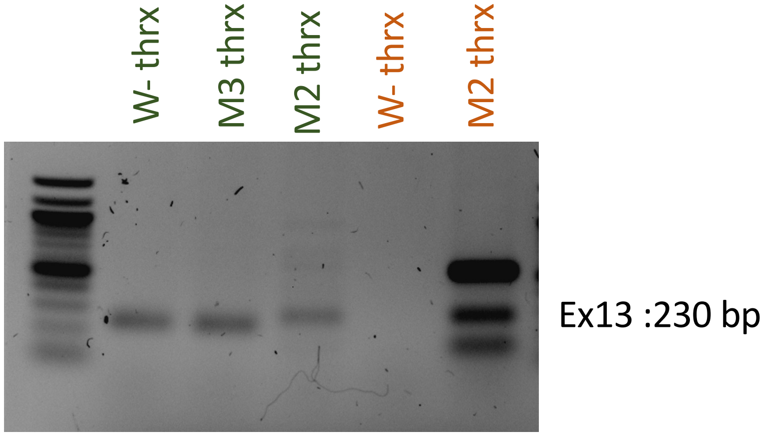

Gel: 211012_gPCR_RT_DIV

Ex14_F: CCAGAATCTAGCGGCCATT

Ex14_R: CAGCCGTGTTGGTCAGTT

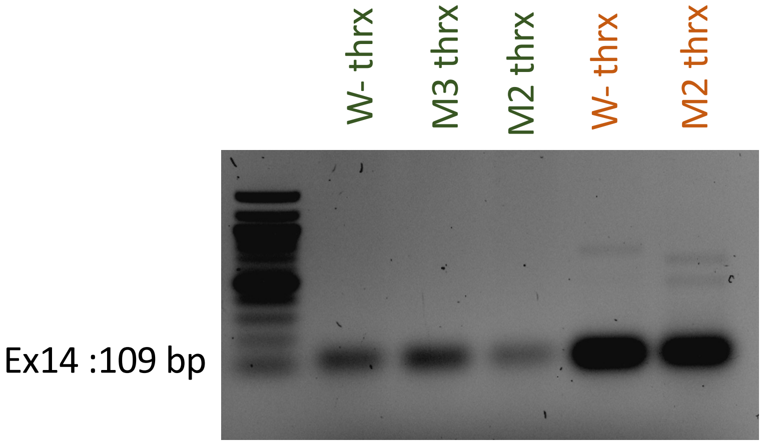

Gel: 211012_gPCR_RT_DIV

cDNA   Genomic DNA

Ex12_F: TTTGAAGTCCACGGAGCC

Ex14_R: AGCCGTGTTGGTCAGTTGGG

Ex8_F: ACGAGACACGCCGCCCTGAAAGC

Ex19_R: CCTCTACTCGATGTGTCCGTTTAACCTT

Ex21_R: TCAAGATGTGCAACGACAAATAGCC

Ex13_R: TTGCGTATCGGCGAACTTGACGACC

Ex12_R: TCCGTGGACTTCAAATAGCTTGCG

W- thrx | M3 thrx | M2 thrx | W- thrx | M2 thrx | W- thrx | M2 thrx | W- thrx | M2 thrx | W- thrx | M2 thrx | W- thrx | M2 thrx

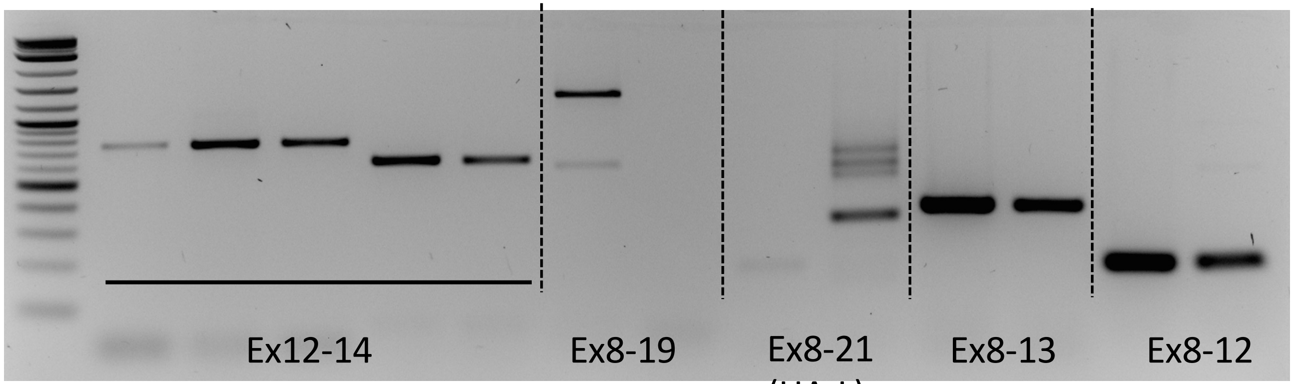

Gel: 211020_DIV_1

(HA-L)

Expected length:
gDNA: 775
cDNA: 615

1260bp

2346bp

Seq: Ex12-14 on gDNA = no differences between wt and M2

cDNA

Genomic DNA

ex19_F: AAGGTTAAACGGACACATCGAGTAGAGG

Ex20_R: GGCACAGATTTCATGTCTTTTTCTGC

ex18_F:TGCTTCGGGTTTGTCTCCTTCGAC

Ex21_R: TCAAGATGTGCAACGACAAATAGCC

ex18_F:TGCTTCGGGTTTGTCTCCTTCGAC

Ex18_R: AGTAGGGCTTCGAGTCCTTG

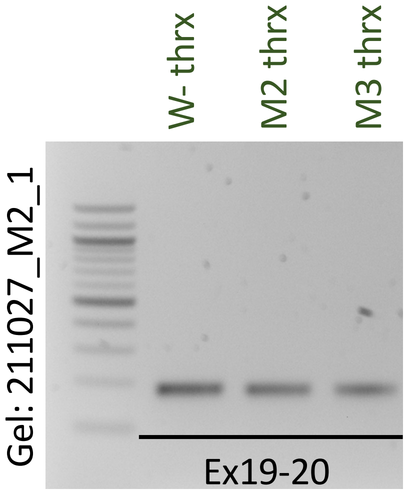

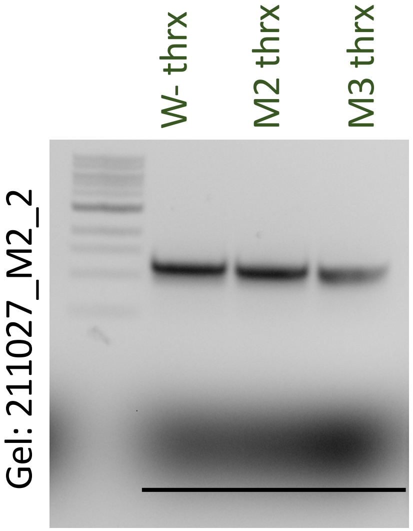

Ex18-21(HA-R)

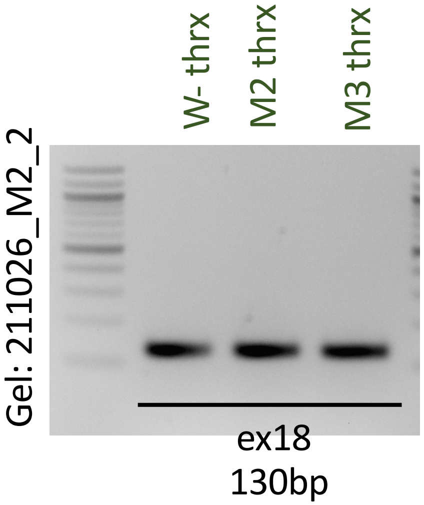

ex18
130bp

Seq: ex18-21= no changes in coding seq of RRM3

Genomic DNA

cDNA

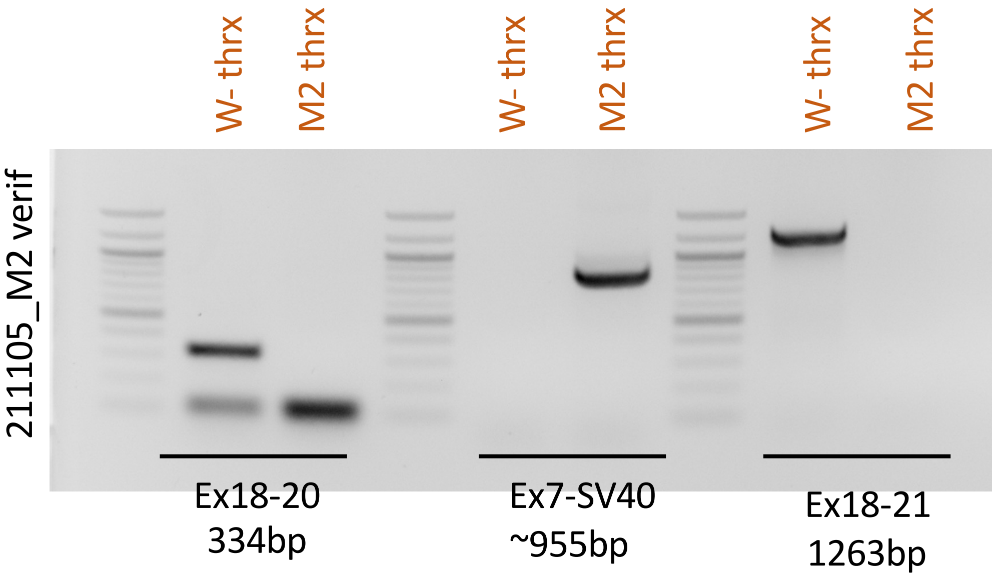

ex18_F:TGCTTCGGGTTTGTCTCCTTCGAC

Ex20_R: GGCACAGATTTCATGTCTTTTTCTGC

Ex7_F: AGCCTGCCGAATAGTCCC

SV40_R: gactagttgatcataatcagcca

ex18_F:TGCTTCGGGTTTGTCTCCTTCGAC

Ex21_R: TCAAGATGTGCAACGACAAATAGCC

RP49: GGTATCgacaacagagtgcg

RP49: GAACTTCTTGAATCCGGTGGG

cDNA

Ex14_F: CCAGAATCTAGCGGCCATT

Ex18_R: AGTAGGGCTTCGAGTCCTTG

Oligo_dT: TTTTTTTTTTTTTTTTTT

SV40_R: gactagttgatcataatcagcca

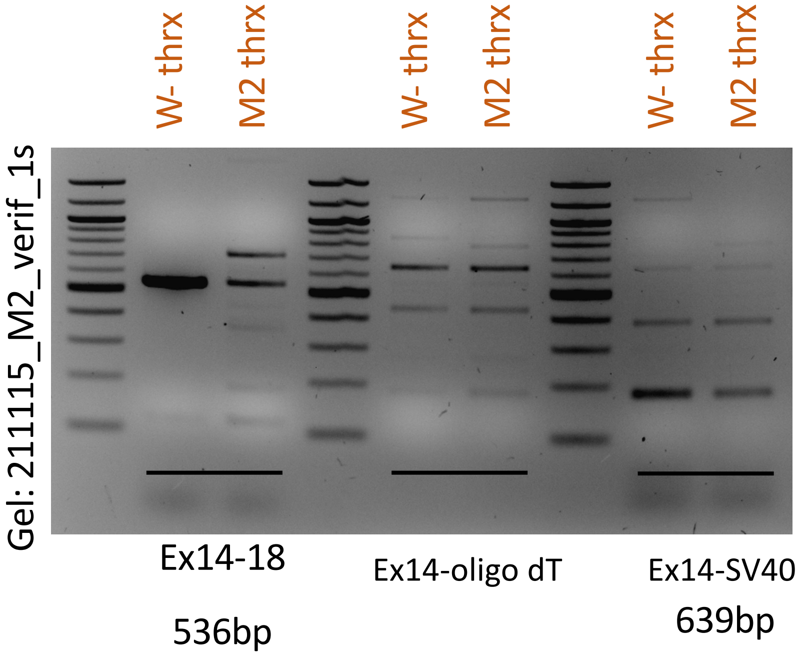

Supplement: Supplementary file 12 [file LSA-2021-01342_SdataFS5.pdf]
