# Supplementary material for: Rbfox1 is required for myofibril development and maintaining fiber type–specific isoform expression in Drosophila muscles
Source: Life Sci Alliance. 2022 Jan 7;5(4):e202101342. doi: 10.26508/lsa.202101342 (PMC8742874; doi:10.26508/lsa.202101342)
Supplement: Supplementary file 13 [file LSA-2021-01342_SdataF6.pdf]

**Raw data used to generate plots**

**Figure panel**

| | | band1 (TDT) | | band2 (midIFM) | | band3 (short) | |
|---|---|---|---|---|---|---|---|
| 6U | Zasp52_wt | 0 | 0 | 55.07893 | 62.38281 | 44.92107 | 37.61719 |
| | Zasp52_KK | 0 | 0 | 10.17483 | 14.29639 | 89.82517 | 85.70361 |
| | Zasp52_bru | 0 | 0 | 14.16892 | 10.87659 | 85.83108 | 89.12341 |
| | Zasp52_bru | 0 | 0 | 4.018706 | 5.198926 | 95.98129 | 94.80107 |
| | Zasp52_UH | 0 | 0 | 65.15297 | 73.24667 | 34.84703 | 26.75333 |
| | Zasp52_wt | 60.42228 | 38.10364 | 8.273291 | 22.89991 | 31.30443 | 38.99645 |
| | Zasp52_KK | 30.48278 | 16.65872 | 6.574435 | 10.37849 | 62.94279 | 72.96279 |
| | Zasp52_bru | 51.58188 | 32.81203 | 8.280225 | 24.72443 | 40.1379 | 42.46354 |
| | Zasp52_Ac | 0 | 0 | 62.11828 | 76.92252 | 37.88173 | 23.07748 |
| | Zasp52_wt | 0 | 0 | 3.327714 | 4.149055 | 96.67229 | 95.85094 |
| | Zasp52_KK | 0 | 0 | 1.758191 | 5.442959 | 98.24181 | 94.55704 |
| | Zasp52_bru | 0 | 0 | 2.476291 | 3.915824 | 97.52371 | 96.08418 |

| | | band1 (1kb) | | | band2 (200bp) | | | band3 (100bp) | | |
|---|---|---|---|---|---|---|---|---|---|---|
| 6V | Zasp66_wt | 0 | 0 | 0 | 63.02933 | 67.03788 | 77.0198 | 36.97067 | 32.96212 | 22.9802 |
| | Zasp66_KK | 3.416893 | 1.337118 | 10.84018 | 0 | 0 | 0 | 96.58311 | 98.66288 | 89.15982 |
| | Zasp66_bru | 0 | 0 | 0 | 100 | 100 | 100 | 0 | 0 | 0 |
| | Zasp66_bru | 9.103911 | 4.672287 | 9.307065 | 5.833469 | 0 | 10.44243 | 85.06262 | 95.32771 | 80.25051 |
| | Zasp66_UH | 1.265471 | 1.687153 | 0 | 40.78331 | 40.55687 | 44.91733 | 57.95122 | 57.75597 | 55.08267 |
| | Zasp66_wt | 1.234645 | 1.013914 | | 98.76535 | 98.98609 | | 0 | 0 | |
| | Zasp66_KK | 47.87674 | 35.85826 | | 43.19991 | 44.08342 | | 8.923356 | 20.05832 | |
| | Zasp66_bru | 1.160377 | 1.165845 | | 98.83962 | 98.83416 | | 0 | 0 | |
| | Zasp66_Ac | 2.756936 | 1.155097 | | 45.46736 | 44.9465 | | 51.77571 | 53.8984 | |

| | | band1 (710bp) | | band2 (621bp) | | band3 (492bp) | |
|---|---|---|---|---|---|---|---|
| 6W | Zasp67_wt | 14.11247 | 28.89946 | 85.88753 | 71.10054 | 0 | 0 |
| | Zasp67_KK | 25.34982 | 16.4256 | 54.11581 | 49.1871 | 20.53437 | 34.38729 |
| | Zasp67_bru | 15.87212 | 30.57929 | 84.12788 | 69.42071 | 0 | 0 |
| | Zasp67_bru | 0 | 0 | 16.58523 | 21.60676 | 83.41477 | 78.39324 |
| | Zasp67_UH | 21.54266 | 38.69117 | 78.45734 | 61.30883 | 0 | 0 |
| | Zasp67_wt | 25.10568 | 28.92491 | 74.89432 | 71.07509 | 0 | 0 |
| | Zasp67_KK | 13.90531 | 26.07943 | 40.97799 | 17.95645 | 45.11671 | 55.96411 |
| | Zasp67_bru | 21.54854 | 30.64786 | 78.45146 | 69.35214 | 0 | 0 |
| | Zasp67_Ac | 35.47668 | 42.88006 | 64.52332 | 57.11994 | 0 | 0 |

| | w- | Mef2xw- | Mef2x27286 | M2 | M2,27286 |
|---|---|---|---|---|---|
| 6Q | 2.7063 | 2.9318 | 2.4432 | 1.5673 | |
| | 2.7063 | 2.9318 | 2.5869 | 1.59 | 0 |
| | 2.7063 | 2.9318 | 2.5869 | 1.6385 | 0 |
| | 2.7063 | 2.9318 | 2.5869 | 1.673 | 0 |
| | 2.7063 | 2.9318 | 2.7063 | 1.6753 | |
| | 2.7063 | 3.1983 | 2.7063 | 1.693 | |
| | 2.7063 | 3.1983 | 2.7063 | 1.699 | |
| | 2.9318 | 3.1983 | 2.7063 | 1.7166 | |
| | 2.9318 | 3.1983 | 2.7486 | 1.7166 | |
| | 2.9318 | 3.1983 | 2.7486 | 1.7166 | |
| | 3.1983 | 3.1983 | 2.7486 | 1.719 | |
| | 3.1983 | 3.1983 | 2.7486 | 1.726 | |
| | 3.1983 | 3.1983 | 2.7486 | 1.728 | |
| | 3.1983 | 3.1983 | 2.7486 | 1.728 | |
| | 3.1983 | 3.1983 | 2.7486 | 1.7591 | |
| | 3.1983 | 3.1983 | 2.7486 | 1.7591 | |
| | 3.1983 | 3.1983 | 2.9318 | 1.7591 | |
| | 3.1983 | 3.1983 | 2.9318 | 1.762 | |
| | 3.1983 | 3.1983 | 2.9318 | 1.801 | |
| | 3.3829 | 3.1983 | 2.9318 | 1.8024 | |
| | 3.3829 | 3.1983 | 2.9318 | 1.8024 | |
| | 3.3829 | 3.1983 | 2.9318 | 1.8024 | |
| | 3.3829 | 3.1983 | 2.9318 | 1.804 | |
| | 3.3829 | 3.1983 | 2.9318 | 1.839 | |
| | 3.5182 | 3.1983 | 2.9318 | 1.858 | |
| | 3.5182 | 3.1983 | 2.9318 | 1.867 | |
| | 3.5182 | 3.5182 | 2.9318 | 1.871 | |
| | 3.5182 | 3.5182 | 2.9318 | 1.878 | |
| | 3.5182 | 3.5182 | 3.1412 | 1.878 | |
| | 3.5182 | 3.5182 | 3.1412 | 1.89 | |

| | | | | |
|---|---|---|---|---|
| 3.5182 | 3.5182 | 3.1412 | 1.948 | |
| 3.5182 | 3.5182 | 3.1412 | 1.9545 | |
| 3.5182 | 3.5182 | 3.1983 | 1.96 | |
| 3.5182 | 3.5182 | 3.1983 | 1.993 | |
| | 3.5182 | 3.1983 | 2.027 | |
| | 3.5182 | 3.1983 | 2.027 | |
| | 3.5182 | 3.1983 | 2.063 | |
| | 3.5182 | 3.1983 | 2.066 | |
| | 3.5182 | 3.1983 | 2.0695 | |
| | 3.5182 | 3.1983 | 2.0695 | |
| | 3.5182 | 3.1983 | 2.0695 | |
| | 3.5182 | 3.1983 | 2.0695 | |
| | 3.5182 | 3.1983 | 2.098 | |
| | 3.5182 | 3.1983 | 2.1205 | |
| | 3.5182 | 3.1983 | 2.137 | |
| | 3.5182 | 3.1983 | 2.166 | |
| | 3.5182 | 3.1983 | 2.1989 | |
| | 3.5182 | 3.1983 | 2.1989 | |
| | 3.5182 | 3.1983 | 2.1989 | |
| | 3.5182 | 3.1983 | 2.1989 | |
| | 3.5182 | 3.1983 | 2.1989 | |
| | 3.5182 | 3.1983 | 2.199 | |
| | 3.5182 | 3.1983 | 2.2 | |
| | 3.5182 | 3.1983 | 2.2 | |
| | | 2.362 | 2.201 | |
| | | 2.407 | 2.201 | |
| | | 2.449 | 2.201 | |
| | | 2.534 | 2.208 | |
| | | 2.535 | 2.233 | |
| | | 2.537 | 2.24 | |
| | | 2.545 | 2.253 | |
| | | 2.577 | 2.302 | |
| | | 2.577 | 2.339 | |
| | | 2.577 | 2.375 | |
| | | 2.578 | 2.439 | |
| | | 2.611 | 2.44 | |
| | | 2.611 | 2.442 | |
| | | 2.615 | 2.482 | |
| | | 2.62 | 2.513 | |
| | | 2.622 | 2.513 | |
| | | 2.646 | 2.58 | |
| | | 2.646 | | |
| | | 2.648 | | |
| | | 2.651 | | |
| | | 2.663 | | |
| | | 2.664 | | |
| | | 2.668 | | |
| | | 2.68 | | |
| | | 2.681 | | |
| | | 2.682 | | |
| | | 2.706 | | |
| | | 2.706 | | |
| | | 2.706 | | |
| | | 2.706 | | |
| | | 2.706 | | |
| | | 2.707 | | |
| | | 2.707 | | |
| | | 2.749 | | |
| | | 2.749 | | |
| | | 2.749 | | |
| | | 2.749 | | |
| | | 2.749 | | |
| | | 2.752 | | |
| | | 2.783 | | |
| | | 2.783 | | |
| | | 2.784 | | |
| | | 2.784 | | |
| | | 2.792 | | |
| | | 2.793 | | |
| | | 2.834 | | |

| | | | | |
|---|---|---|---|---|
| | | 2.836 | | |
| | | 2.852 | | |
| | | 2.852 | | |
| | | 2.852 | | |
| | | 2.854 | | |
| | | 2.879 | | |
| | | 2.886 | | |
| | | 2.886 | | |
| | | 2.886 | | |
| | | 2.886 | | |
| | | 2.888 | | |
| | | 2.889 | | |
| | | 2.889 | | |
| | | 2.92 | | |
| | | 2.921 | | |
| | | 2.921 | | |
| | | 2.955 | | |
| | | 2.955 | | |
| | | 2.956 | | |
| | | 2.958 | | |
| | | 2.989 | | |
| | | 2.989 | | |
| | | 2.989 | | |
| | | 2.991 | | |
| | | 3.024 | | |
| | | 3.024 | | |
| | | 3.024 | | |
| | | 3.025 | | |
| | | 3.027 | | |
| | | 3.058 | | |
| | | 3.059 | | |
| | | 3.06 | | |
| | | 3.061 | | |
| | | 3.063 | | |
| | | 3.092 | | |
| | | 3.126 | | |
| | | 3.127 | | |
| | | 3.195 | | |
| | | 3.23 | | |
| | | 3.264 | | |
| | | 3.298 | | |
| | | 3.3 | | |
| | | 3.333 | | |
| | | 3.367 | | |
| | | 3.368 | | |
| | | 3.368 | | |
| | | 3.401 | | |
| | | 3.539 | | |

| | w- | Mef2xw- | Mef2x27286 | M2 | M2,27286 |
|---|---|---|---|---|---|
| **6R** | 1.0307 | 0.9276 | 1.0651 | 0.9276 | 0 |
| | 1.0307 | 0.9276 | 1.066 | 0.9276 | 0 |
| | 1.0307 | 0.9964 | 1.07 | 0.999 | 0 |
| | 1.0651 | 0.9964 | 1.0737 | 1.0307 | |
| | 1.0651 | 1.0307 | 1.1338 | 1.031 | |
| | 1.0994 | 1.0307 | 1.1338 | 1.0561 | |
| | 1.1166 | 1.0307 | 1.147 | 1.0561 | |
| | 1.1338 | 1.0307 | 1.1681 | 1.133 | |
| | 1.1338 | 1.0651 | 1.2025 | 1.138 | |
| | 1.1338 | 1.0651 | 1.2025 | 1.146 | |
| | 1.1596 | 1.0651 | 1.203 | 1.1681 | |
| | 1.1596 | 1.0651 | 1.2454 | 1.21 | |
| | 1.1596 | 1.0651 | 1.2454 | 1.215 | |
| | 1.1681 | 1.0651 | 1.248 | 1.244 | |
| | 1.1681 | 1.0994 | 1.271 | 1.272 | |
| | 1.1681 | 1.0994 | 1.2712 | 1.294 | |
| | 1.1681 | 1.0994 | 1.2884 | 1.294 | |
| | 1.1681 | 1.0994 | 1.2884 | 1.294 | |
| | 1.1681 | 1.0994 | 1.2884 | 1.294 | |
| | 1.1681 | 1.0994 | 1.3056 | 1.32 | |

| | | | | |
|---|---|---|---|---|
| 1.2025 | 1.1338 | 1.3056 | 1.3399 | |
| 1.2025 | 1.1338 | 1.331 | 1.4086 | |
| 1.2025 | 1.1338 | 1.332 | 1.412 | |
| 1.2025 | 1.1338 | 1.3399 | 1.424 | |
| 1.2025 | 1.1338 | 1.3399 | 1.511 | |
| 1.2025 | 1.1681 | 1.3743 | 1.58 | |
| 1.2025 | 1.1681 | 1.3743 | 1.609 | |
| 1.2025 | 1.1681 | 1.375 | 1.6148 | |
| 1.2369 | 1.1681 | 1.4086 | 1.678 | |
| 1.2369 | 1.1681 | 1.4086 | 1.684 | |
| 1.2454 | 1.2025 | 1.409 | 1.684 | |
| 1.3056 | 1.2025 | 1.409 | 1.758 | |
| | 1.2025 | 1.417 | 1.808 | |
| | 1.2025 | 1.417 | 1.833 | |
| | 1.2025 | 1.4172 | 1.863 | |
| | 1.2025 | 1.418 | 1.891 | |
| | 1.2369 | 1.418 | 1.892 | |
| | 1.2369 | 1.443 | 1.895 | |
| | 1.2369 | 1.443 | 1.917 | |
| | 1.2369 | 1.443 | 1.935 | |
| | 1.2369 | 1.443 | 1.963 | |
| | 1.2712 | 1.443 | 2.102 | |
| | 1.2712 | 1.443 | 2.11 | |
| | 1.2712 | 1.4774 | 2.201 | |
| | 1.2712 | 1.4774 | 2.272 | |
| | 1.2712 | 1.478 | 2.308 | |
| | 1.2712 | 1.503 | 2.342 | |
| | 1.2712 | 1.503 | 2.371 | |
| | 1.3056 | 1.5031 | 2.435 | |
| | 1.3056 | 1.5031 | 2.451 | |
| | 1.3056 | 1.5117 | 2.482 | |
| | 1.3056 | 1.5117 | 2.51 | |
| | 1.3056 | 1.5117 | 2.529 | |
| | 1.3056 | 1.512 | 2.59 | |
| | 1.3056 | 1.512 | 2.594 | |
| | 1.3056 | 1.513 | 2.605 | |
| | 1.3056 | 1.546 | 2.626 | |
| | 1.3056 | 1.546 | 2.817 | |
| | 1.3056 | 1.546 | 2.828 | |
| | 1.3399 | 1.5461 | 3.019 | |
| | 1.3743 | 1.5461 | 3.028 | |
| | 1.3743 | 1.5461 | 3.028 | |
| | 1.3743 | 1.547 | 3.338 | |
| | | 1.548 | | |
| | | 1.548 | | |
| | | 1.58 | | |
| | | 1.58 | | |
| | | 1.5804 | | |
| | | 1.581 | | |
| | | 1.581 | | |
| | | 1.581 | | |
| | | 1.582 | | |
| | | 1.584 | | |
| | | 1.6148 | | |
| | | 1.615 | | |
| | | 1.615 | | |
| | | 1.615 | | |
| | | 1.616 | | |
| | | 1.632 | | |
| | | 1.633 | | |
| | | 1.649 | | |
| | | 1.6491 | | |
| | | 1.6491 | | |
| | | 1.651 | | |
| | | 1.651 | | |
| | | 1.6749 | | |
| | | 1.675 | | |
| | | 1.683 | | |
| | | 1.683 | | |
| | | 1.684 | | |

| | | | | |
|---|---|---|---|---|
| | | 1.685 | | |
| | | 1.685 | | |
| | | 1.685 | | |
| | | 1.687 | | |
| | | 1.689 | | |
| | | 1.7179 | | |
| | | 1.7179 | | |
| | | 1.718 | | |
| | | 1.718 | | |
| | | 1.72 | | |
| | | 1.754 | | |
| | | 1.754 | | |
| | | 1.758 | | |
| | | 1.761 | | |
| | | 1.761 | | |
| | | 1.804 | | |
| | | 1.804 | | |
| | | 1.804 | | |
| | | 1.804 | | |
| | | 1.821 | | |
| | | 1.824 | | |
| | | 1.826 | | |
| | | 1.8467 | | |
| | | 1.8467 | | |
| | | 1.847 | | |
| | | 1.847 | | |
| | | 1.849 | | |
| | | 1.855 | | |
| | | 1.855 | | |
| | | 1.863 | | |
| | | 1.8896 | | |
| | | 1.89 | | |
| | | 1.89 | | |
| | | 1.892 | | |
| | | 1.894 | | |
| | | 1.924 | | |
| | | 1.933 | | |
| | | 1.96 | | |
| | | 1.976 | | |
| | | 2.018 | | |
| | | 2.019 | | |
| | | 2.061 | | |
| | | 2.105 | | |
| | | 2.233 | | |
| | | 2.319 | | |

| | w- | Mef2xw- | Mef2x27286 | M2 | Dbl |
|---|---|---|---|---|---|
| | 2.166 | 2.1989 | 1.683 | 2.237 | 2.267 |
| **6S** | 2.268 | 2.24 | 1.683 | 2.243 | 1.718 |
| | 2.269 | 2.339 | 1.7591 | 2.3454 | 1.751 |
| | 2.339 | 2.373 | 1.774 | 2.421 | 1.786 |
| | 2.339 | 2.405 | 1.821 | 2.44 | 1.787 |
| | 2.377 | 2.405 | 1.824 | 2.474 | 1.856 |
| | 2.443 | 2.405 | 1.855 | 2.475 | 1.858 |
| | 2.474 | 2.407 | 1.86 | 2.476 | 1.867 |
| | 2.492 | 2.474 | 1.86 | 2.485 | 1.891 |
| | 2.543 | 2.474 | 1.89 | 2.508 | 1.91 |
| | 2.577 | 2.474 | 1.891 | 2.513 | 1.926 |
| | 2.611 | 2.508 | 1.895 | 2.513 | 1.927 |
| | 2.611 | 2.508 | 1.895 | 2.536 | 1.9545 |
| | 2.625 | 2.509 | 1.925 | 2.543 | 1.962 |
| | 2.68 | 2.509 | 1.929 | 2.543 | 1.969 |
| | 2.681 | 2.513 | 1.929 | 2.543 | 1.993 |
| | 2.716 | 2.513 | 1.935 | 2.577 | 2.027 |
| | 2.749 | 2.513 | 1.9545 | 2.579 | 2.034 |
| | 2.749 | 2.513 | 1.9545 | 2.585 | 2.061 |
| | 2.783 | 2.513 | 1.9545 | 2.591 | 2.062 |
| | 2.784 | 2.513 | 1.9545 | 2.645 | 2.069 |
| | 2.786 | 2.513 | 1.9545 | 2.645 | 2.1 |
| | 2.792 | 2.513 | 1.9545 | 2.646 | 2.103 |

| | | | | |
|---|---|---|---|---|
| 2.819 | 2.513 | 1.958 | 2.649 | 2.114 |
| 2.852 | 2.513 | 1.959 | 2.681 | 2.13 |
| 2.3146 | 2.513 | 1.994 | 2.682 | 2.135 |
| 2.3454 | 2.513 | 1.994 | 2.7063 | 2.166 |
| 2.3454 | 2.514 | 2.012 | 2.7063 | 2.171 |
| 2.3454 | 2.542 | 2.028 | 2.7063 | 2.174 |
| 2.4432 | 2.543 | 2.028 | 2.7063 | 2.178 |
| 2.513 | 2.557 | 2.032 | 2.7063 | 2.186 |
| 2.513 | 2.575 | 2.032 | 2.7063 | 2.1989 |
| 2.513 | 2.578 | 2.051 | 2.7063 | 2.199 |
| 2.513 | 2.583 | 2.062 | 2.714 | 2.2 |
| 2.513 | 2.588 | 2.066 | 2.714 | 2.203 |
| 2.513 | 2.595 | 2.0695 | 2.749 | 2.203 |
| 2.5869 | 2.611 | 2.0695 | 2.749 | 2.207 |
| 2.5869 | 2.612 | 2.0695 | 2.783 | 2.208 |
| 2.5869 | 2.613 | 2.0695 | 2.783 | 2.236 |
| 2.5869 | 2.646 | 2.0695 | 2.784 | 2.237 |
| 2.7063 | 2.646 | 2.096 | 2.817 | 2.306 |
| 2.7063 | 2.646 | 2.096 | 2.818 | 2.3454 |
| 2.7063 | 2.654 | 2.13 | 2.828 | 2.375 |
| 2.7063 | 2.664 | 2.13 | 2.831 | 2.383 |
| 2.7063 | 2.681 | 2.13 | 2.872 | 2.391 |
| 2.7063 | 2.681 | 2.137 | 2.893 | 2.493 |
| 2.7063 | 2.698 | 2.137 | 2.899 | 2.551 |
| 2.7063 | 2.7063 | 2.14 | 2.922 | 2.578 |
| 2.7063 | 2.7063 | 2.165 | 2.9318 | 2.714 |
| 2.7063 | 2.7063 | 2.166 | 2.9318 | 2.736 |
| 2.7063 | 2.7063 | 2.174 | 2.9318 | 2.749 |
| 2.7063 | 2.7063 | 2.186 | 2.9318 | 2.749 |
| 2.7063 | 2.7063 | 2.192 | 2.9318 | 2.75 |
| 2.7063 | 2.7063 | 2.1989 | 2.9318 | 2.762 |
| 2.7063 | 2.7063 | 2.1989 | 2.9318 | 2.8 |
| 2.7063 | 2.7063 | 2.1989 | 2.9318 | 2.852 |
| 2.7063 | 2.7063 | 2.1989 | 2.9318 | 2.896 |
| 2.7063 | 2.7063 | 2.1989 | 2.9318 | 2.899 |
| 2.7063 | 2.709 | 2.1989 | 2.9318 | 2.903 |
| 2.7063 | 2.749 | 2.1989 | 2.9318 | 2.945 |
| 2.7486 | 2.749 | 2.1989 | 2.9318 | 2.962 |
| 2.9318 | 2.817 | 2.1989 | 2.9318 | 3.024 |
| 2.9318 | 2.9318 | 2.1989 | 2.933 | 3.101 |
| 2.9318 | 2.9318 | 2.1989 | 2.962 | 3.1983 |
| 2.9318 | 2.9318 | 2.1989 | 2.975 | 3.1983 |
| | 2.9318 | 2.199 | 2.99 | 3.23 |
| | 2.9318 | 2.226 | 2.994 | |
| | 2.9318 | 2.236 | 2.999 | |
| | 3.1983 | 2.236 | 3.005 | |
| | 3.1983 | 2.246 | | |
| | 3.1983 | 2.268 | | |
| | | 2.268 | | |
| | | 2.268 | | |
| | | 2.302 | | |
| | | 2.3454 | | |
| | | 2.371 | | |
| | | 2.474 | | |
| | | 2.475 | | |
| | | 2.513 | | |
| | | 2.513 | | |
| | | 2.513 | | |
| | | 2.513 | | |
| | | 2.578 | | |
| | | 2.7063 | | |
| | | 2.715 | | |

| | w- | Mef2xw- | Mef2x27286 | M2 | Dbl |
|---|---|---|---|---|---|
| **6T** | 3.124 | 2.509 | 1.4073 | 1.7591 | 2.619 |
| | 3.145 | 2.688 | 1.5992 | 1.8517 | 2.762 |
| | 3.1983 | 2.7063 | 1.6753 | 1.8517 | 2.817 |
| | 3.481 | 2.723 | 2.513 | 1.9545 | 2.824 |
| | 3.505 | 2.808 | 2.513 | 1.9545 | 2.825 |
| | 3.515 | 2.818 | 2.7063 | 1.9545 | 2.891 |

| | | | | |
|---|---|---|---|---|
| 3.5182 | 2.896 | 3.1983 | 2.0695 | 2.894 |
| 3.716 | 2.9318 | 3.335 | 2.166 | 2.93 |
| 3.721 | 2.9318 | 3.376 | 2.171 | 2.9318 |
| 3.745 | 3.07 | 3.471 | 2.1989 | 2.9318 |
| 3.78 | 3.092 | 3.481 | 2.1989 | 2.9318 |
| 3.849 | 3.127 | 3.5182 | 2.28 | 2.9318 |
| 3.9091 | 3.168 | 3.5182 | 2.3454 | 2.9318 |
| 3.9091 | 3.176 | 3.539 | 2.48 | 2.9318 |
| 3.986 | 3.267 | 3.584 | 2.513 | 2.9318 |
| 3.988 | 3.5182 | 3.618 | 2.513 | 2.9318 |
| 4.021 | 3.5182 | 3.642 | 2.513 | 2.933 |
| 4.026 | 3.579 | 3.65 | 2.513 | 2.955 |
| 4.091 | 3.745 | 3.684 | 2.513 | 2.967 |
| 4.123 | 3.802 | 3.711 | 2.534 | 2.988 |
| 4.157 | 3.9091 | 3.751 | 2.61 | 2.992 |
| 4.226 | 3.9091 | 3.78 | 2.9318 | 3.002 |
| 4.296 | 3.9091 | 3.814 | 2.9318 | 3.002 |
| 4.3977 | 3.917 | 3.85 | 2.9318 | 3.013 |
| 4.3977 | 3.929 | 3.863 | 2.9318 | 3.013 |
| 4.3977 | 3.929 | 3.9091 | 2.9318 | 3.026 |
| 4.4 | 3.952 | 3.922 | 2.9318 | 3.061 |
| 4.428 | 4.041 | 3.958 | 3.058 | 3.127 |
| 4.707 | 4.066 | 3.963 | 3.06 | 3.13 |
| 4.708 | 4.091 | 3.997 | 3.094 | 3.131 |
| 4.776 | 4.092 | 4.02 | 3.13 | 3.161 |
| 4.857 | 4.162 | 4.02 | 3.181 | 3.163 |
| 2.513 | 4.198 | 4.025 | 3.1983 | 3.176 |
| 2.513 | 4.237 | 4.054 | 3.1983 | 3.194 |
| 2.7063 | 4.36 | 4.072 | 3.1983 | 3.196 |
| | 4.3977 | 4.075 | 3.2 | 3.21 |
| | 4.3977 | 4.124 | 3.234 | 3.228 |
| | 4.435 | 4.345 | 3.236 | 3.267 |
| | 4.467 | 4.3977 | 3.266 | 3.276 |
| | 4.501 | 4.3977 | 3.313 | 3.298 |
| | 4.717 | 4.432 | 3.371 | 3.298 |
| | 4.948 | 4.57 | 3.402 | 3.298 |
| | | 4.61 | 3.47 | 3.32 |
| | | 4.857 | 3.5182 | 3.392 |
| | | | 3.5182 | 3.506 |
| | | | 3.5182 | 3.5182 |
| | | | 3.5182 | 3.5182 |
| | | | 3.539 | 3.5182 |
| | | | 3.539 | 3.52 |
| | | | 3.54 | 3.567 |
| | | | 3.552 | 3.567 |
| | | | 3.585 | 3.573 |
| | | | 3.587 | 3.575 |
| | | | 3.676 | 3.584 |
| | | | 3.678 | 3.642 |
| | | | 3.711 | 3.642 |
| | | | 3.722 | 3.642 |
| | | | 3.792 | 3.655 |
| | | | 3.826 | 3.676 |
| | | | 3.848 | 3.682 |
| | | | 3.849 | 3.709 |
| | | | 3.886 | 3.711 |
| | | | 3.89 | 3.824 |
| | | | 3.895 | 3.841 |
| | | | 3.9091 | 3.849 |
| | | | 3.9091 | 3.849 |
| | | | 3.9091 | 3.849 |
| | | | 3.9091 | 3.85 |
| | | | 3.9091 | 3.856 |
| | | | 3.956 | 3.883 |
| | | | 4.027 | 3.886 |
| | | | 4.058 | 3.917 |
| | | | 4.061 | 3.917 |
| | | | 4.092 | 3.976 |
| | | | 4.098 | 3.991 |
| | | | 4.134 | 3.991 |

| | | | | |
|---|---|---|---|---|
| | | | 4.158 | 4.054 |
| | | | 4.194 | 4.092 |
| | | | 4.228 | 4.125 |
| | | | 4.26 | 4.161 |
| | | | 4.3977 | 4.164 |
| | | | 4.3977 | 4.233 |
| | | | 4.3977 | 4.237 |
| | | | 4.3977 | 4.237 |
| | | | 4.3977 | 4.311 |
| | | | 4.3977 | 4.329 |
| | | | 4.3977 | 4.367 |
| | | | 4.3977 | 4.3977 |
| | | | 4.505 | 4.403 |
| | | | 4.507 | 4.432 |
| | | | 4.535 | 4.432 |
| | | | 4.54 | 4.433 |
| | | | 4.542 | 4.467 |
| | | | 4.551 | 4.502 |
| | | | 4.61 | 4.517 |
| | | | 4.639 | 4.673 |
| | | | 4.673 | 2.1989 |
| | | | 4.676 | 1.6753 |
| | | | 4.677 | 2.0695 |
| | | | 4.776 | 2.513 |
| | | | 4.81 | 1.3531 |
| | | | 4.949 | |

# RT-PCR gels of alternative splice events

Figure 6 panels

Zasp66

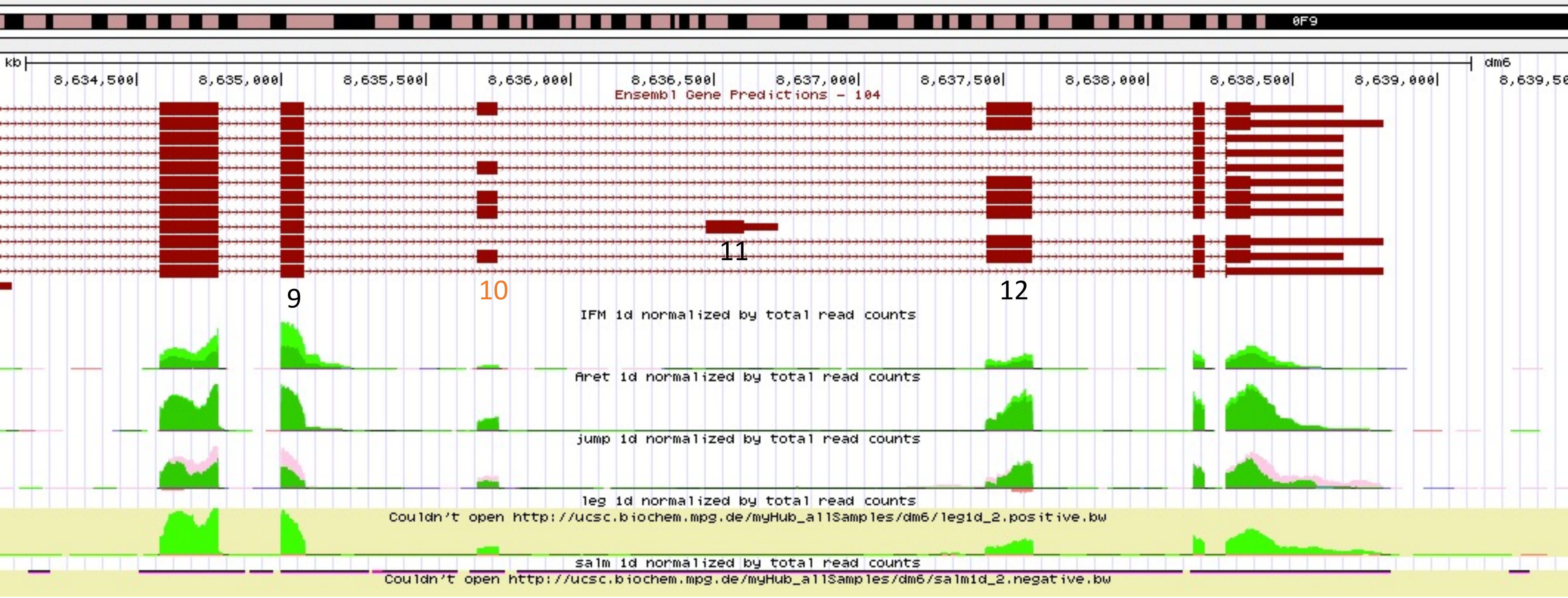
0F9
kb
8,634,500
8,635,000
8,635,500
8,636,000
8,636,500
8,637,000
8,637,500
8,638,000
8,638,500
8,639,000
dm6
Ensembl Gene Predictions - 104
9
10
11
12
IFM 1d normalized by total read counts
Aret 1d normalized by total read counts
jump 1d normalized by total read counts
leg 1d normalized by total read counts
Couldn't open http://ucsc.biochem.mpg.de/myHub_allSamples/dm6/leg1d_2.positive.bw
salm 1d normalized by total read counts
Couldn't open http://ucsc.biochem.mpg.de/myHub_allSamples/dm6/salm1d_2.negative.bw

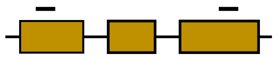

Zasp66_ex10

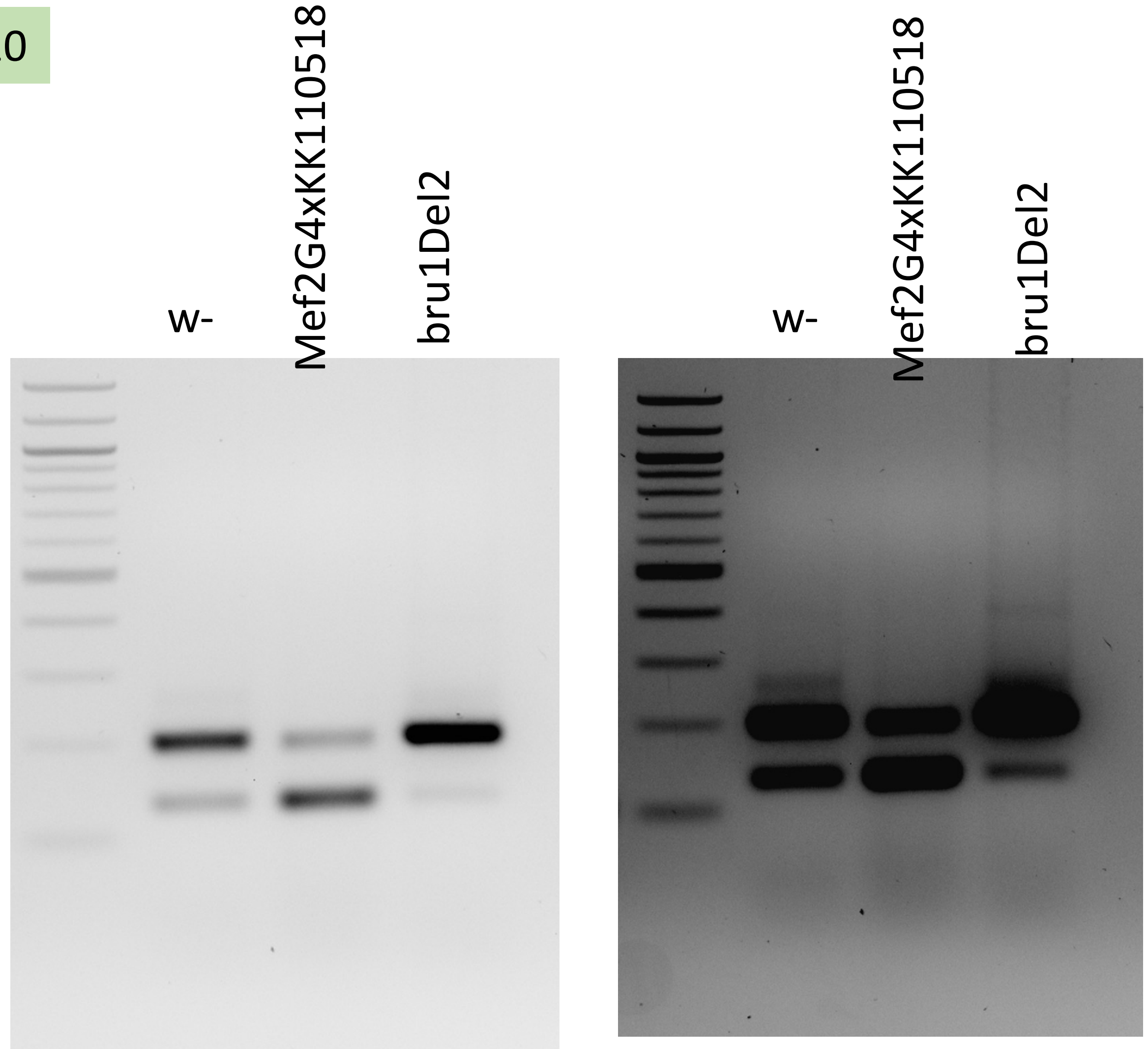


Ex9 F: ATCGAGGACACCATCAGATCC

Ex12 R:ACTTCCTGTGGCGAGGATTGG

Predicted length:

ex9-10-12: 206

Ex9-12: 134

thorax

Zasp66

Ex9 F: ATCGAGGACACCATCAGATCC

Ex12 R:ACTTCCTGTGGCGAGGATTGG

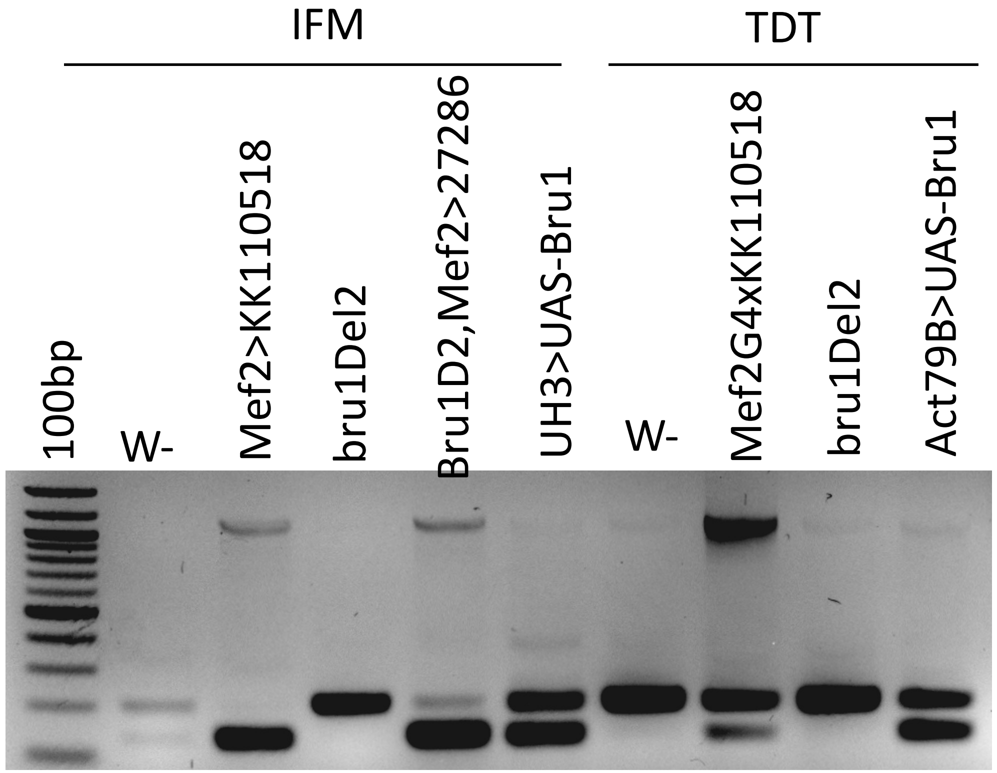


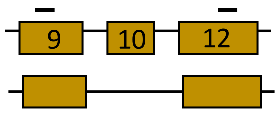


Gel: 211109_R1_zasp66_1

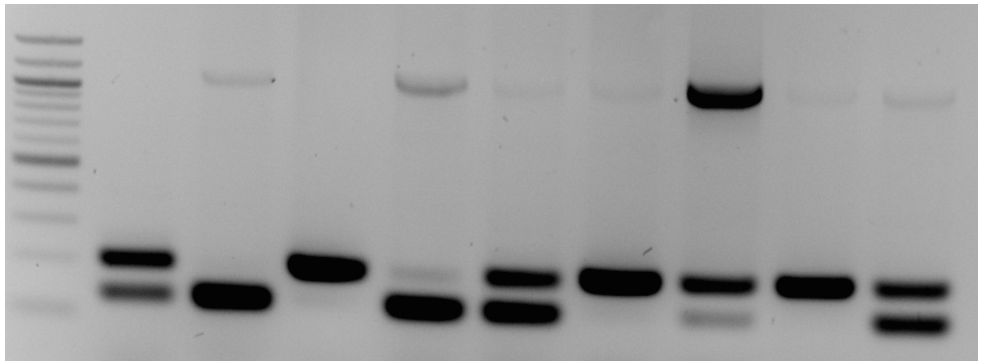

Gel: 211110_R2_zasp66_700ms

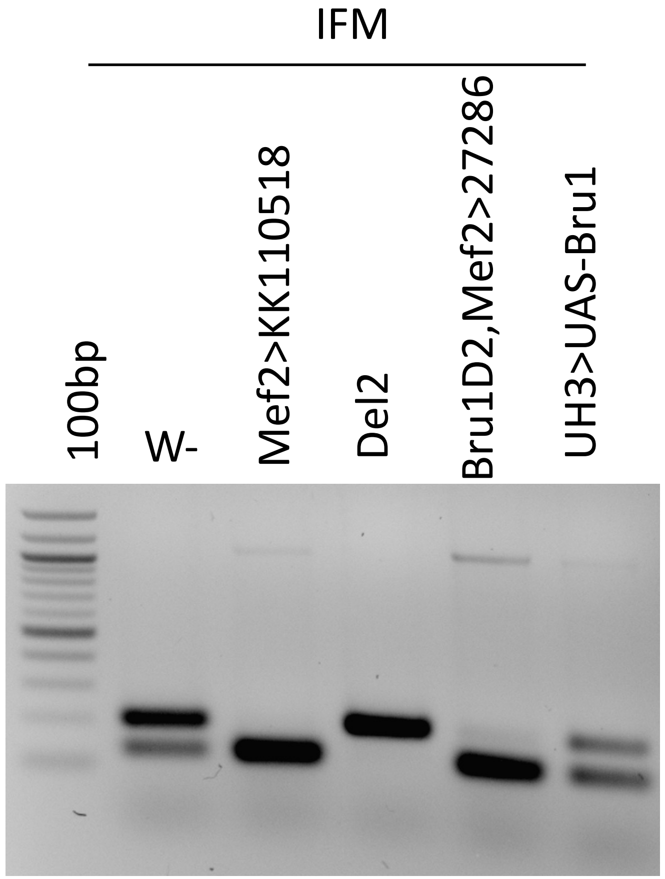


Gel: 211111_R3_zasp66_67_1s

Zasp67

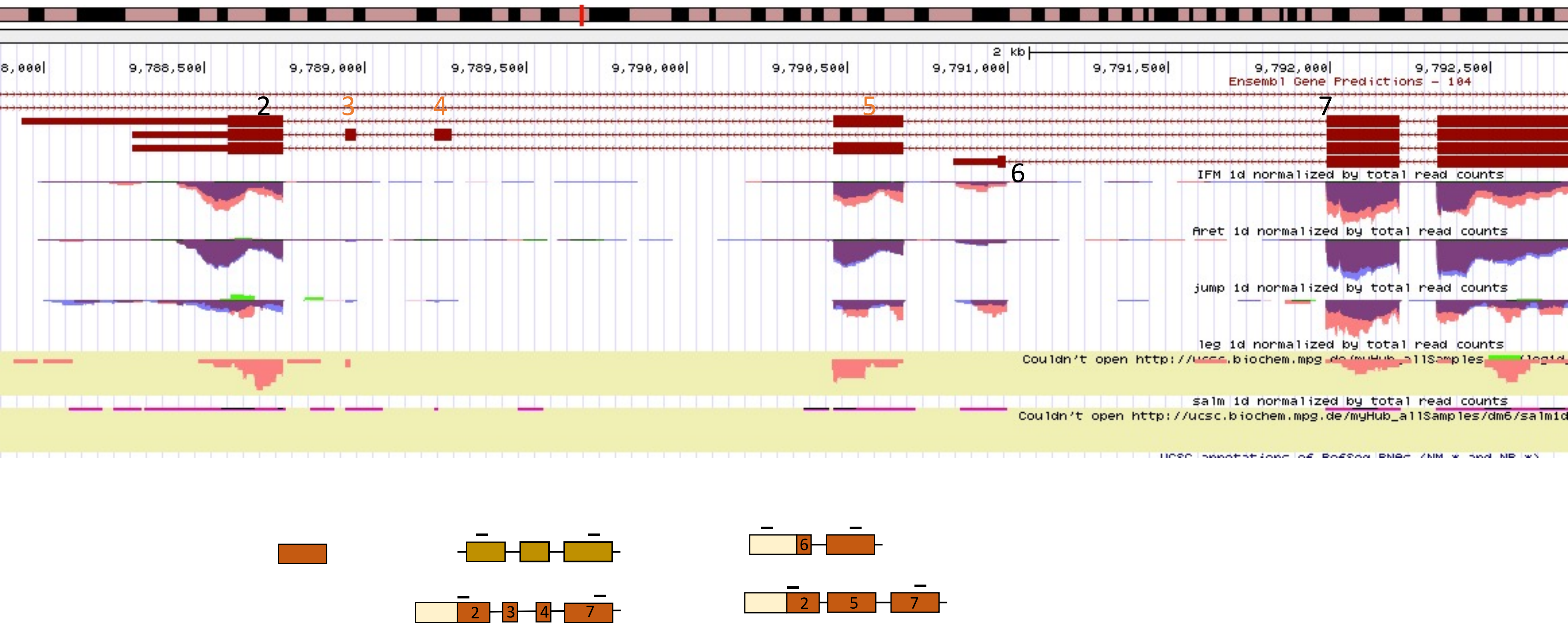

9,788,500
9,789,000
9,789,500
9,790,000
9,790,500
9,791,000
9,791,500
9,792,000
9,792,500
2 kb
Ensembl Gene Predictions - 104
2
3
4
5
7
6
IFM 1d normalized by total read counts
Aret 1d normalized by total read counts
jump 1d normalized by total read counts
leg 1d normalized by total read counts
salm 1d normalized by total read counts
6
2
3
4
7
2
5
7

Zasp67_3UTR

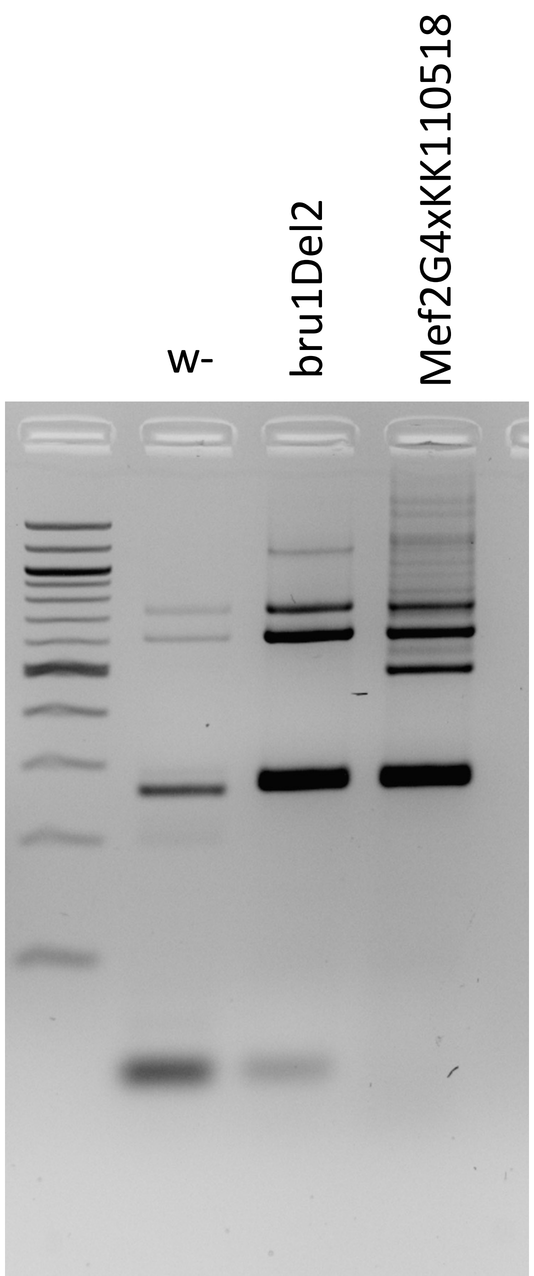


Ex7 F: ATAGATGCGATGCCAATGACCG

Ex2 R: ATGGGATTGACTTGAGATTCGGG

Ex6 R: AGAACCATCTTGGGCACAACGC

Predicted length:

Ex2-3-4-5-7: 710

Ex2-5-7: 621

Ex2-3-4-7: 492

Ex6-7: 268 (serve as control, change not expected)

thorax

Zasp67

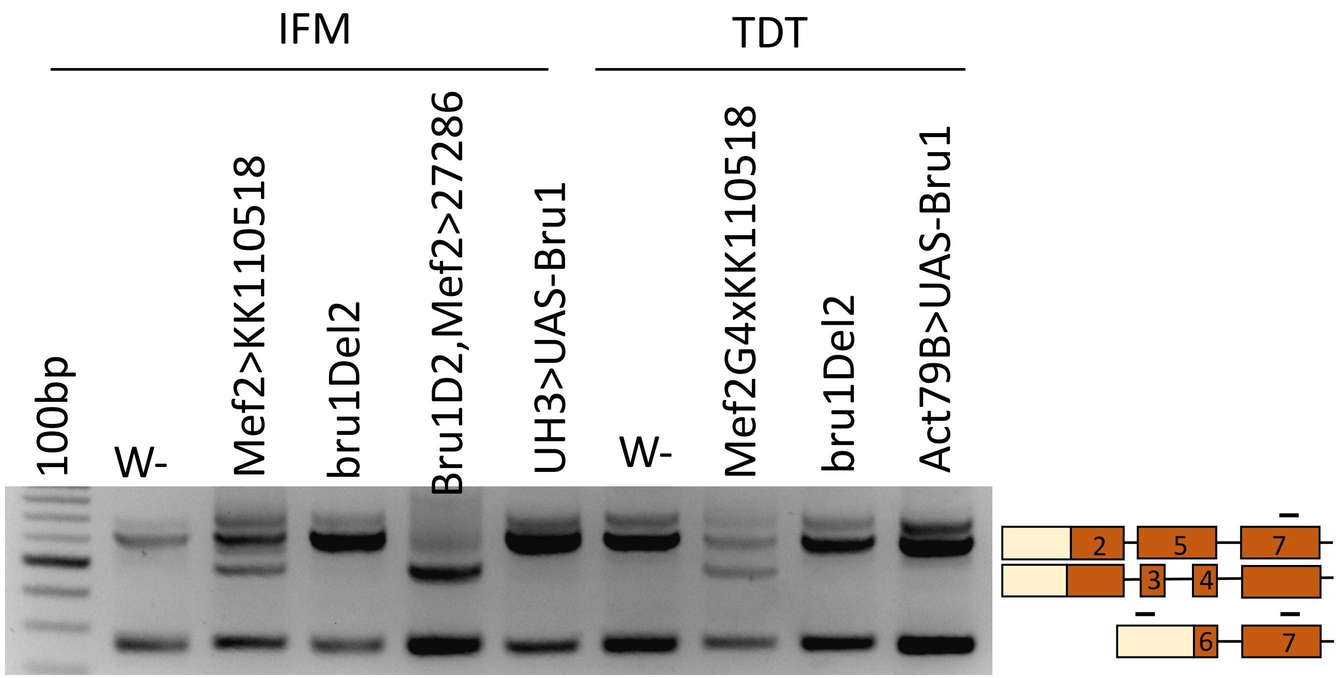


Gel: 211109_R1_zasp67_1.2ms

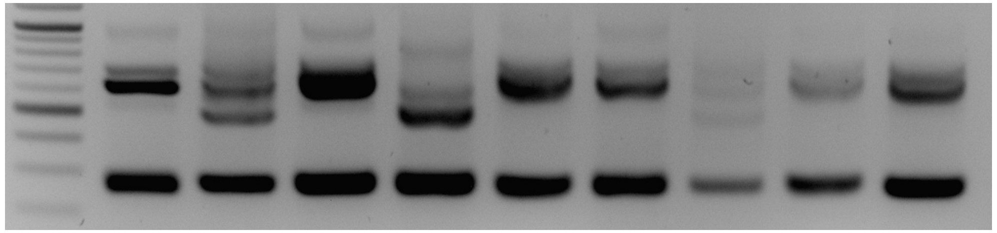

Gel: 211110_R2_zasp67_700ms

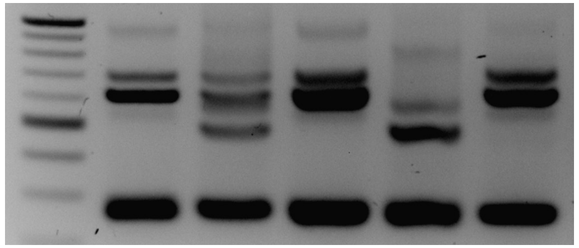

Gel: 211110_R2_zasp67_IFMonly
(Longer run)

Ex7 F: ATAGATGCGATGCCAATGACCG

Ex2 R: ATGGGATTGACTTGAGATTCGGG

Ex6 R: AGAACCATCTTGGGCACAACGC

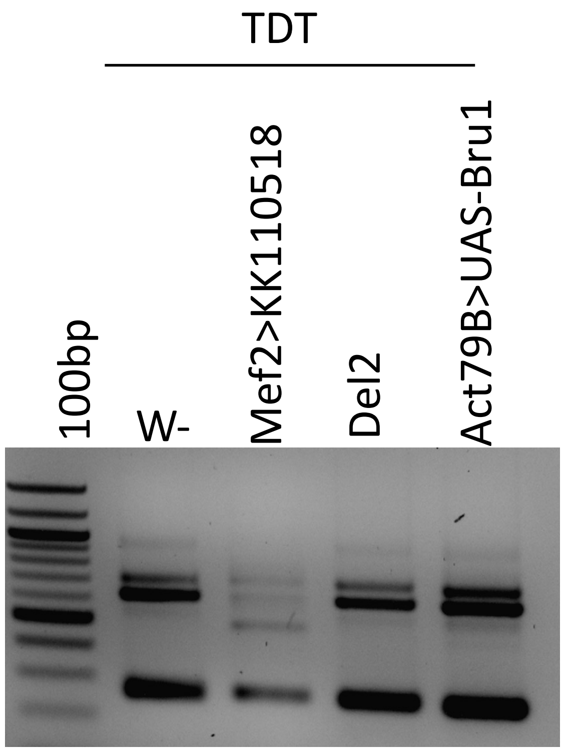


Gel: 211111_R3_zasp66_67_1.3s

# Zasp52

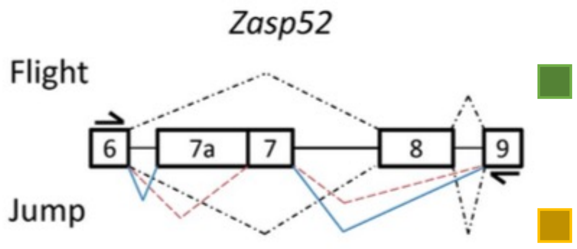


[Oas et al., 2014, JCB]

Primers for Zasp52:
F: ATCGCTTCCGACGTTCTGAAG
R: GTCGCAGTAGAGCTTGTTGTTG
From Oas et al., 2014

Rp49
F: GGTATCgacaacagagtgcg
R: GAACTTCTTGAATCCGGTGGG

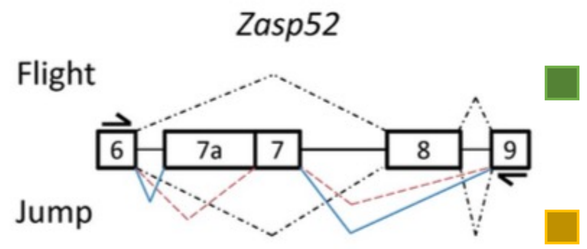


[Oas et al., 2014, JCB]

Primers for Zasp52:
F: ATCGCTTCCGACGTTCTGAAG
R: GTCGCAGTAGAGCTTGTTGTTG
From Oas et al., 2014

Rp49
F: GGTATCgacaacagagtgcg
R: GAACTTCTTGAATCCGGTGGG

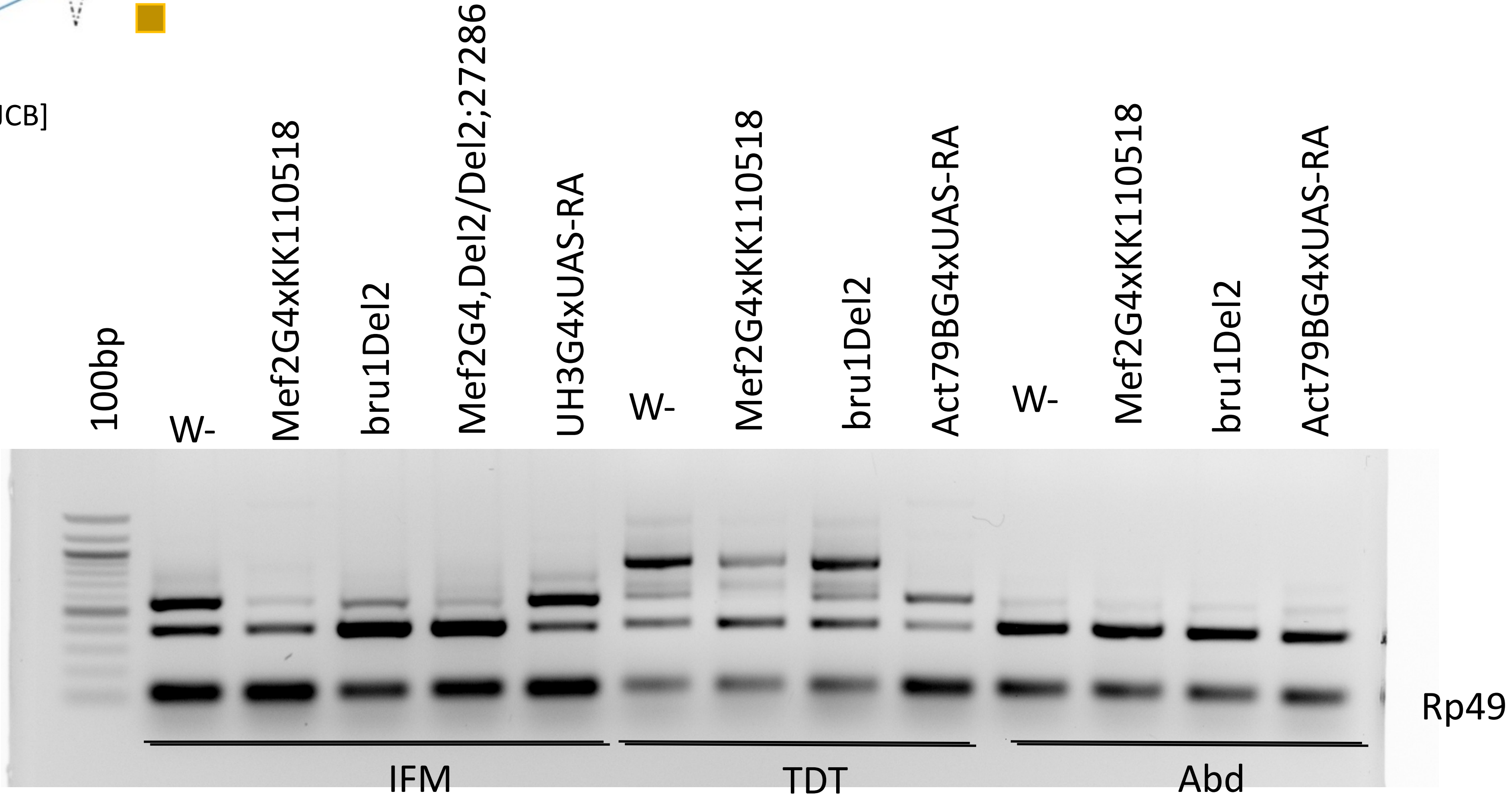


Gel: 201230_Zasp52_AS_700ms

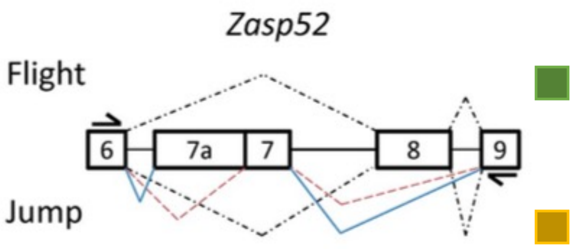


[Oas et al., 2014, JCB]

Primers for Zasp52:
F: ATCGCTTCCGACGTTCTGAAG
R: GTCGCAGTAGAGCTTGTTGTTG
From Oas et al., 2014

Rp49
F: GGTATCgacaacagagtgcg
R: GAACTTCTTGAATCCGGTGGG

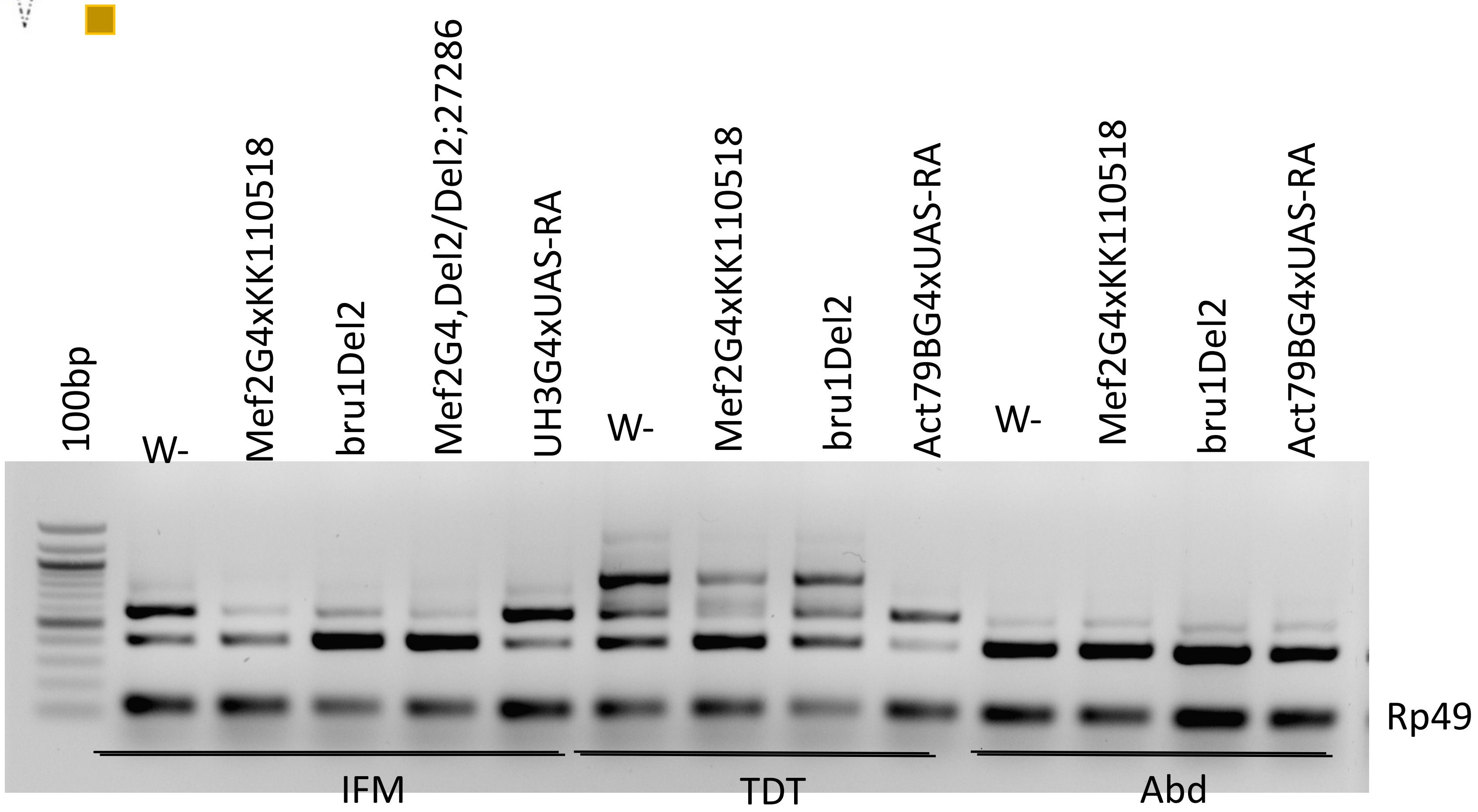


Gel: 210107_1_Zasp52_2
