# Supplementary material for: Rbfox1 is required for myofibril development and maintaining fiber type–specific isoform expression in Drosophila muscles
Source: Life Sci Alliance. 2022 Jan 7;5(4):e202101342. doi: 10.26508/lsa.202101342 (PMC8742874; doi:10.26508/lsa.202101342)
Supplement: Supplementary file 14 [file LSA-2021-01342_SdataFS6.pdf]

Raw data used to generate plots

Figure panel

		band1 (exon9)				band2 (ex9skip)			
	wupA_wt_Ifm	100	100	100	100	0	0	0	0
	wupA_KK110518_IFM	100	100	100	100	0	0	0	0
	wupA_bru1M2_IFM	0	0	0	0	100	100	100	100
	wupA_bru1M2,27286_IFM	0	0	0	0	100	100	100	100
	wupA_UH3bru1_IFM	100	100	100	100	0	0	0	0
**S6T**	wupA_wt_tdt	0	0	0		100	100	100	
	wupA_KK110518_tdt	0	0	0		100	100	100	
	wupA_bru1M2_tdt	0	0	0		100	100	100	
	wupA_Act79BBru1_tdt	100	100	100		0	0	0	
	wupA_wt_abd	3.588469	17.01764	11.59434		96.41153	82.98236	88.40566	
	wupA_KK110518_abd	8.375912	22.37227	14.48378		91.62409	77.62773	85.51622	
	wupA_bru1M2_abd	0	0	0		100	100	100	

		band1 (IFM)		band2 (middle)		band3 (TDT)	
	Mhc_wt_Ifm	83.2332	78.06053	5.008353	8.415093	11.75845	13.52437
	Mhc_KK110518_IFM	25.67999	30.65522	8.453723	12.58222	65.86629	56.76256
	Mhc_bru1M2_IFM	9.029734	14.4357	6.289721	12.14062	84.68054	73.42368
	Mhc_bru1M2,27286_IFM	1.564298	3.266778	2.244635	5.350071	96.19107	91.38315
	Mhc_UH3bru1_IFM	31.43146	30.10756	9.03841	21.13947	59.53013	48.75297
**S6U**	Mhc_wt_tdt	19.69718	24.82017	8.017966	14.61068	72.28485	60.56914
	Mhc_KK110518_tdt	3.72987	5.123226	3.756157	7.19735	92.51397	87.67942
	Mhc_bru1M2_tdt	12.07166	13.99863	6.496314	11.27977	81.43203	74.72159
	Mhc_Act79BBru1_tdt	27.74434	27.84916	9.559719	13.5218	62.69595	58.62904
	Mhc_wt_abd	2.449134	6.244872	1.945428	5.868948	95.60544	87.88618
	Mhc_KK110518_abd	2.999996	4.586192	2.271059	5.109786	94.72895	90.30402
	Mhc_bru1M2_abd	1.093178	1.367763	1.060274	2.695426	97.84655	95.93681

Original RT-PCR gels for alternative splice events

Tropomyosin 1

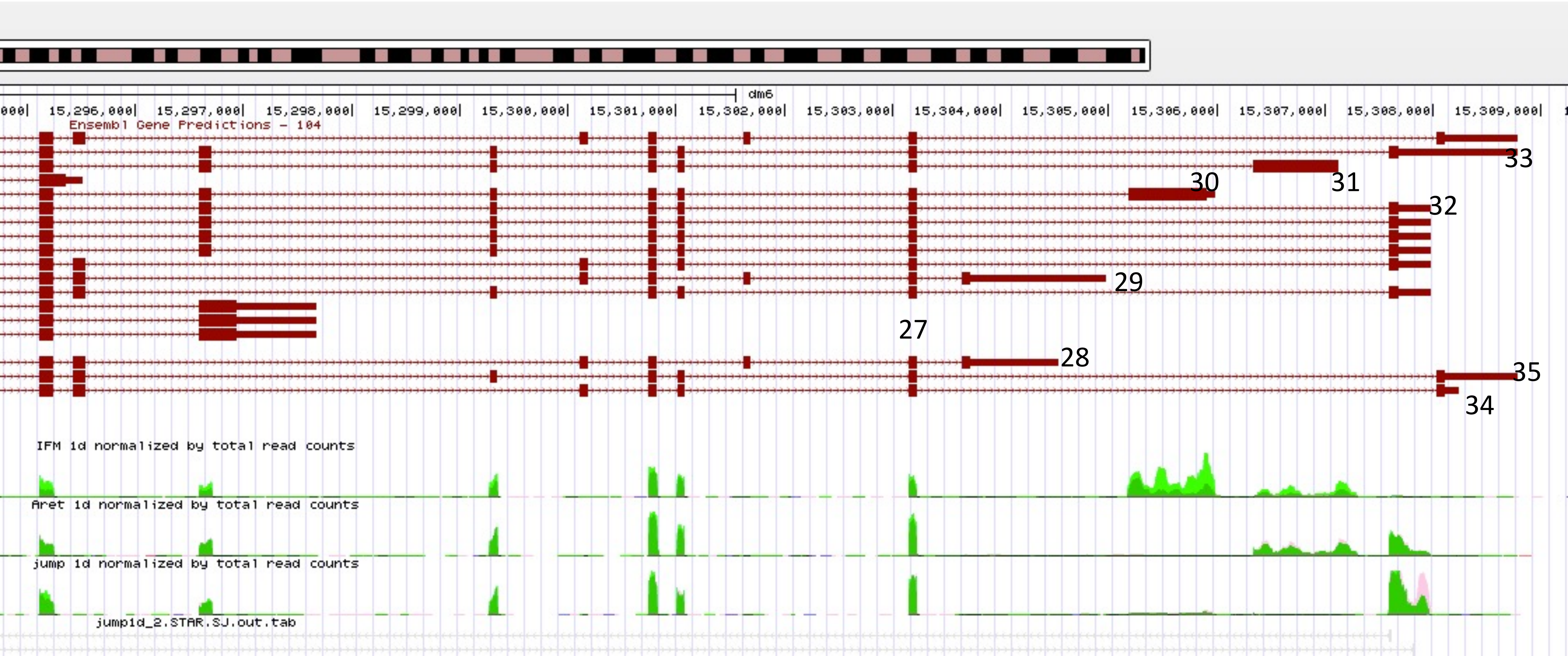

TM1_3UTR

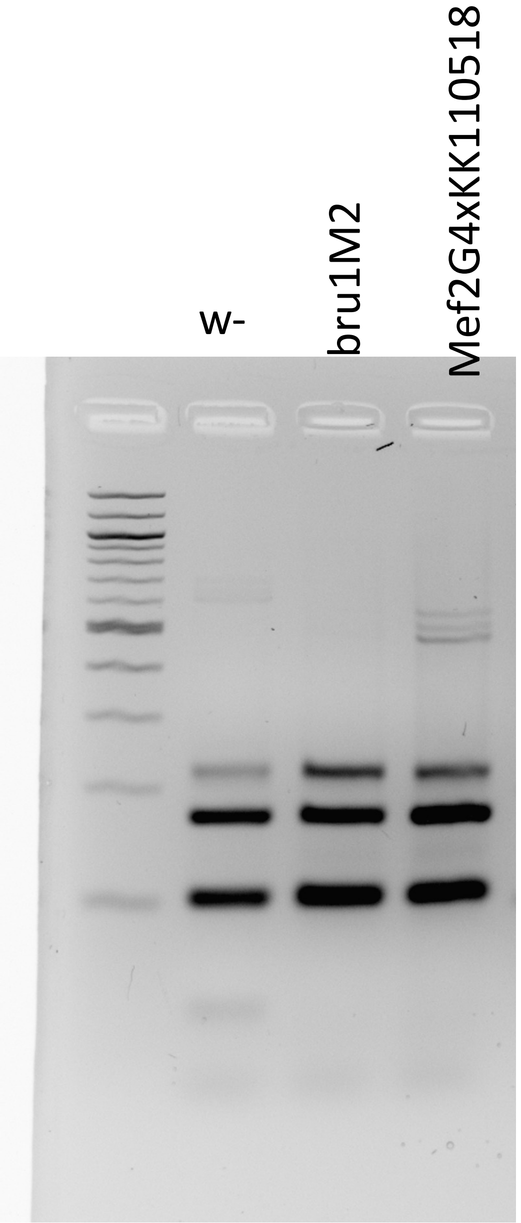

Ex27 F: TGAGTTCGCTGAACGCTCCG

Ex28/29 R: AATCGTGAATTGGAATGCGC

Ex30 R: AGGTGCTGGTGCTCCTTCTGCC

Ex31 R: AGTCGGCGGCTTAGGGTTGCG

Ex32 R: TCGAGATCGTCTCCGATGTCC

Predicted length:

Ex27-28/29: 510

Ex27-30: 625

Ex27-31: 178

Ex27-32/33: 108

thorax

TM1_3UTR_long isos

Ex27 F: TGAGTTCGCTGAACGCTCCG

Ex31 R: AGTCGGCGGCTTAGGGTTGCG

Ex30 R: AGGTGCTGGTGCTCCTTCTGCC

Ex28/29 R: AATCGTGAATTGGAATGCGC

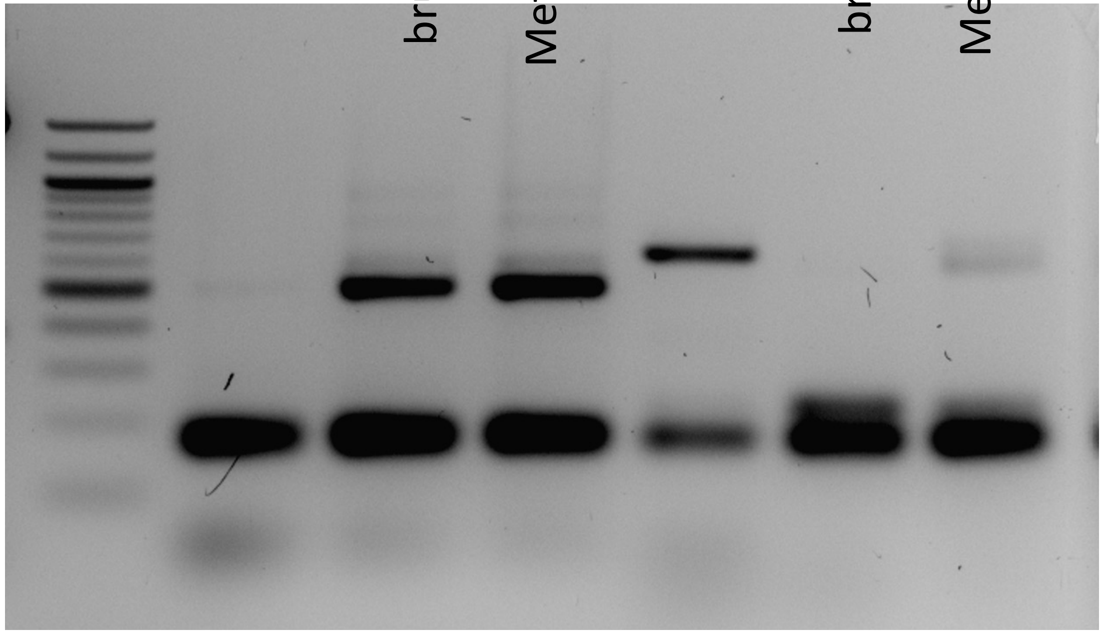

Ex27-31: 178 (as control to all samples)

Ex27-30: 625

Ex27-28/29: 510

Gel:211007_tn_TM1_1s

thorax

rhea

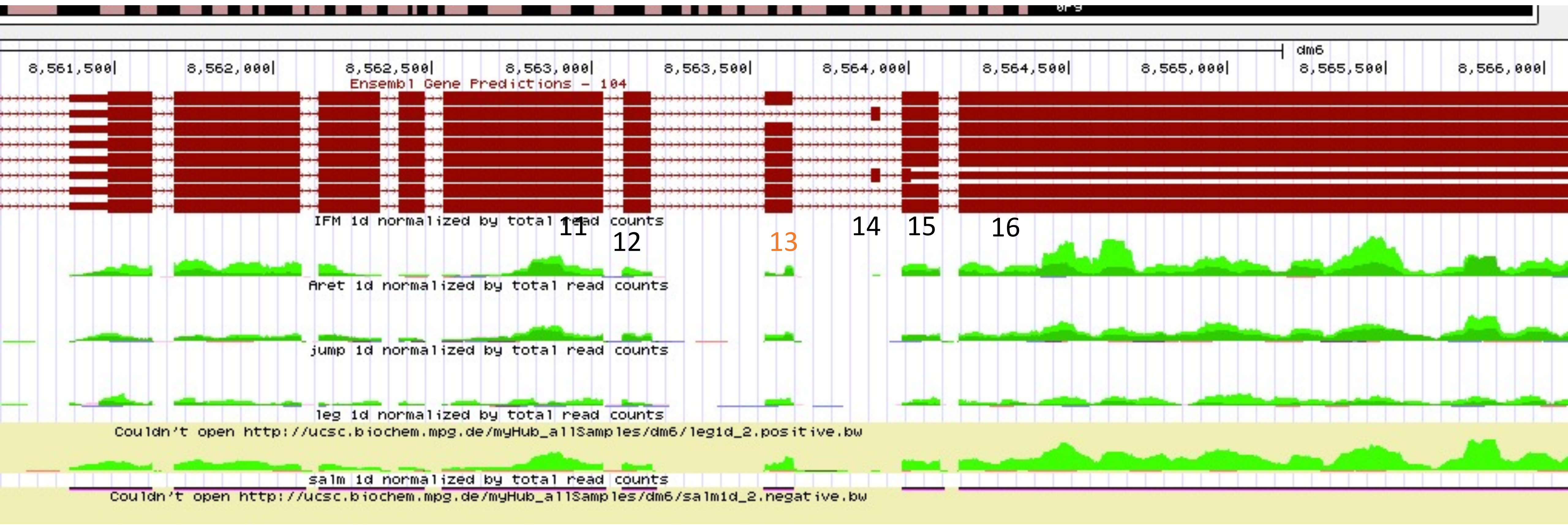

dm6
8,561,500
8,562,000
8,562,500
8,563,000
8,563,500
8,564,000
8,564,500
8,565,000
8,565,500
8,566,000
Ensembl Gene Predictions - 104
IFM 1d normalized by total read counts
11
12
13
14
15
16
Aret 1d normalized by total read counts
jump 1d normalized by total read counts
leg 1d normalized by total read counts
Couldn't open http://ucsc.biochem.mpg.de/myHub_allSamples/dm6/leg1d_2.positive.bw
salm 1d normalized by total read counts
Couldn't open http://ucsc.biochem.mpg.de/myHub_allSamples/dm6/salm1d_2.negative.bw

Rhea_ex13

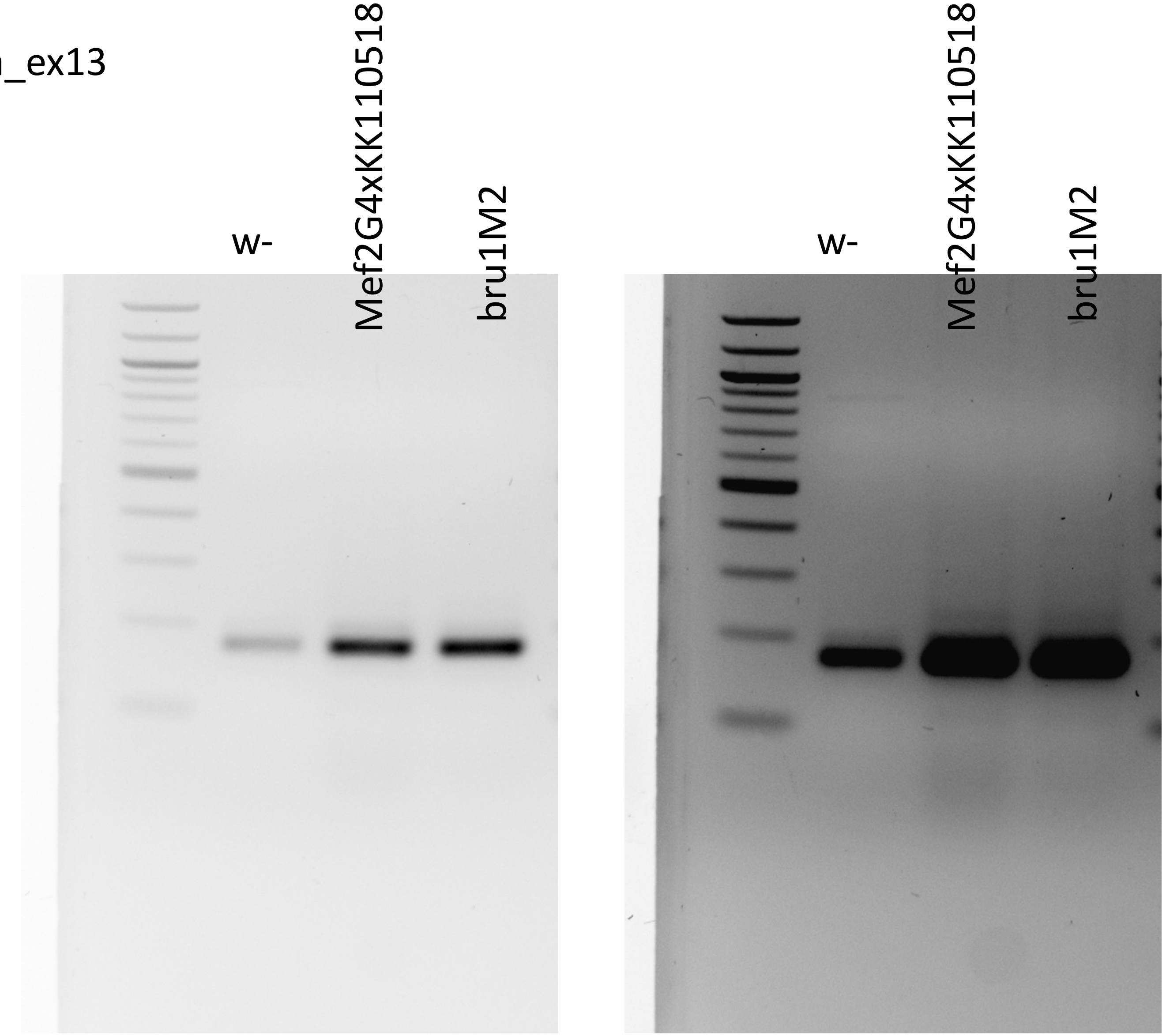

Ex 12 F: CATGGAGCAACTAAATGTGG

Ex15 R: ACAGAACTAATGCGAACTTCC

Predicted length:

ex12-13-14-15: 200

Ex12-13-15: 174

Ex12-14-15: 85

thorax

rhea

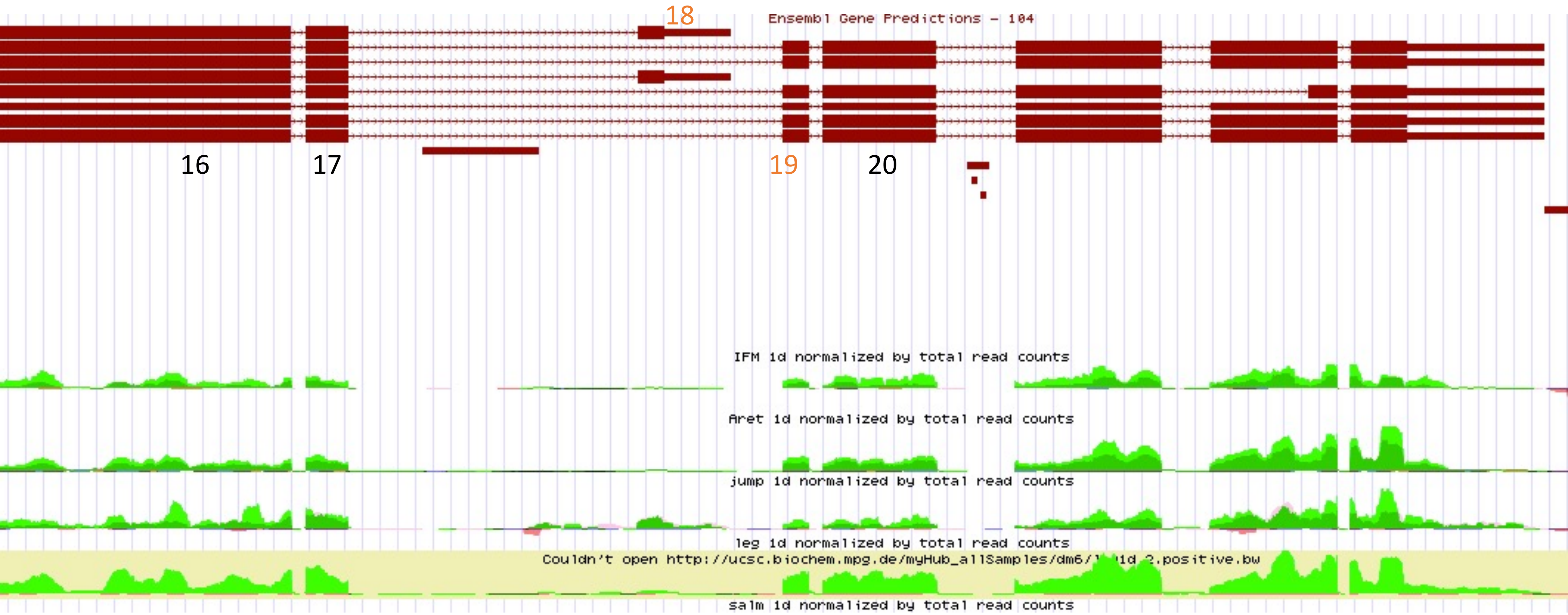

18
Ensembl Gene Predictions - 104
16
17
19
20
IFM 1d normalized by total read counts
Aret 1d normalized by total read counts
jump 1d normalized by total read counts
leg 1d normalized by total read counts
Couldn't open http://ucsc.biochem.mpg.de/myHub_allSamples/dm6/
salm 1d normalized by total read counts

Rhea_3UTR

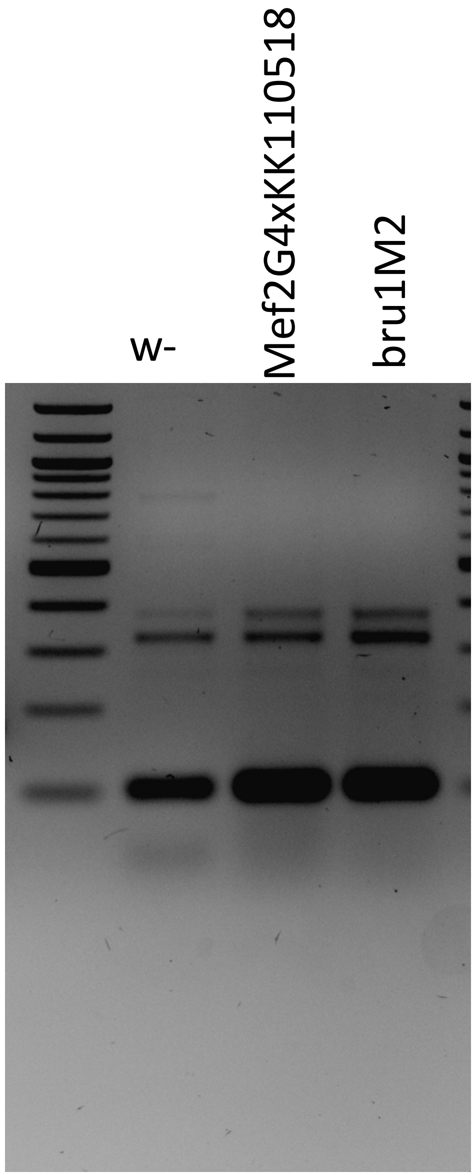

Ex17 F: GATTAACTGCACTAAGTTGGC

Ex18 R: ATTCTCATTGCTCTTCCCAGC

Ex19 R: GTAGTGAGGACACATTCAGC

Predicted length:

Ex17-18: 328

Ex17-19: 104

thorax

tn

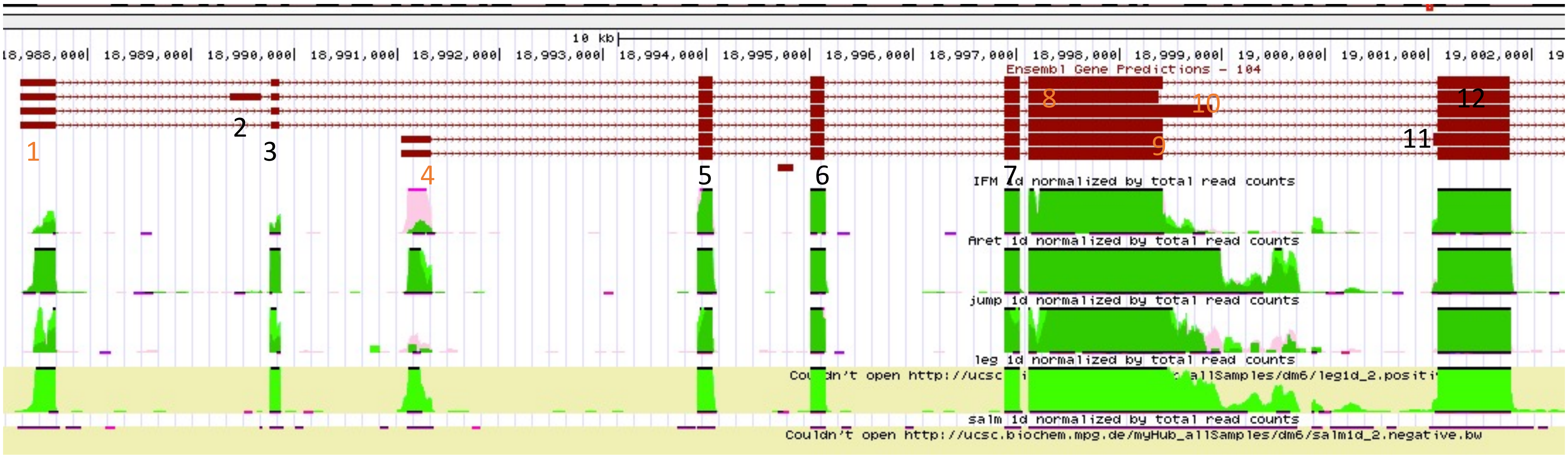
10 kb
18,988,000 18,989,000 18,990,000 18,991,000 18,992,000 18,993,000 18,994,000 18,995,000 18,996,000 18,997,000 18,998,000 18,999,000 19,000,000 19,001,000 19,002,000
Ensembl Gene Predictions - 104
1
2
3
4
5
6
7
8
9
10
11
12
IFM 1d normalized by total read counts
Aret 1d normalized by total read counts
jump 1d normalized by total read counts
leg 1d normalized by total read counts
salm 1d normalized by total read counts
Couldn't open http://ucsc.biochem.mpg.de/myHub_allSamples/dm6/salm1d_2.negative.bw

tn_5UTR

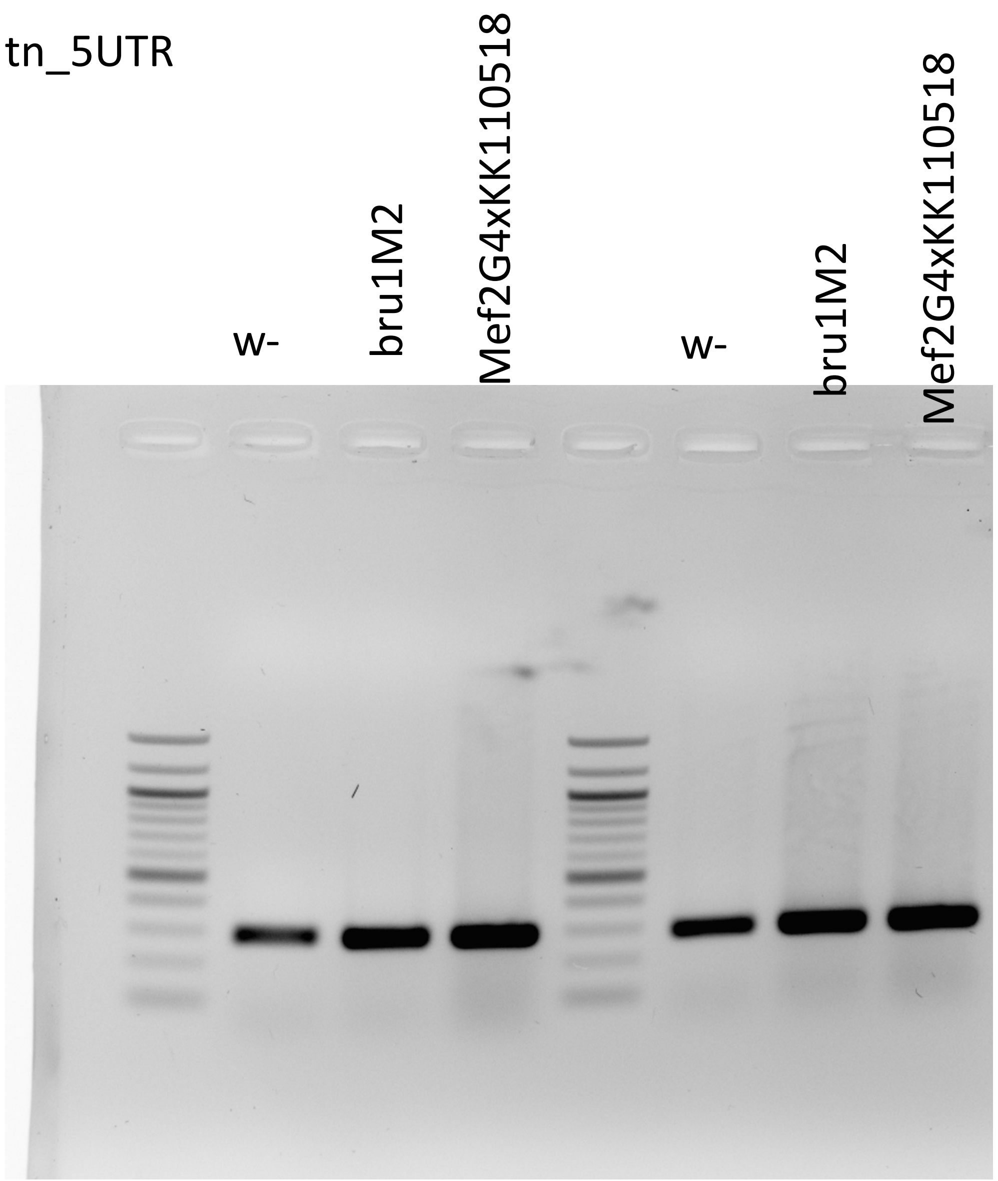

Ex1_F: AGGCGGTAATACATCGTACC

Ex5 R: AGCTTGGGAATCCTGTACC

Ex4 F: AATCTAAACGACACCGTACCG

Ex5 R: AGCTTGGGAATCCTGTACC

Predicted length:

Ex1-2-3-5: 591

Ex1-2-5: 286

Ex4-5: 290

thorax

tn ex8-10

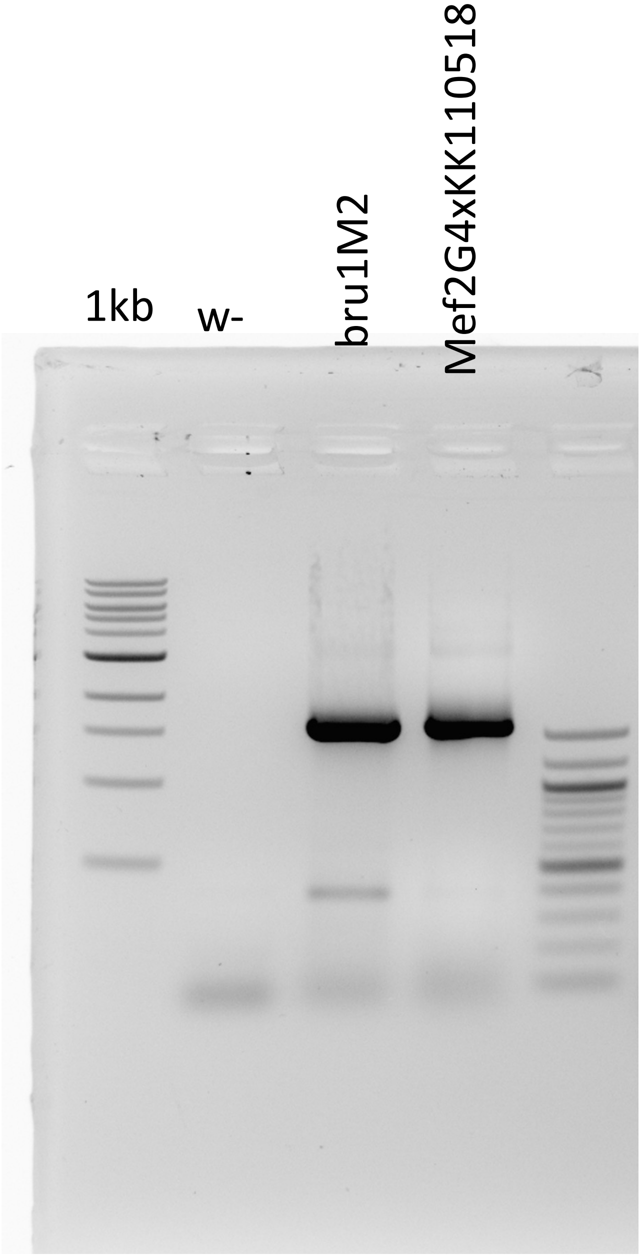

Ex7 F: AGCCTATCTGTCCCATTGTGC

Ex12 R: TGGCTTGTCGAATTGCTGCTGG

Predicted length:

Ex7-8-11: 1599
Ex7-8-12: 1548

Ex7-9-11: 1635
Ex7-9-12: 1584

Ex7-10-11: 2121
Ex7-10-12: 2070

Ex7-11: 400

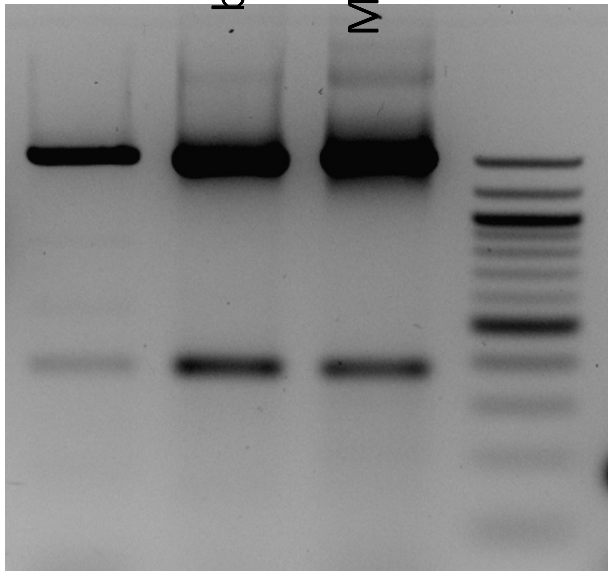

Gel:211007_tn_TM1_1s

thorax

tn

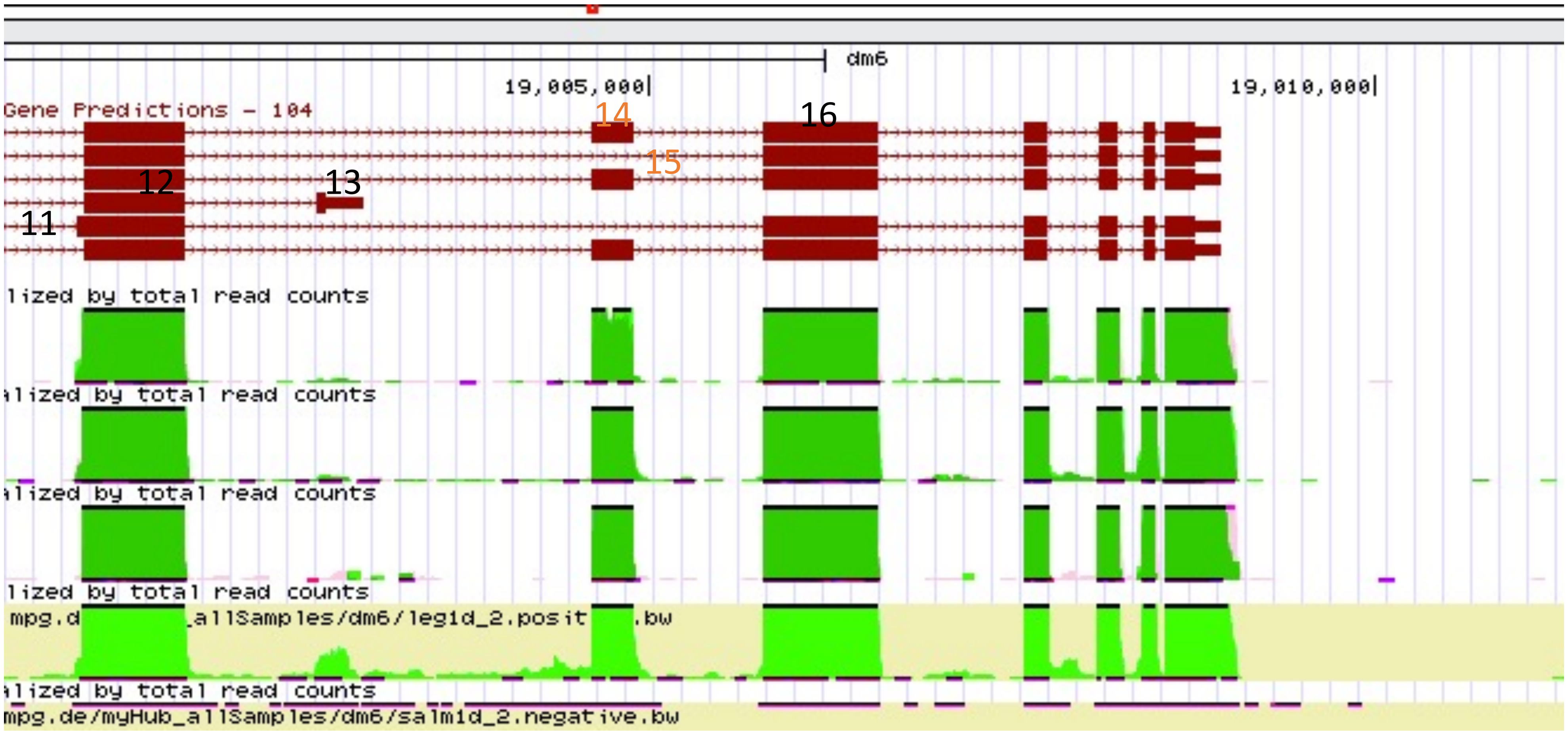
dm6
19,005,000
19,010,000
Gene Predictions - 104
14
16
15
12
13
11
lized by total read counts
alized by total read counts
alized by total read counts
lized by total read counts
mpg.d _allSamples/dm6/legid_2.posit .bw
alized by total read counts
mpg.de/myHub_allSamples/dm6/salmid_2.negative.bw

Tn_ ex14

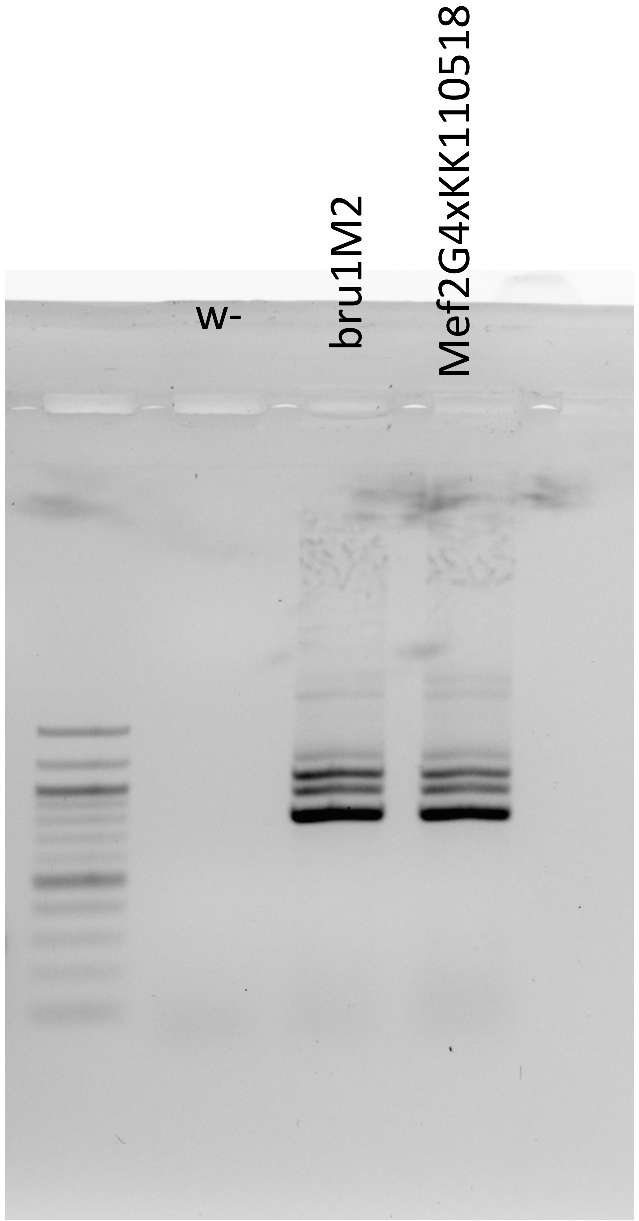

Ex12 F: ACCAGCAGCACCACCAATACC

Ex16 R: ACCTGTTTAGGGATAGTACCTGCC

Predicted length:

Ex12-14/15-16: 1088

Ex12-16: 809

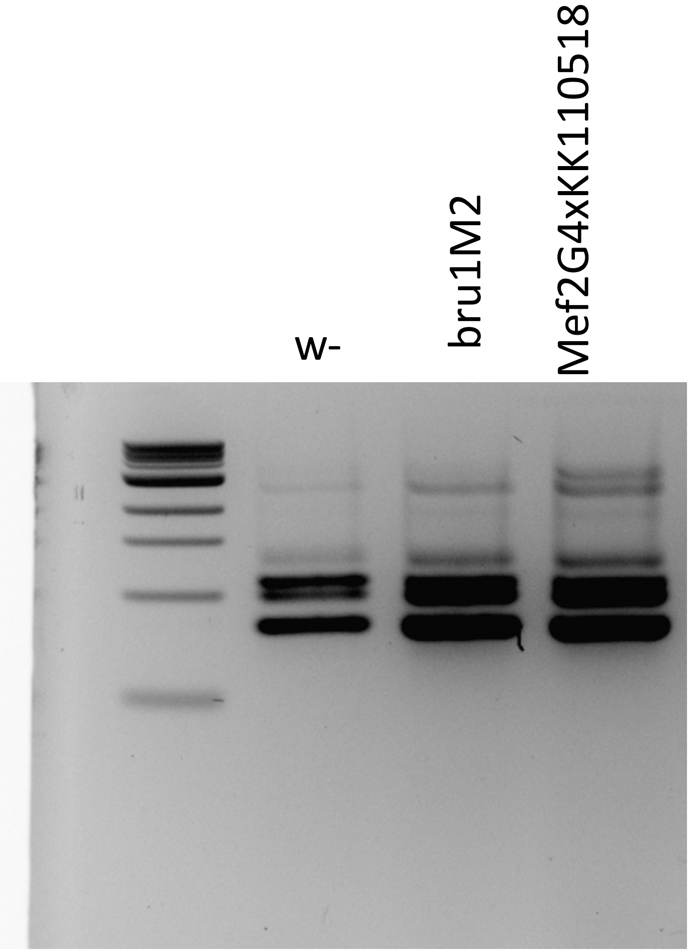

Gel:211007_tn_TM1_1s

thorax

Fhos

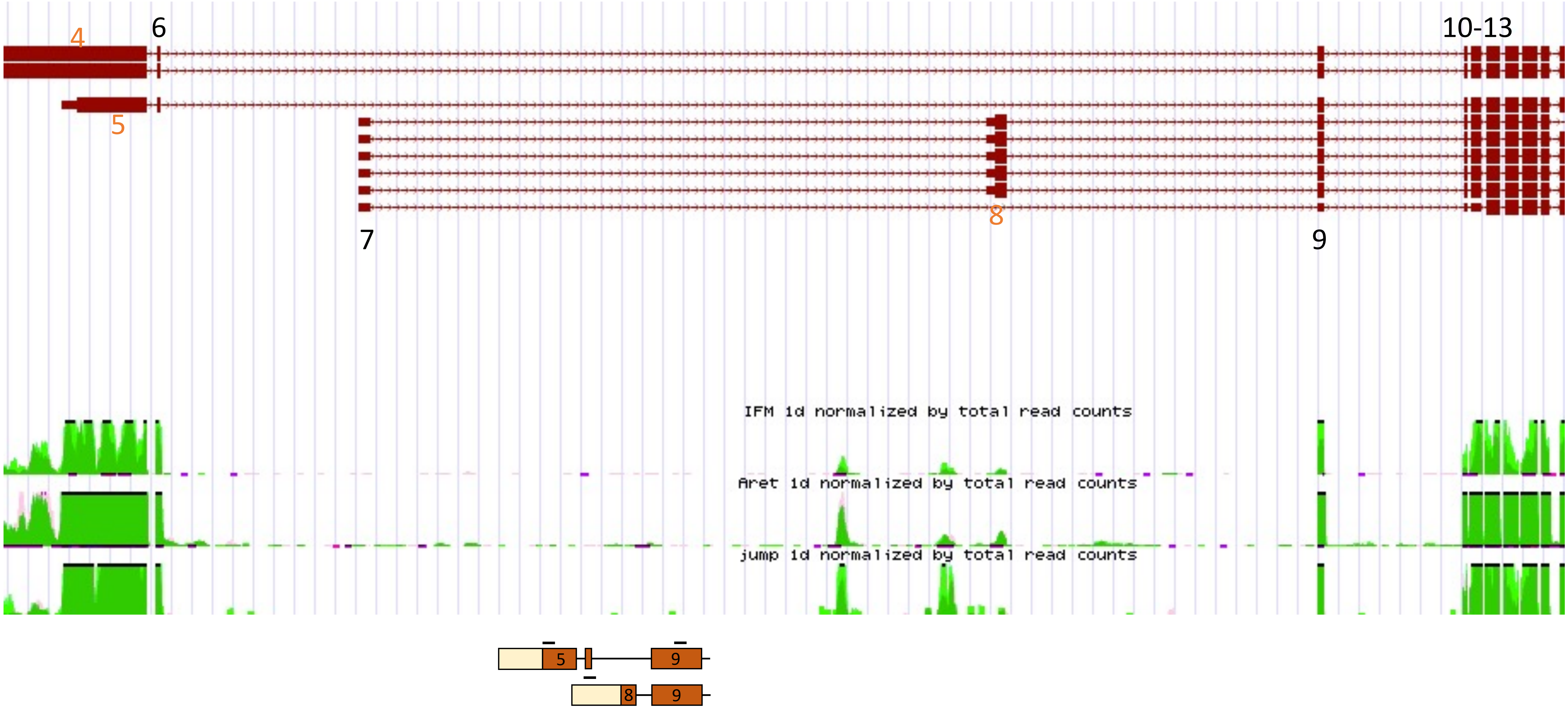
4
6
10-13
5
8
7
9
IFM 1d normalized by total read counts
Aret 1d normalized by total read counts
jump 1d normalized by total read counts
5
9
8
9

Fhos_5UTR

Ex4/5 F:CTCGCCCTACGACAACATTCC

Ex8_F: ATGATTGTGAAAATGGAGCC

Ex9 R: ATGACTCCAGATCCAAGTAGG

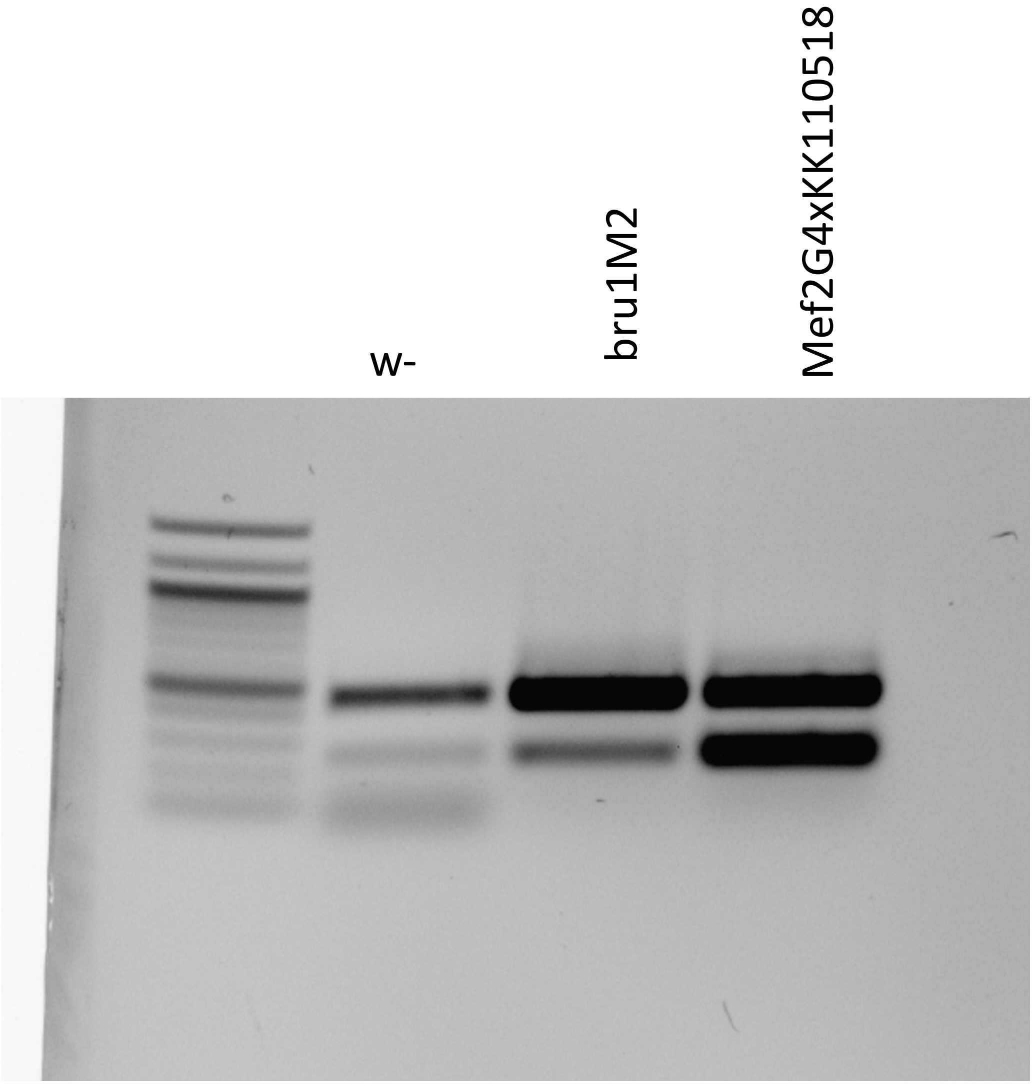

Predicted length:

Ex4/5-6-9: 473

Ex8-9: 256

thorax

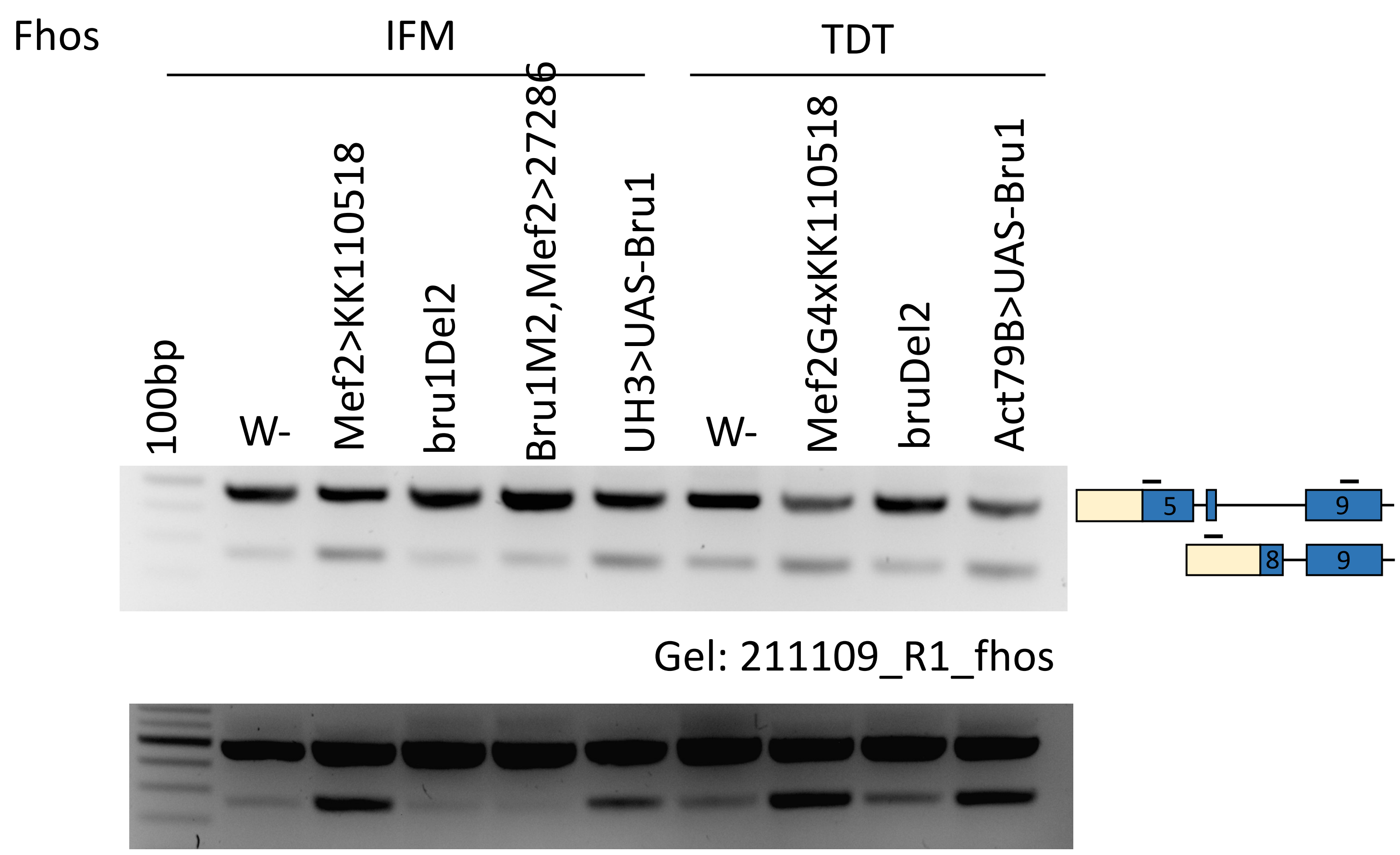

Gel: 211109_R1_fhos

Gel: 211110_R2_fhos_1s

Ex4/5 F:CTCGCCCTACGACAACATTCC

Ex8_F: ATGATTGTGAAAATGGAGCC

Ex9 R: ATGACTCCAGATCCAAGTAGG

sals

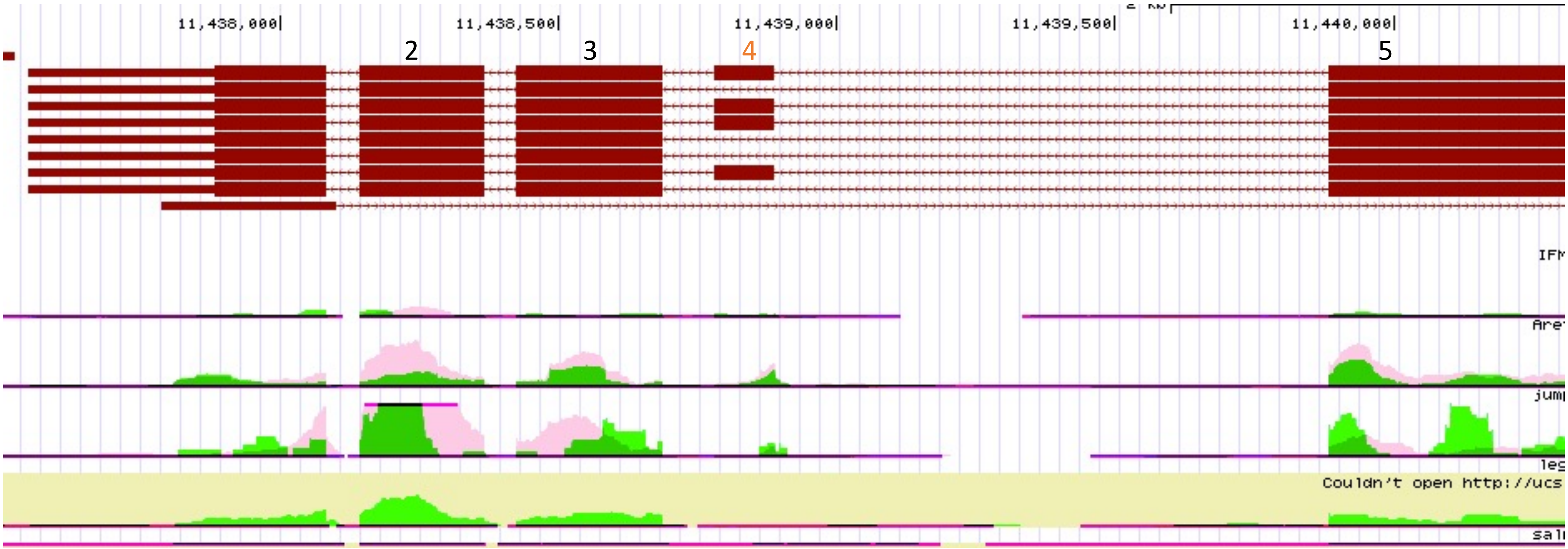

11,438,000
11,438,500
11,439,000
11,439,500
11,440,000
2
3
4
5
IFM
Are
jum
leg
Couldn't open http://ucs
sal

Sals_ex4

w- | bru1M2 | Mef2G4xKK110518

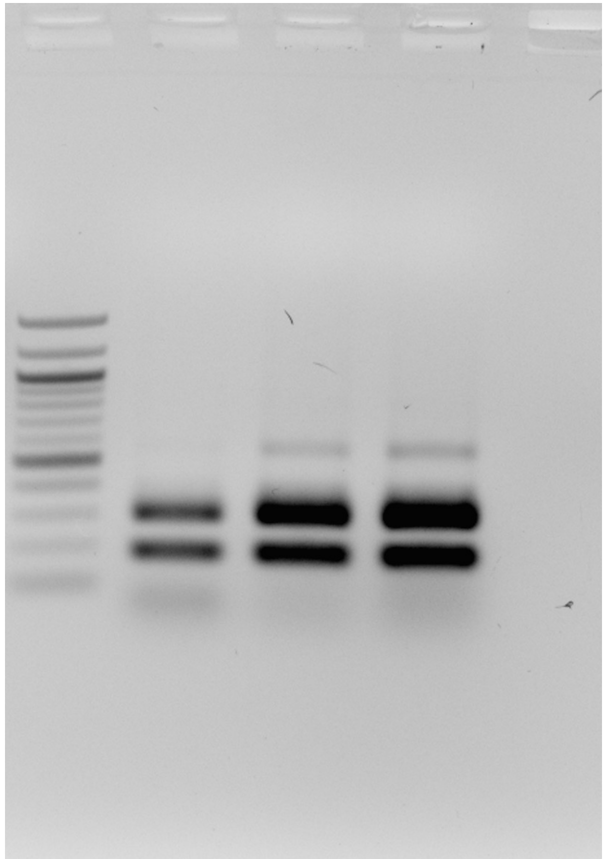

Ex5 F: CTATCAGAGCACCGCCGAGC

Ex3 R: CTGACCTCAGGCTCCAGTTCC

Predicted length:

Ex3-4-5: 297

Ex3-5: 189

thorax

Mhc

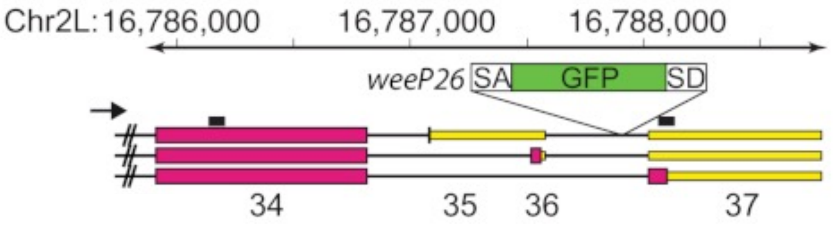

[Kao, Nikonova,..,Spletter, 2019, JoVE]

Mhc:
F: GACGAACTCCTGAACGAAGC
R: TCAGGAGCAAGGTCGAATCT

From Orfanos et al., 2012

Rp49
F: GGTATCgacaacagagtgcg
R: GAACTTCTTGAATCCGGTGGG

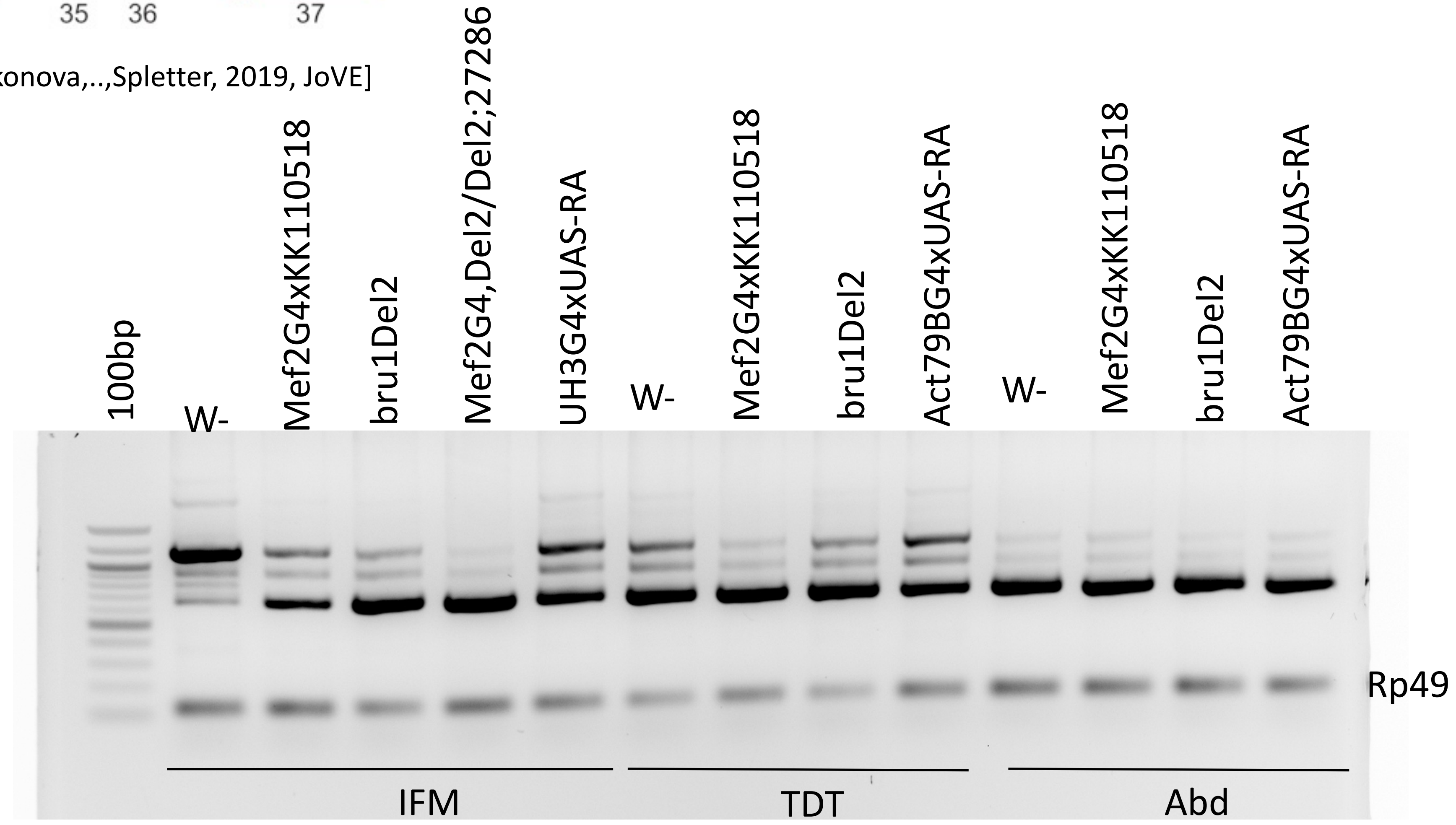

Gel_201230_Mhc_AS_520ms

Mhc

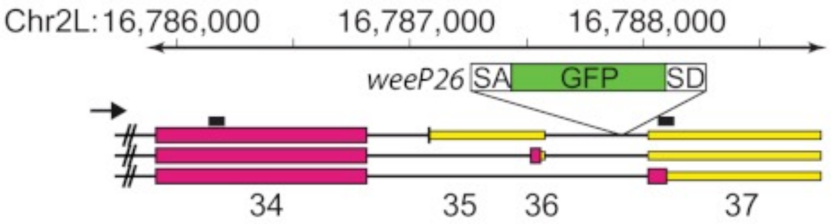

[Kao, Nikonova,..,Spletter, 2019, JoVE]

Mhc:
F: GACGAACTCCTGAACGAAGC
R: TCAGGAGCAAGGTCGAATCT

From Orfanos et al., 2012

Rp49
F: GGTATCgacaacagagtgcg
R: GAACTTCTTGAATCCGGTGGG

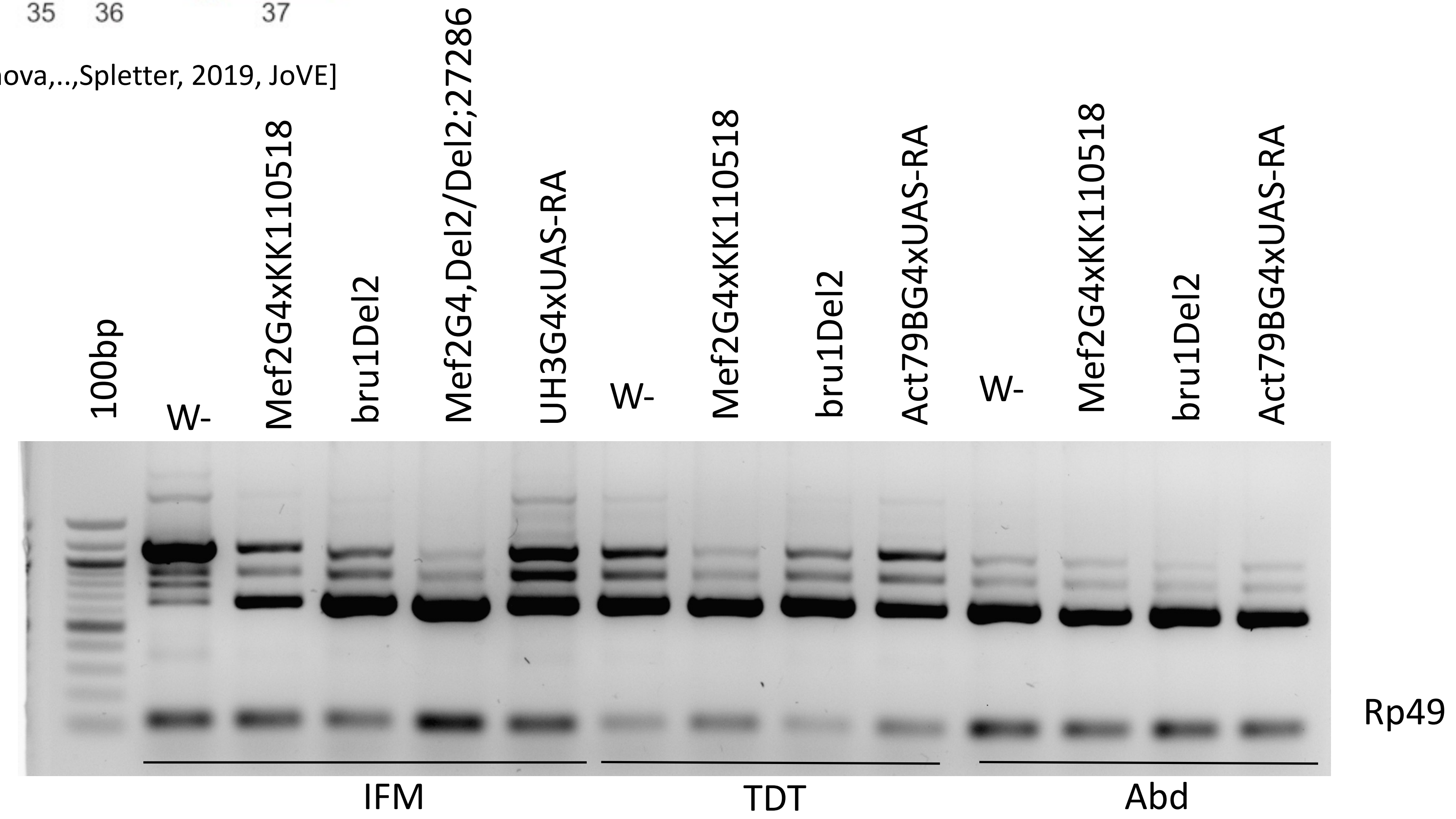

Gel_210107_2_Mhc_2

wupA

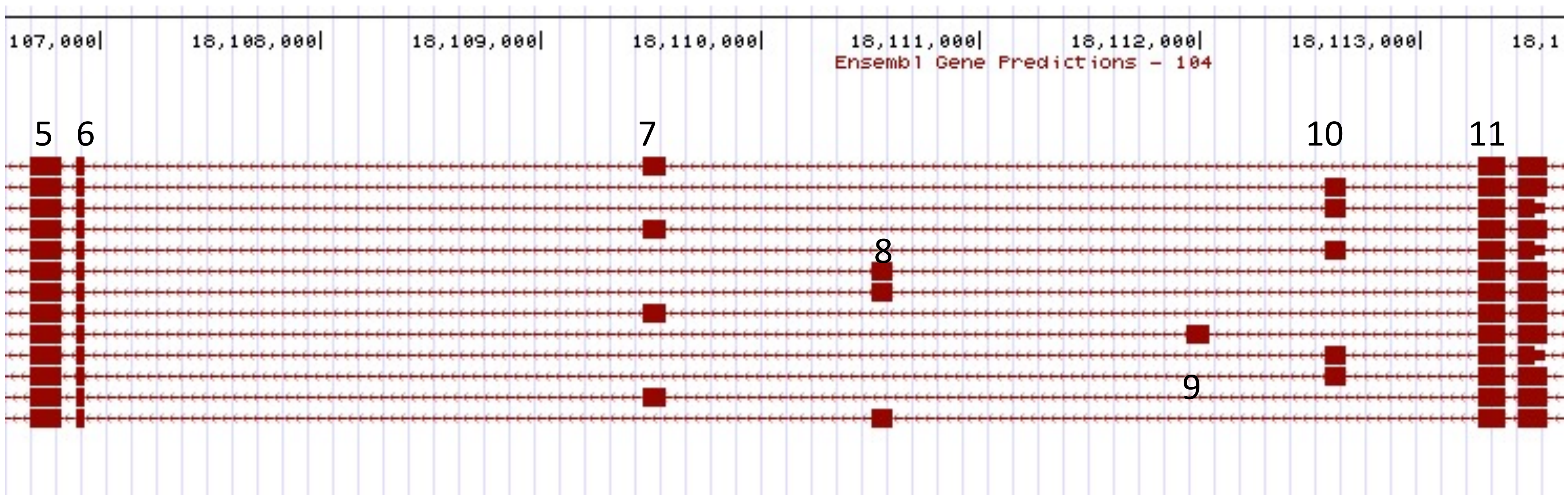

Based on Sanger sequencing of RT-PCR bands:

in both IFM and TDT of W- and M2 the exon 7 is included

in both IFM and TDT of Mef2G4x266 exon 7 is included

wupA (TnI) ex3- ex8

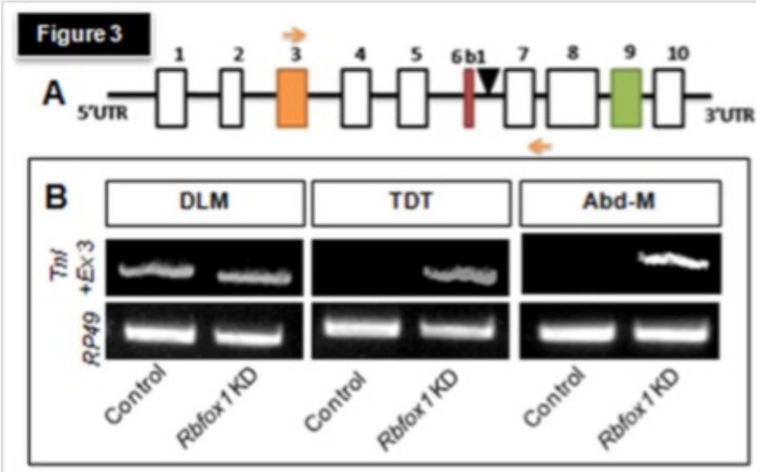

Primers:
TnI
F: CAACGACCCCAAAGTGAAGG
R: TTTAGCACAGCGGCATCTTT
Rp49
F: GGTATCgacaacagagtgcg
R: GAACTTCTTGAATCCGGTGGG

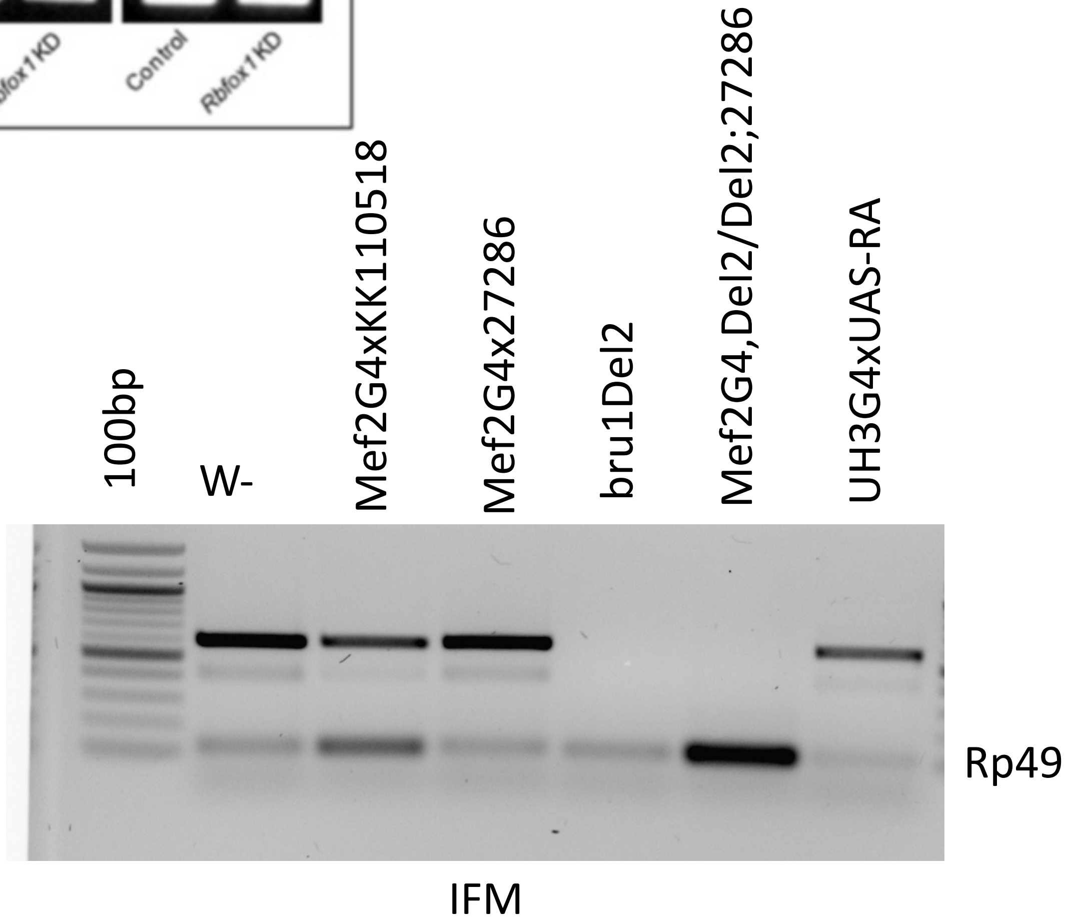

Gel: 201223_TnI_IFM///

wupA (TnI) ex3- ex8

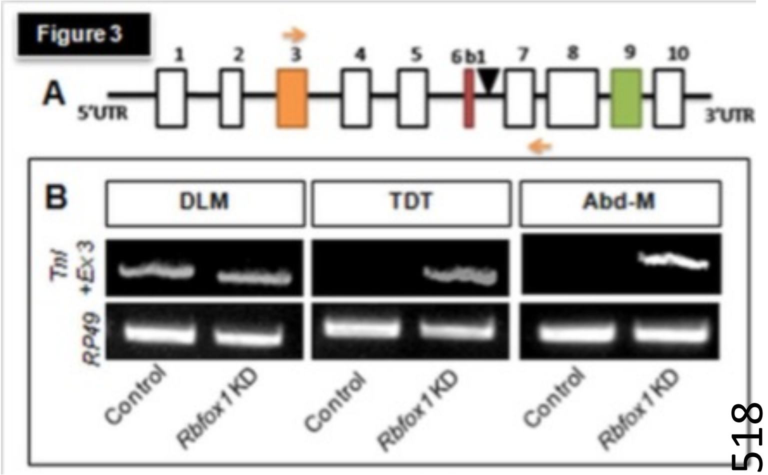

Primers:

TnI

F: CAACGACCCCAAAGTGAAGG

R: TTTAGCACAGCGGCATCTTT

Rp49

F: GGTATCgacaacagagtgcg

R: GAACTTCTTGAATCCGGTGGG

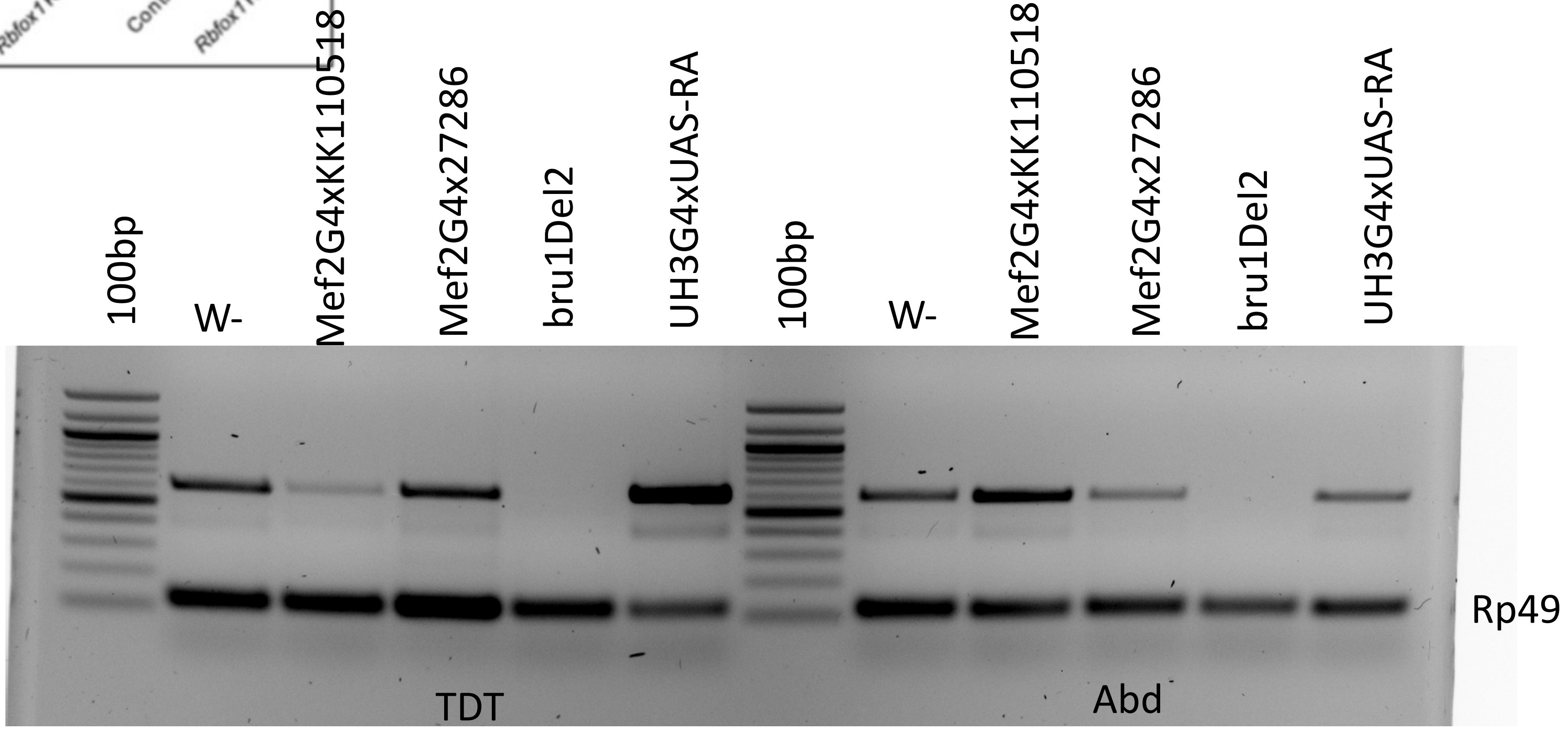

Gel: 201223_TnI_R_TDT_L_Abd_1.1sec

WupA ex8

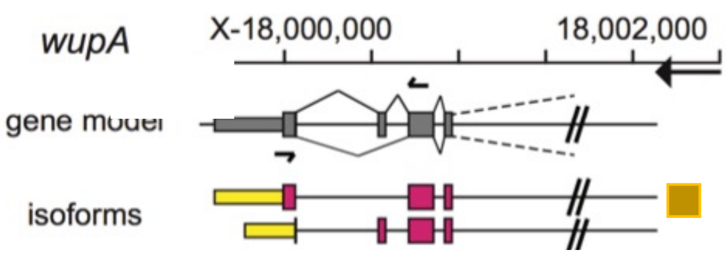

[Nikonova, Kao,..,Spletter, 2019, IJBCB]

Primers:
wupA
F: CGCTGAGTTCAACTTCCGCAACC
R: ATTGTTTAGGGCGGGAGTCACGG

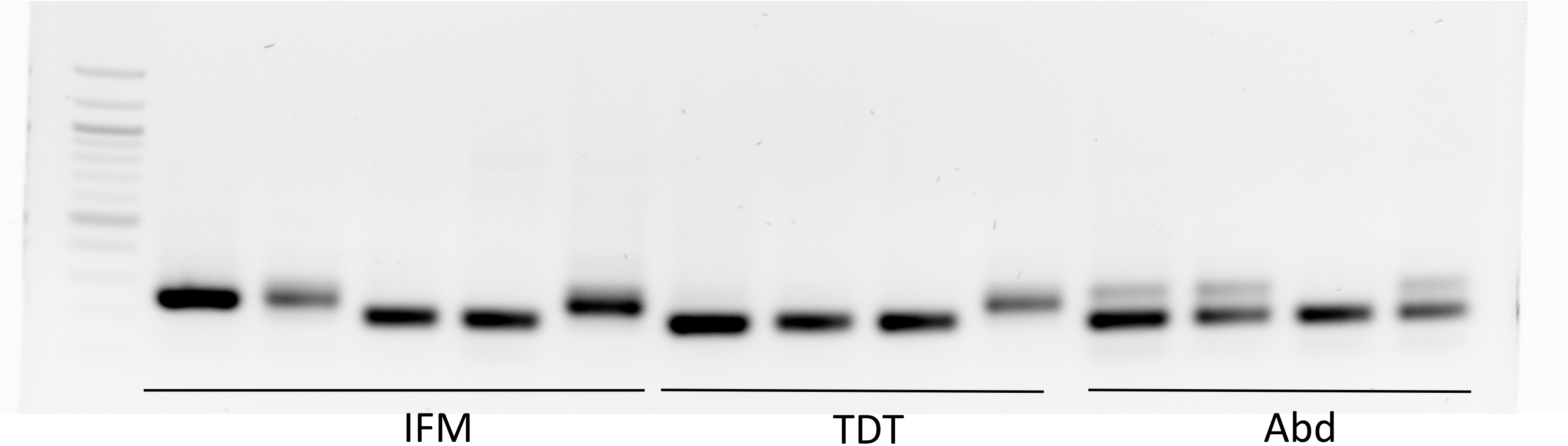

201229_wupA_AS_40min.run

WupA ex8

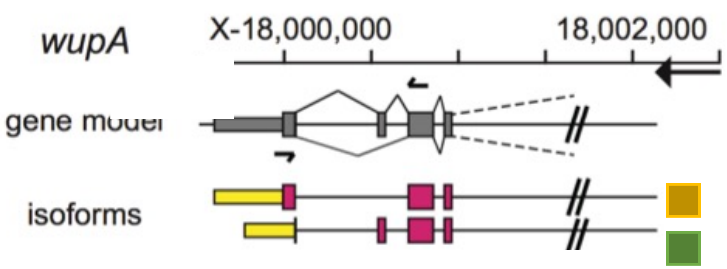

[Nikonova, Kao,..,Spletter, 2019, IJBCB]

Primers:
wupA
F: CGCTGAGTTCAACTTCCGCAACC
R: ATTGTTTAGGGCGGGAGTCACGG

Rp49 (at 500bp)
F: TCCTACCAGCTTCAAGATGAC
R: GTGTATTCCGACCACGTTACA
Seq from Orfanos et al. 2012

RP49 didn't worked

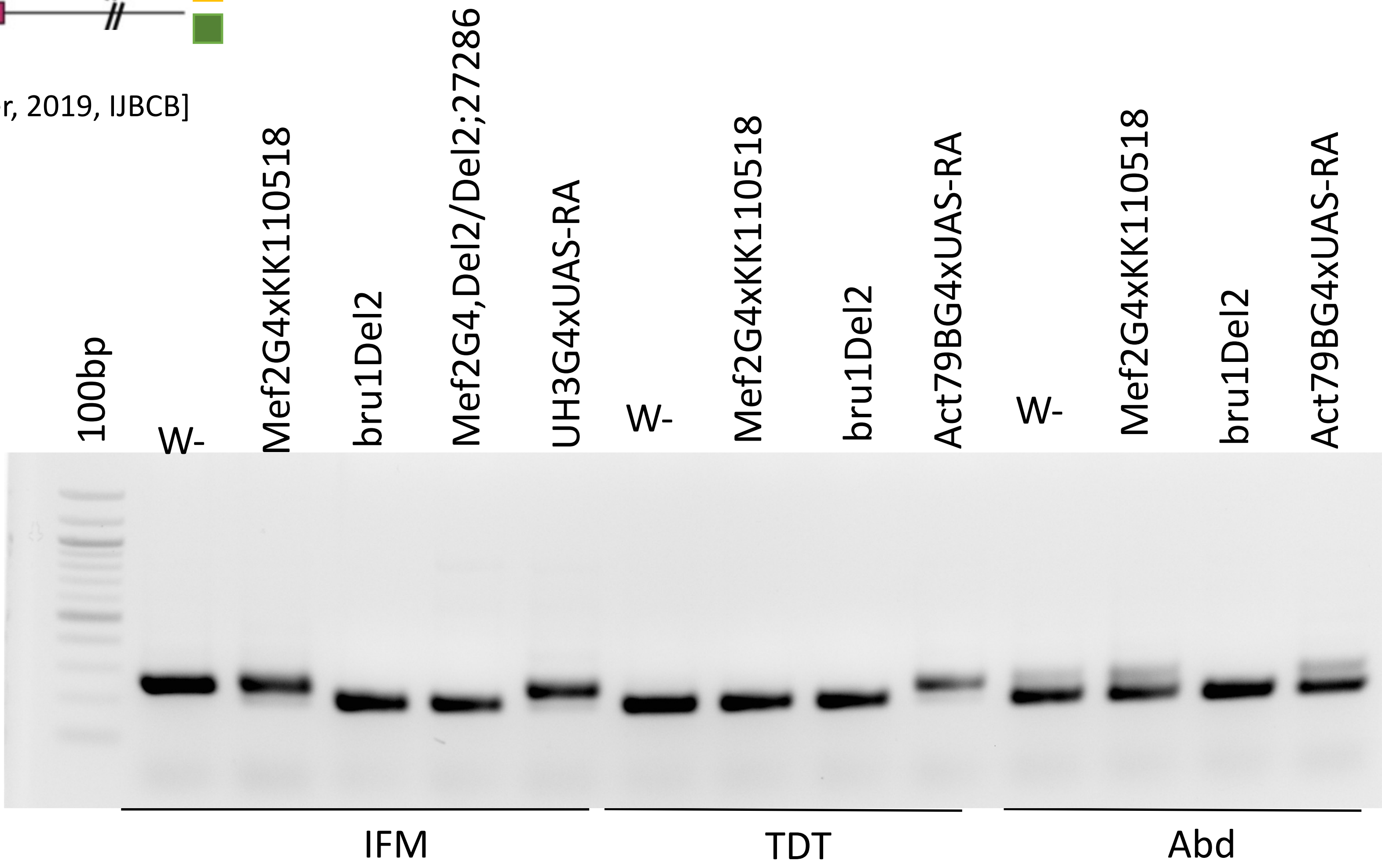

210108_1_wupA_1

WupA ex8

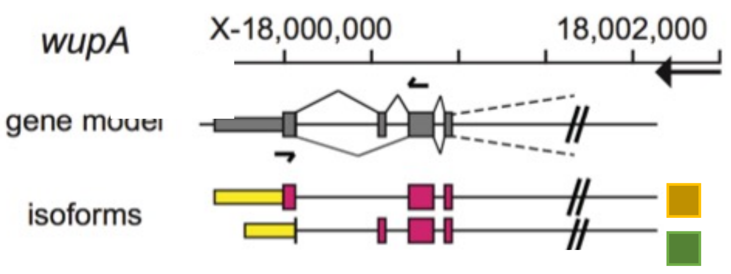

[Nikonova, Kao,..,Spletter, 2019, IJBCB]

Primers:
wupA
F: CGCTGAGTTCAACTTCCGCAACC
R: ATTGTTTAGGGCGGGAGTCACGG

Rp49 (at 500bp)
F: TCCTACCAGCTTCAAGATGAC
R: GTGTATTCCGACCACGTTACA

Seq from Orfanos et al. 2012

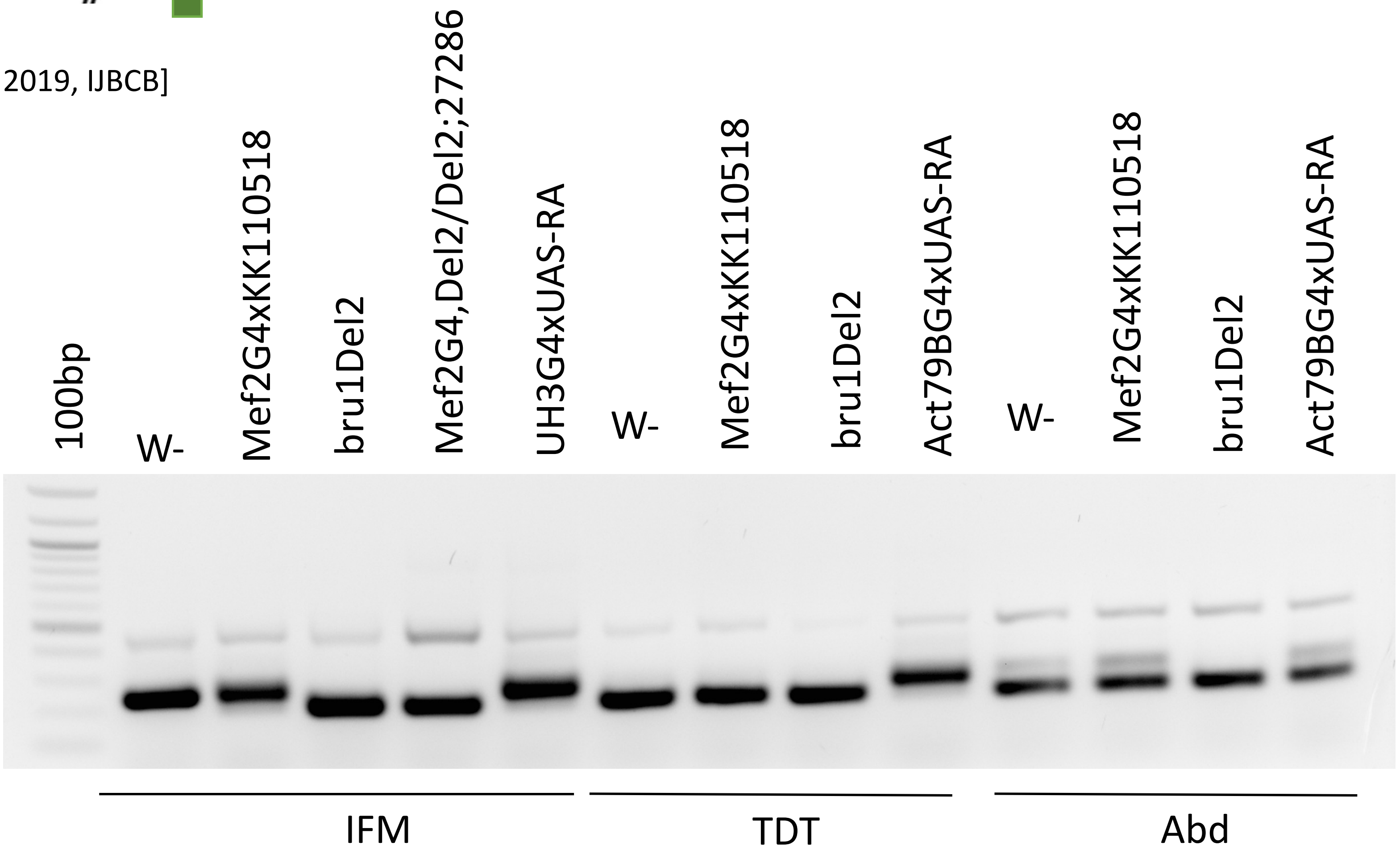

210108_2_wupA_1

WupA ex8

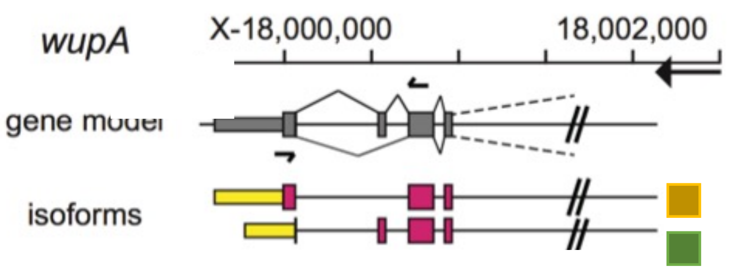

[Nikonova, Kao,..,Spletter, 2019, IJBCB]

Primers:
wupA
F: CGCTGAGTTCAACTTCCGCAACC
R: ATTGTTTAGGGCGGGAGTCACGG

Rp49 (at 500bp)
F: TCCTACCAGCTTCAAGATGAC
R: GTGTATTCCGACCACGTTACA

Seq from Orfanos et al. 2012

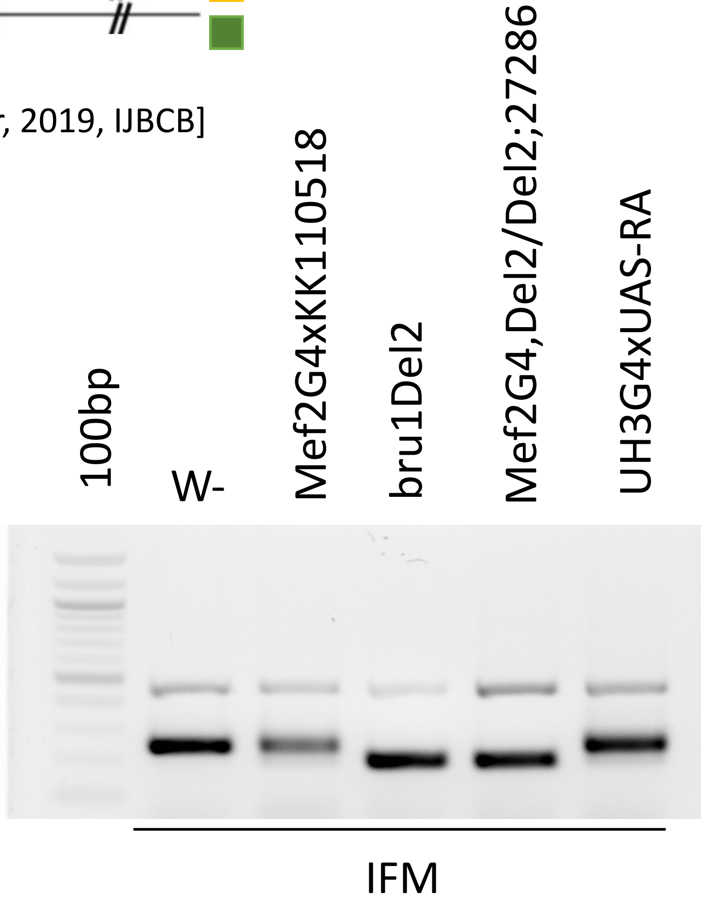

Gel: 210111_1

IFM (244 bp) band

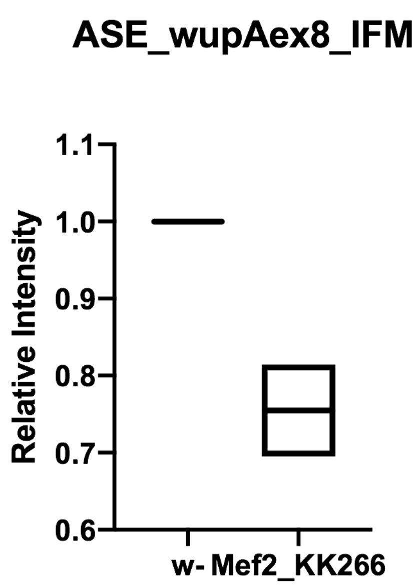

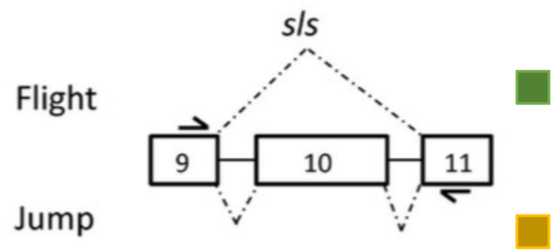

[Oas et al., 2014, JCB]

Primers for sls:

F: CGCGCAGTATGTGCAAAAT

R: AAACCGTTCCACGAAAAGTG

From Oas et al., 2014

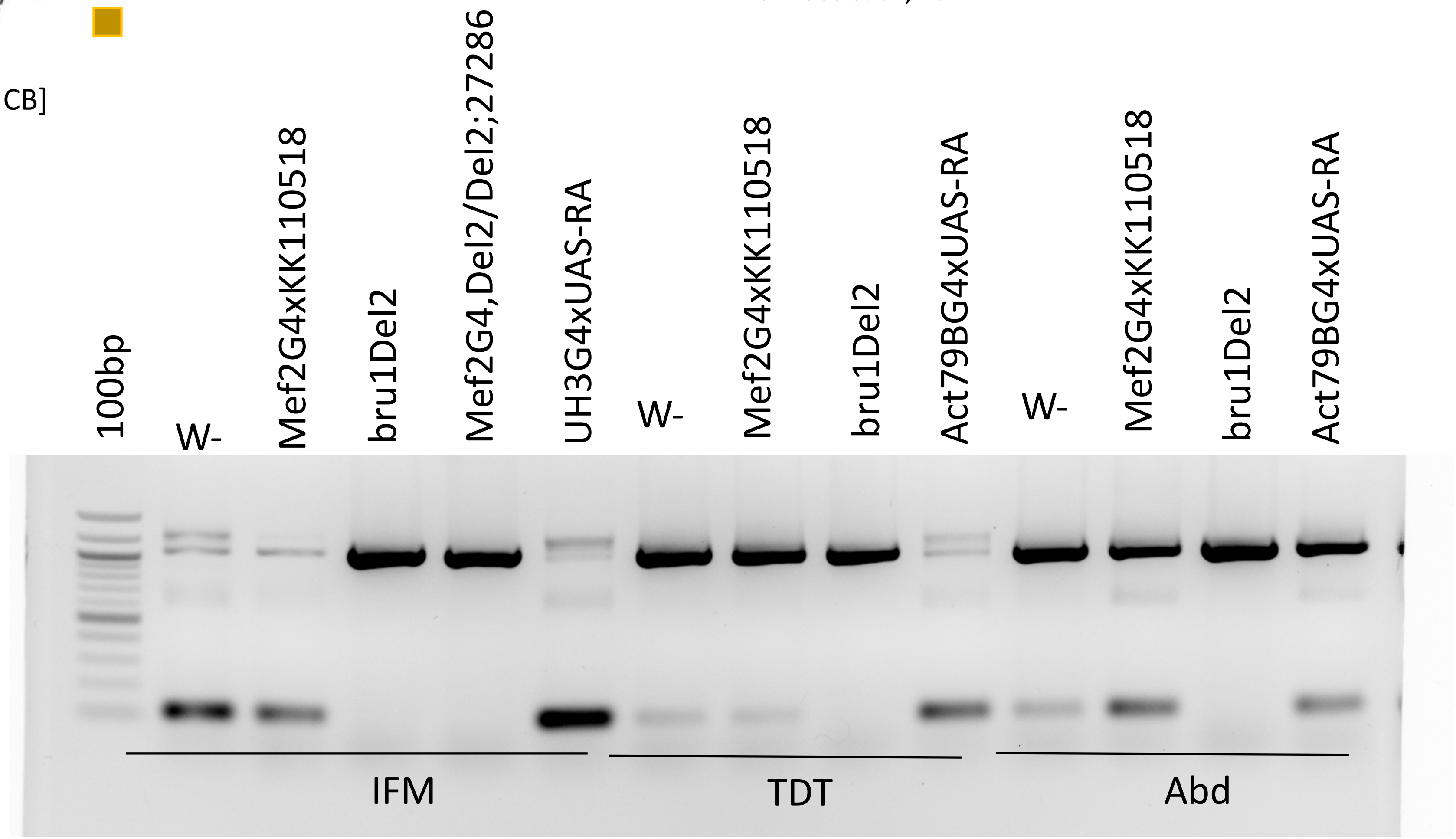

Gel: 201229_sls_AS_560ms

Strn-Mlck isoR

Primers:
Strn-Mlck
F: GTTGGGTATCTACGATCTCACAGG
R: CACGAAGGACATTACCCAATCGG

Rp49
F: GGTATCgacaacagagtgcg
R: GAACTTCTTGAATCCGGTGGG

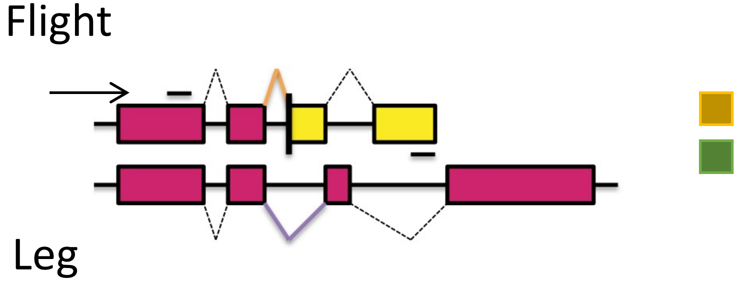

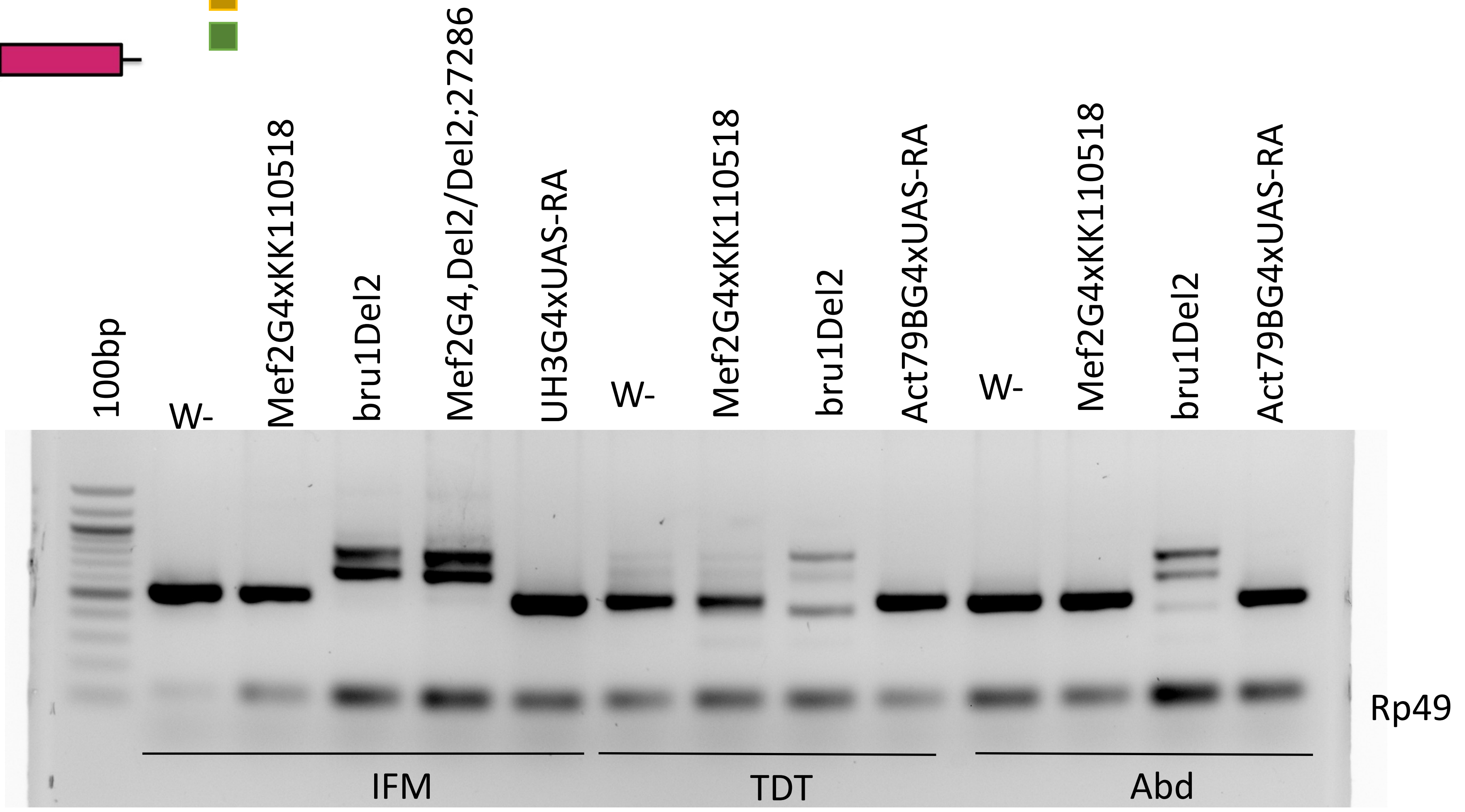

210104_Strn_AS_2
