# Supplementary material for: Rbfox1 is required for myofibril development and maintaining fiber type–specific isoform expression in Drosophila muscles
Source: Life Sci Alliance. 2022 Jan 7;5(4):e202101342. doi: 10.26508/lsa.202101342 (PMC8742874; doi:10.26508/lsa.202101342)
Supplement: Supplementary file 15 [file LSA-2021-01342_SdataF7.pdf]

Raw data used to generate plots

Figure panel

**7A**	Mef2 in Rbfox1 OE	
	IFM	
	control	Mef2 > Rbfox1
	1	0.5274417
	1	0.5651801
	1	0.3522362

**7G**	control	DLM Rbfox1-RNAi	controlTDT	TDT Rbfox1-RNAi	AbdM control	Abdm Rbfox1-RNAi
	1	2.8	0	0	1	5.76
	1	2.435	0	0	1	6.39

**7H**	IFMw-	IFMFoxIR	IFMFoxKD27286	FMDcr2FoxKD2728	FMFoxKDKK10151	TDTw-	TDTFoxIR
	1	2.037471	1.10451893	0.64176109	0.265050134	1	0.194
	1	1.74331	1.10811746	0.67017589	0.273470749	1	0.8309663
	1	2.058559	1.14425358	0.71411467	0.261182344	1	0.4232228
	1		1.2607474				

TDTFoxKD27286	DTFoxKDKK10151	Abdw-	AbdFoxKD27286	bdFoxKDKK101518
0.53085212	0.71705485	1	0.97792493	1.10054085
0.71432667	0.5895926	1	0.95345478	1.07565052
0.57380101	0.40661279	1	0.86983749	1.09013324
		1	1.03343451	1.05745802

**7G**	IFMsalmIR	TDTsalmIR	AbdsalmIR	IFMctl	TDTctl	Abdctl	IFMsalmIR29C
	1.03987751	0.87521706	0.93611373	1	1	1	0.1422914
	0.69996851	0.56968774	0.92086382	1	1	1	0.05140991
	1.06137105	0.41112268	1.00693225	1	1	1	0.5675913
	0.71303961	0.70095462	0.98699576	1	1	1	

Original RT-PCR gels

Rbfox1 in *salm-IR* tissues

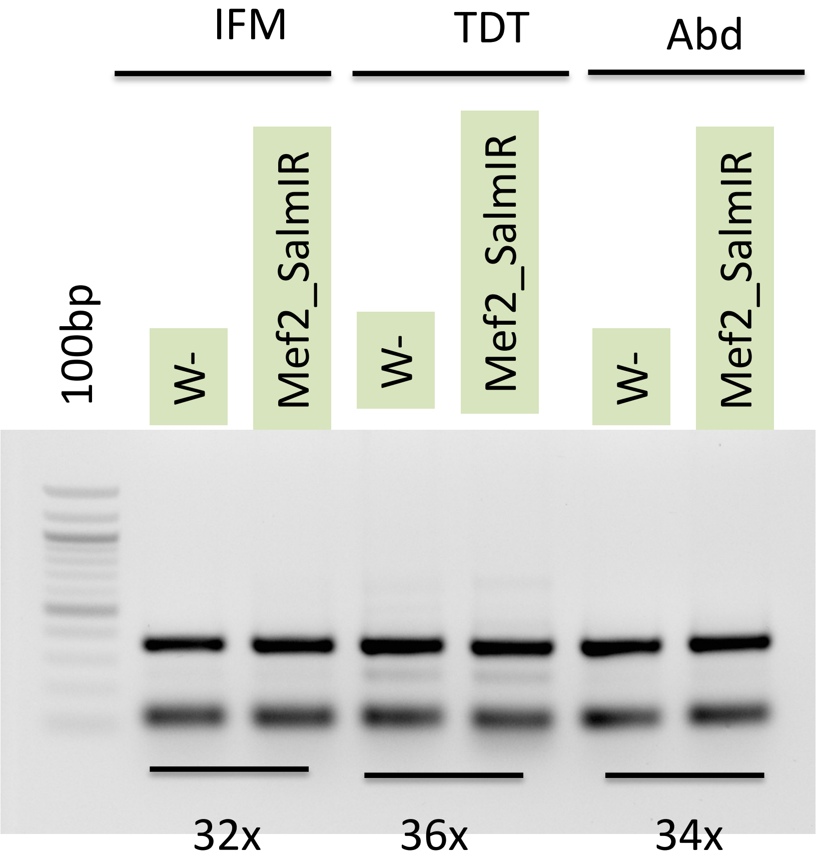

Gel: 210127

Rbfox1 in *salm-IR* tissues

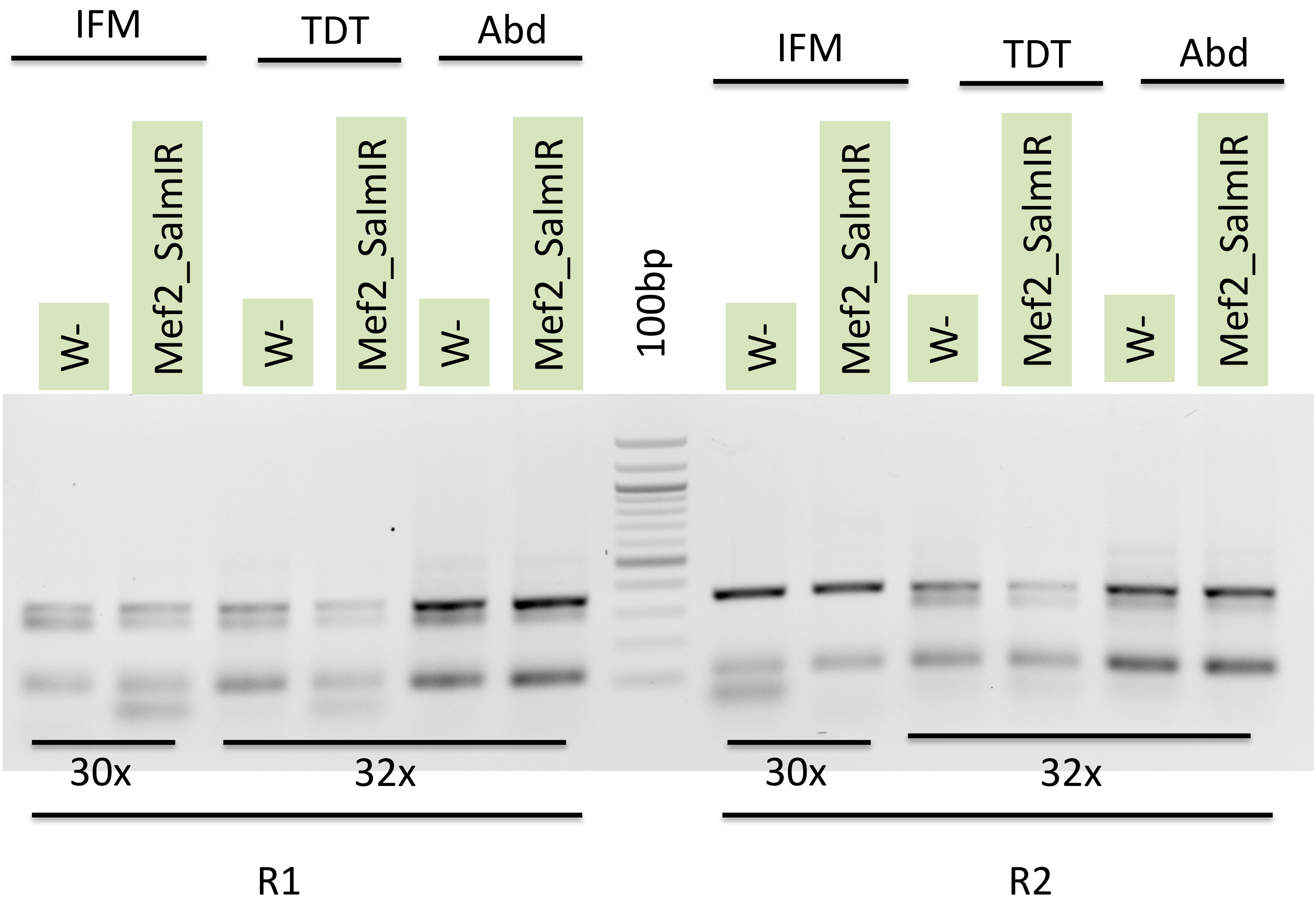

Gel: 210129_1

Rbfox1 in *salm-IR* tissues

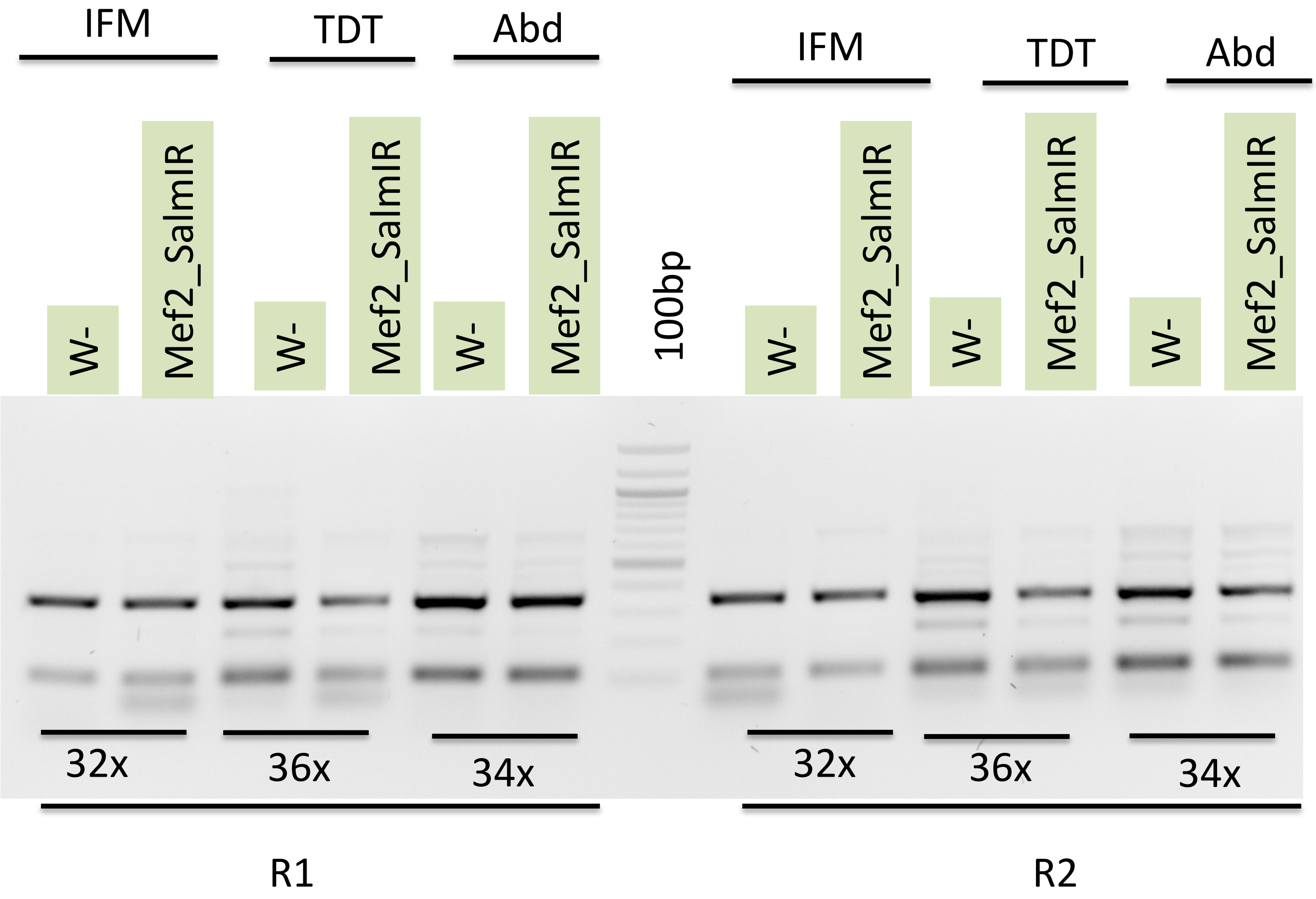

Gel: 210129_2

Rbfox1 in *salm-IR* tissues

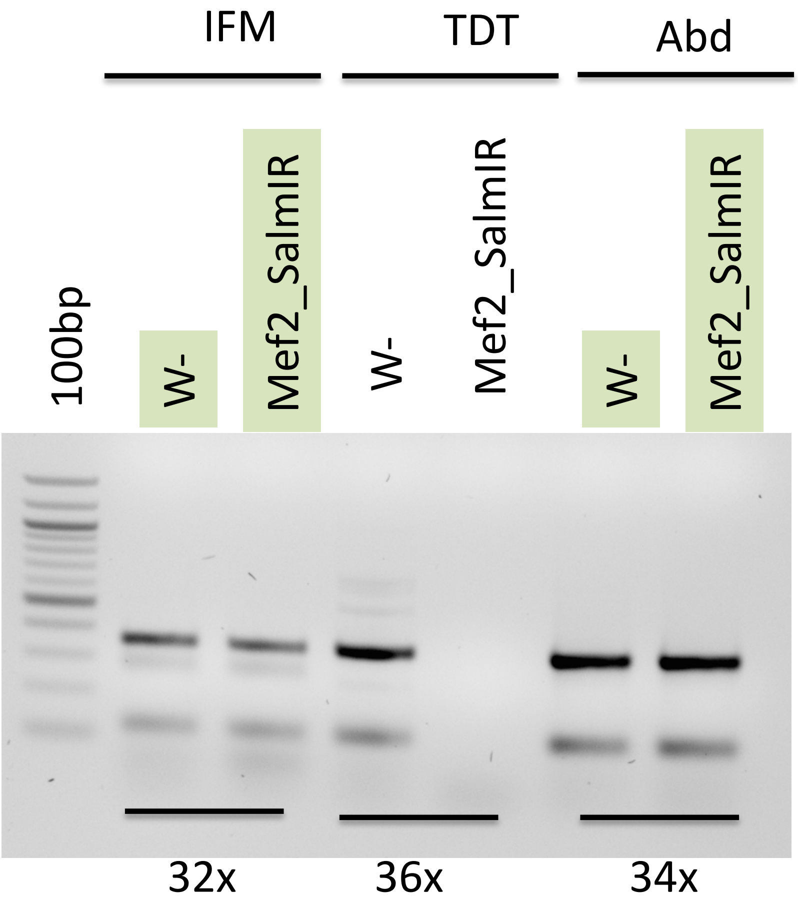

Gel: 210129_3

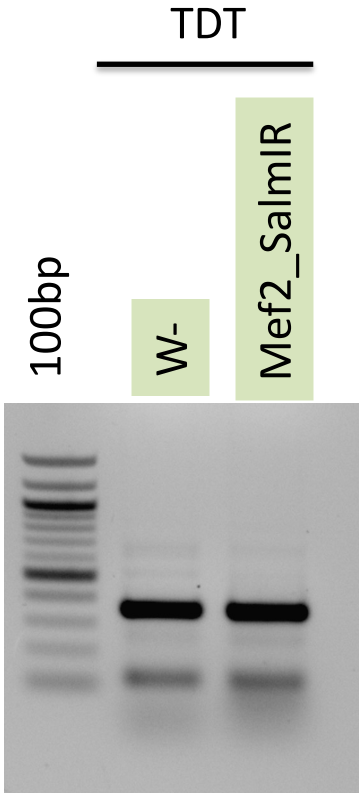

Gel: 210201

salm in *Rbfox1* knockdown tissues

IFM

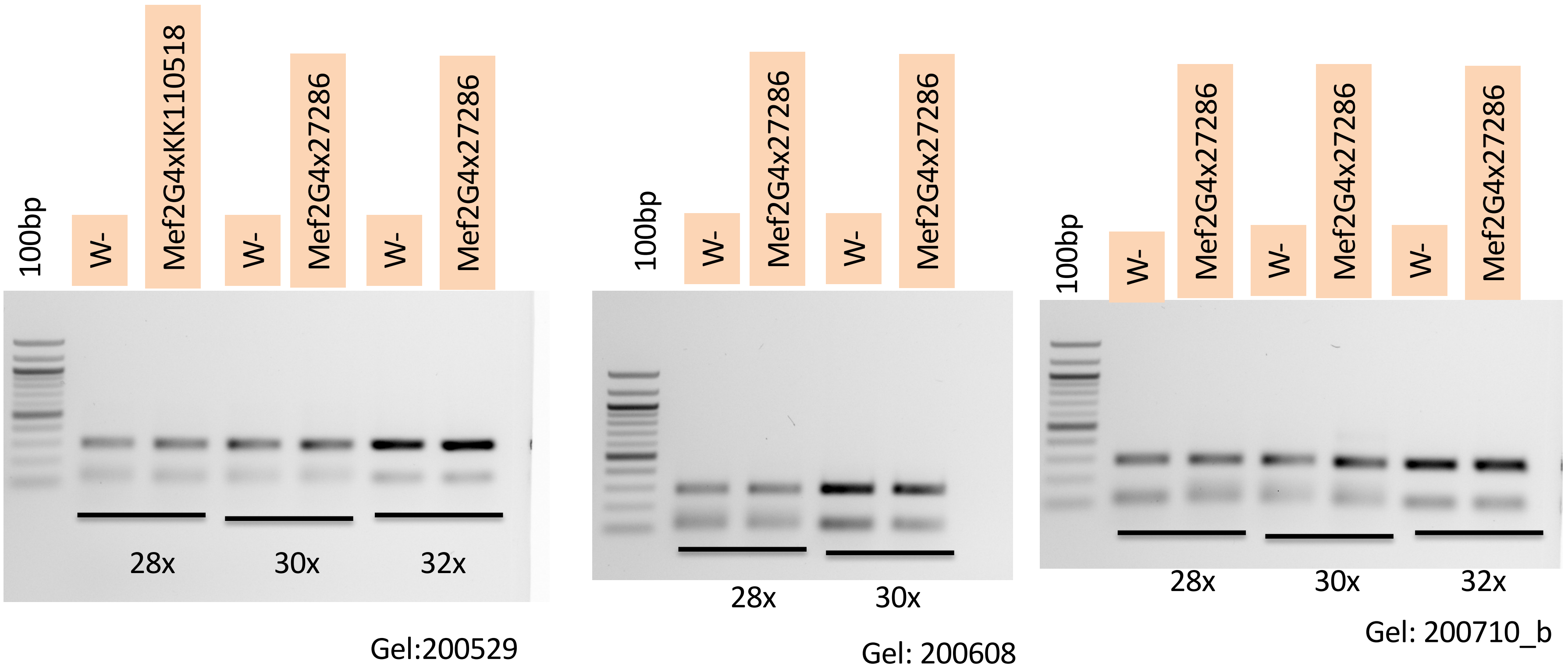

Nx of one gel = same PCR reaction, from which aliquots were taken at the respective cycle number

salm in *Rbfox1* knockdown tissues

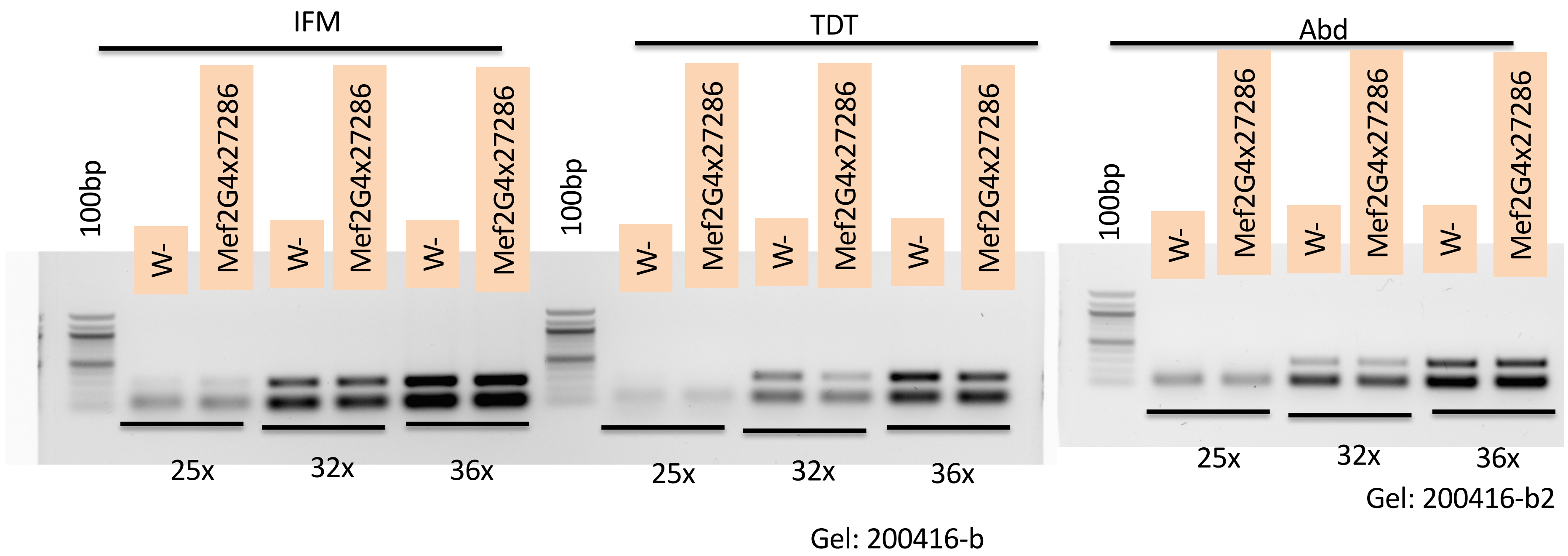

salm in *Rbfox1* knockdown tissues

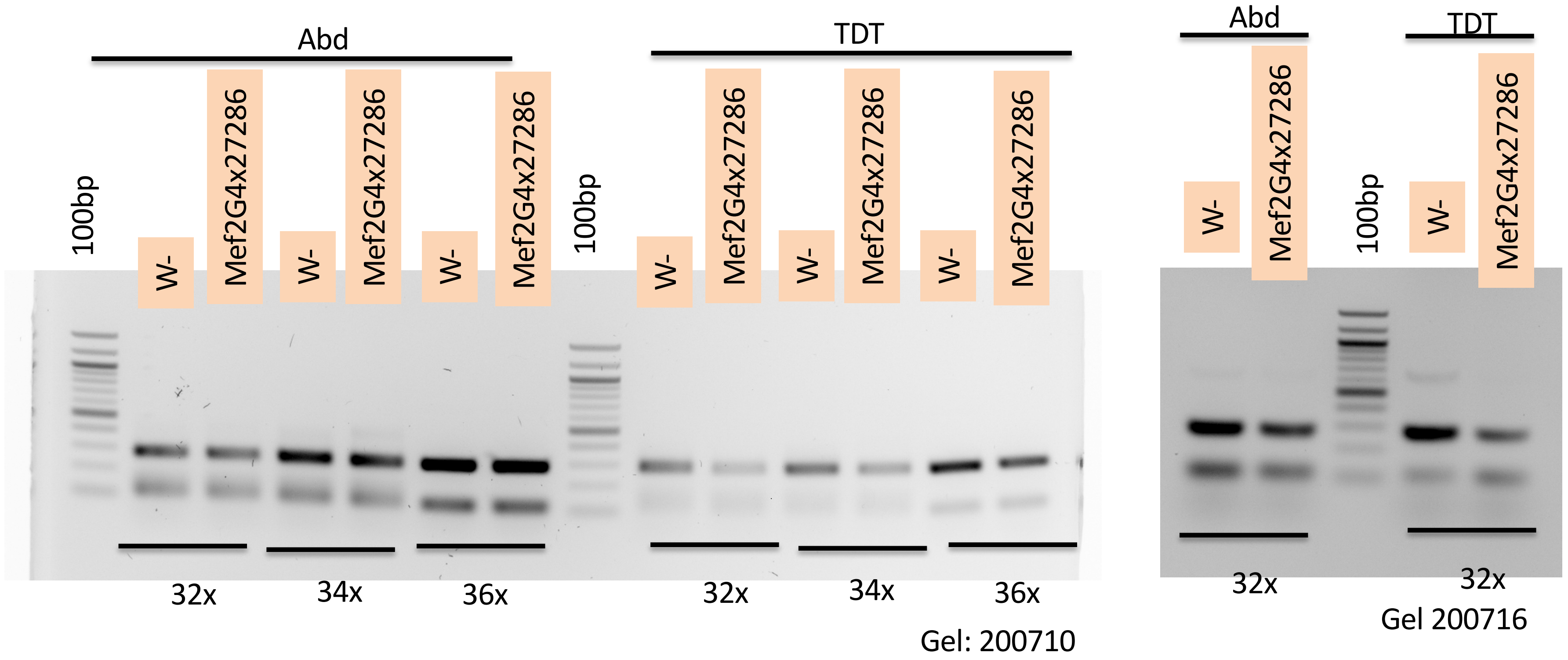

salm in *Rbfox1* knockdown tissues

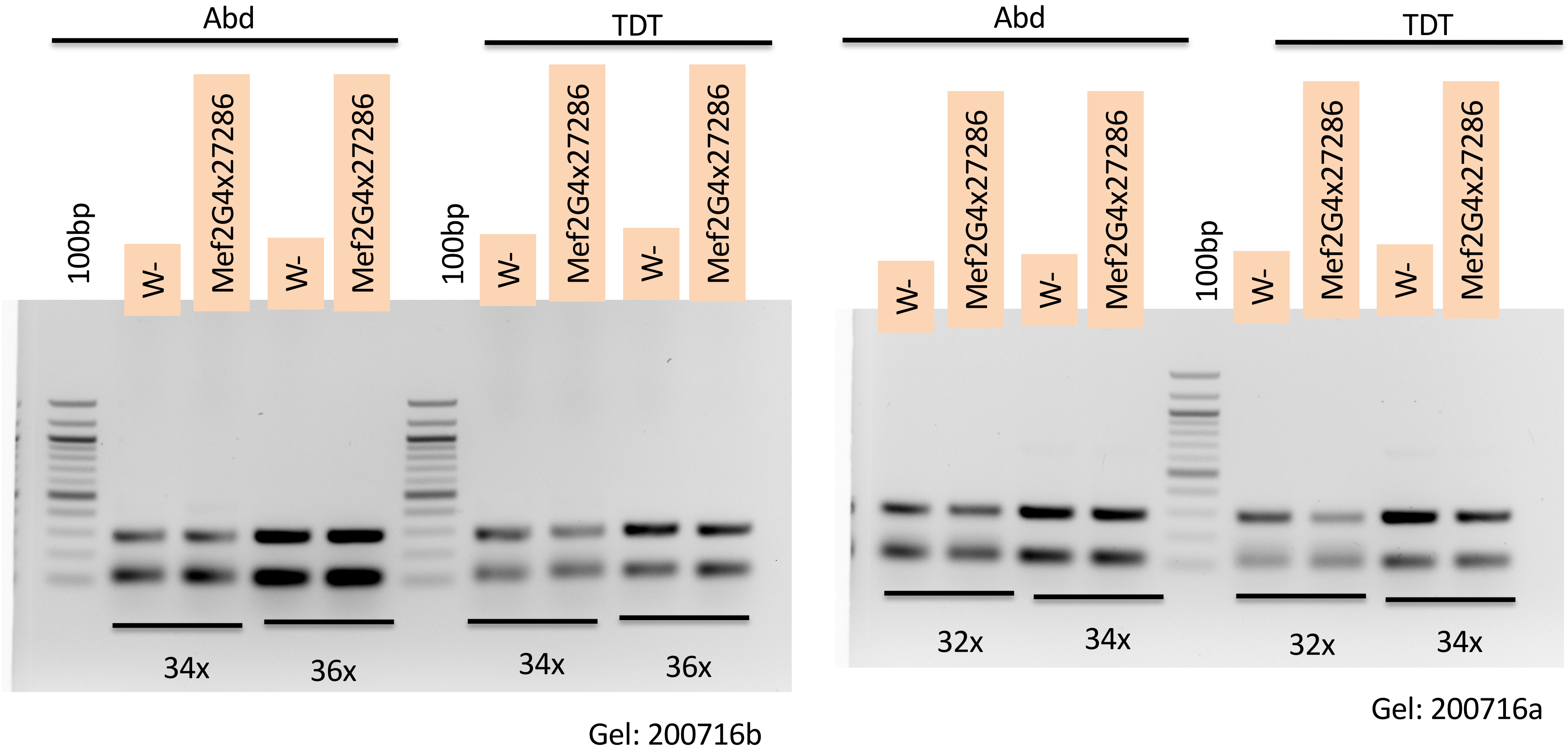

salm in *Rbfox1* knockdown tissues

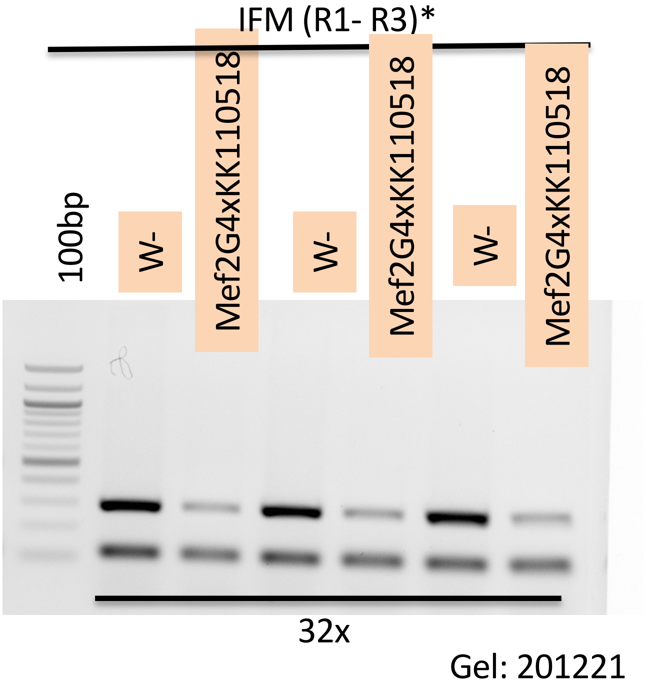

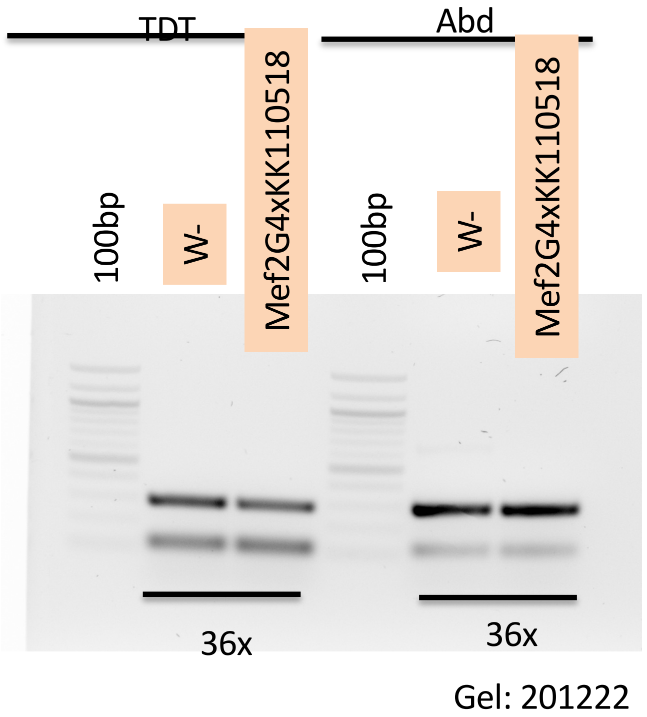

* 3 replicates done as different reactions (MMs), but runed on same PCR block/gel

salm in *Rbfox1* knockdown tissues

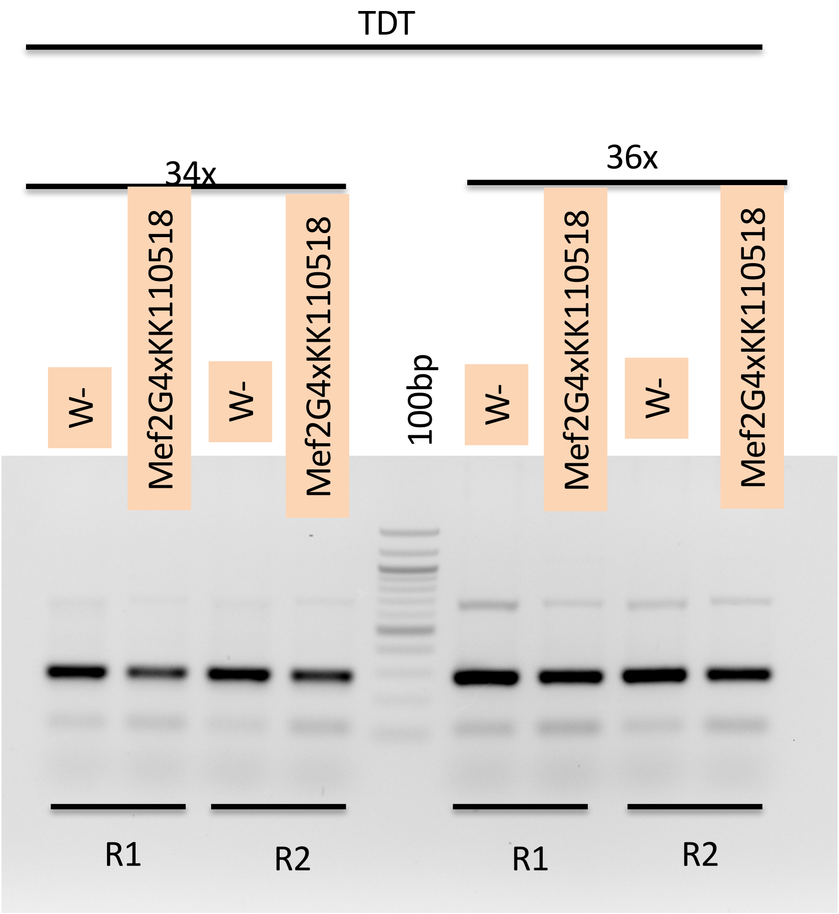

Gel: 201223_salm_TDT

salm in *Rbfox1* knockdown tissues

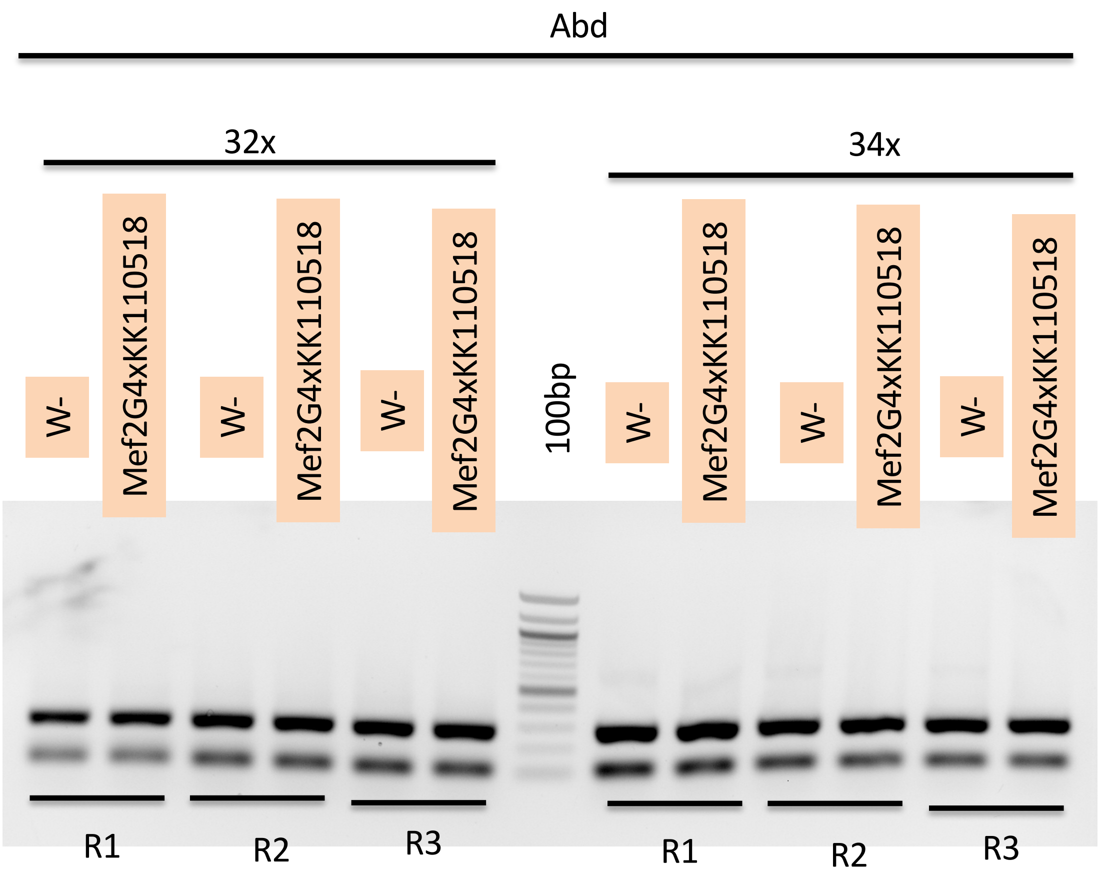

salm in *Rbfox1* knockdown tissues

All: 32x cycles

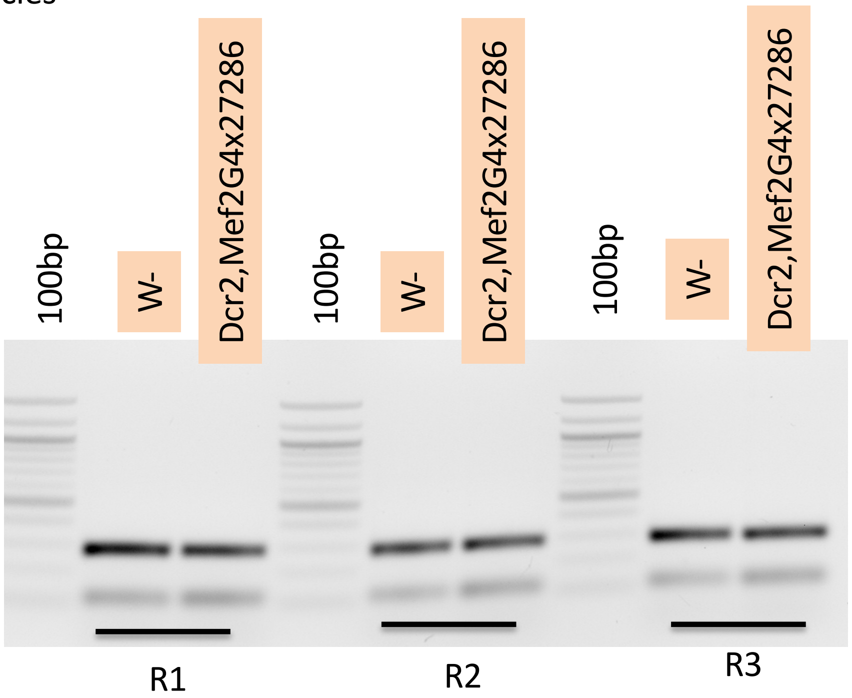

Gel: 210216_3
