# Supplementary material for: Rbfox1 is required for myofibril development and maintaining fiber type–specific isoform expression in Drosophila muscles
Source: Life Sci Alliance. 2022 Jan 7;5(4):e202101342. doi: 10.26508/lsa.202101342 (PMC8742874; doi:10.26508/lsa.202101342)
Supplement: Supplementary file 16 [file LSA-2021-01342_SdataFS7.pdf]

Raw data used to generate plots

Figure panel

		Mef2vstdt	Mef2vsleg	Mef2vssalm	w-vsM3	Mef2vsBruIR
**S7B**	pan (DESeq2)	0.145721	-0.248562	-0.3245191	0.984887	0.087116
	exon17 (DEXSeq)	-0.513707	-0.321576	0.19918577	-0.741052	-0.028972
	exon20 (DEXSeq)	2.12902	0.297947	-0.2680546	0.874541	0.428291
	exon21 (DEXSeq)	-1.081257	0.086587	0.25453964	0.288115	1.045288

		Promoter1	Promoter2	Promoter3
**S7C**	IFMmyo	0.09	0	0.91
	IFM16h	0.65	0.05	0.3
	IFM24h	0.69	0.11	0.2
	IFM30h	0.71	0.09	0.2
	IFM48h	0.6	0.3	0.1
	IFM72h	0.68	0.16	0.15
	IFM90h	0.56	0.18	0.26
	IFM1d	0.31	0.32	0.38
	leg30h	0.13	0.03	0.84
	leg50h	0.14	0.2	0.67
	leg72h	0.14	0.23	0.64
	leg1d	0.37	0.18	0.45
	jump1d	0.39	0.14	0.47

	IFMw-	IFMFoxIR	IFM27286	IFMDcr,27286	FMKK10151	TDTw-	TDTFoxIR	TDT27286	TDTKK101518
**S7D**	1	1.555092	1.2866	0.8567	0.3786	1	1.023671	1.176975	1.1460198
	1	1.213834	1.2408	0.7933	0.4414	1	0.728619	1.276915	0.7511658
	1	1.481729	1.8049	1.1199		1	0.933323	1.59987	1.3864816
	1		1.1015			1		1.799734	1.5202053

Original gels RT-PCR

Exd levels in Rbfox1 knockdown

Exd

IFM, 32x

100 bp
W-
Mef2_KK110518
Mef2_27286

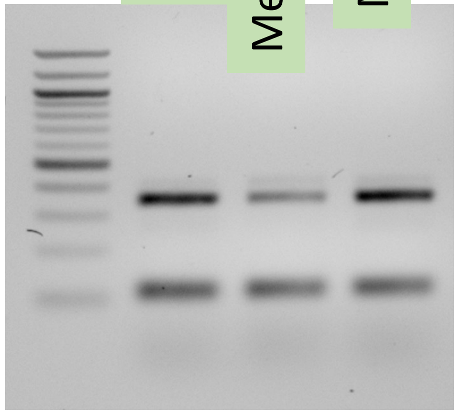

Exd

TDT, 36x

100 bp
W-
Mef2_KK110518
Mef2_27286

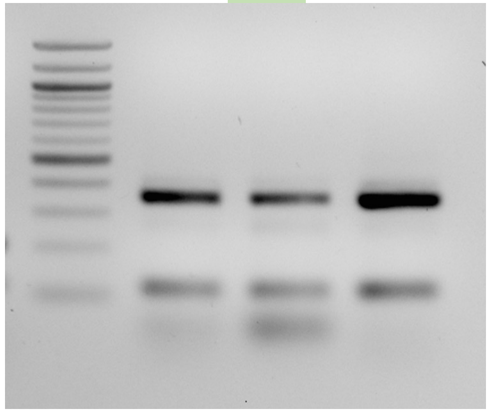

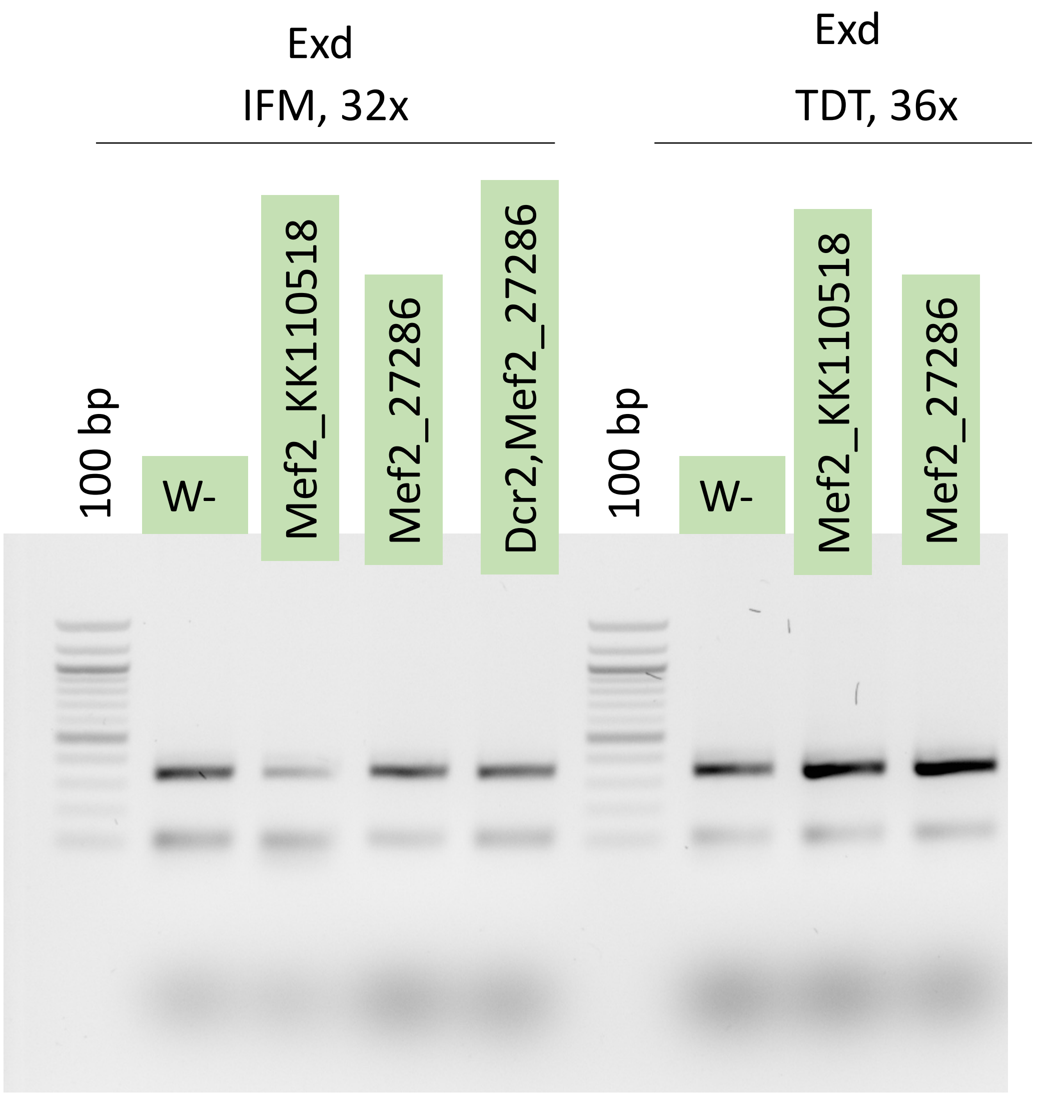
Exd
IFM, 32x
Exd
TDT, 36x
100 bp
W-
Mef2_KK110518
Mef2_27286
Dcr2,Mef2_27286
100 bp
W-
Mef2_KK110518
Mef2_27286

Gel:210212_1

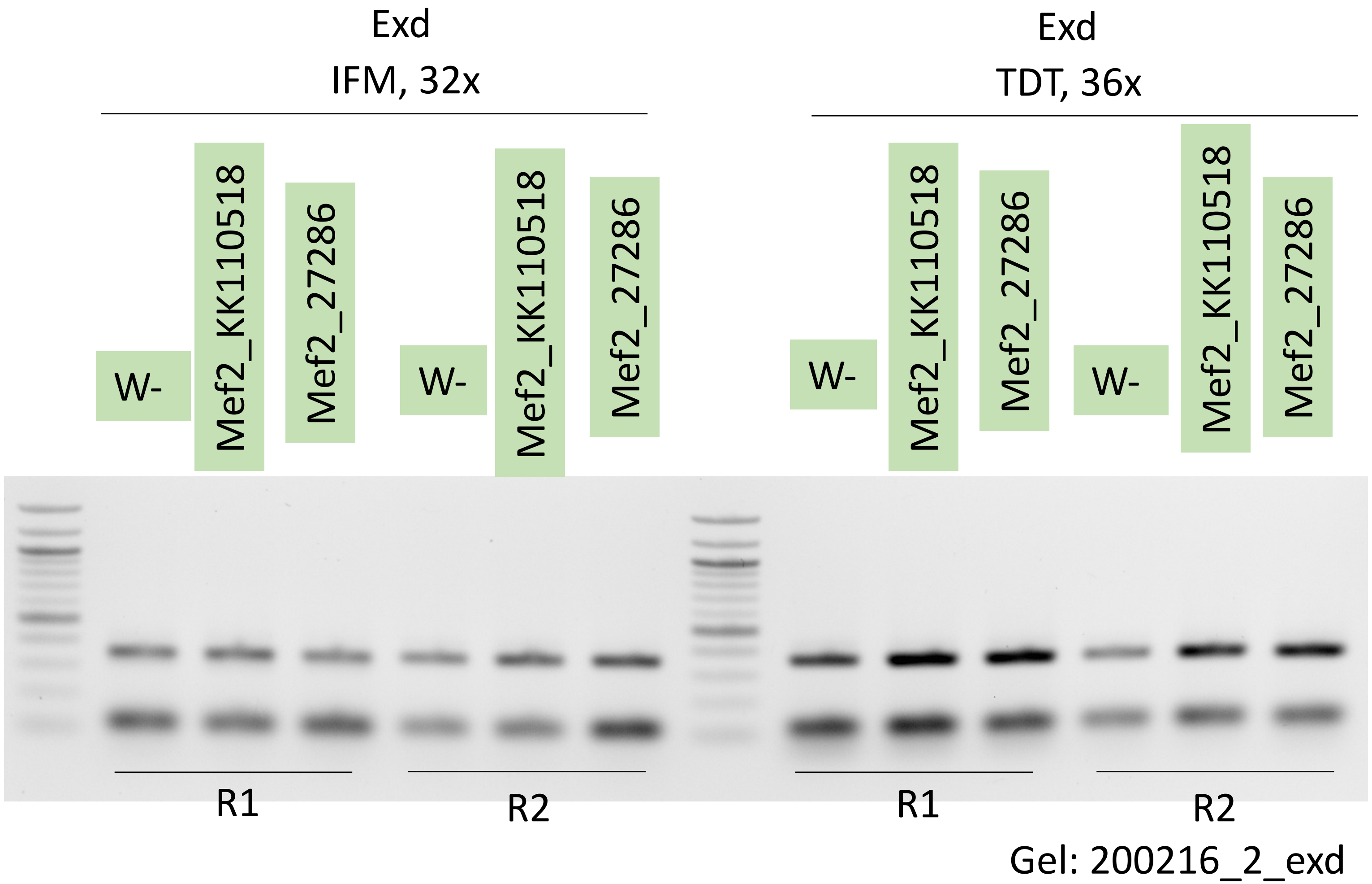
Exd
IFM, 32x
Exd
TDT, 36x
W-
Mef2_KK110518
Mef2_27286
W-
Mef2_KK110518
Mef2_27286
W-
Mef2_KK110518
Mef2_27286
W-
Mef2_KK110518
Mef2_27286
R1
R2
R1
R2
Gel: 200216_2_exd
